# How electronic and steric effects in acceptor alcohols shape S_N_1- and S_N_2-type glycosylation reactions

**DOI:** 10.1039/d5sc07201h

**Published:** 2025-11-17

**Authors:** Daan Hoogers, Koen N. A. van de Vrande, Dennis van der Meij, Wouter A. Remmerswaal, Coralie Tugny, Gijsbert A. van der Marel, Jeroen D. C. Codée

**Affiliations:** a Leiden University, Leiden Institute of Chemistry Einsteinweg 55 2333 CC Leiden The Netherlands jcodee@chem.leidenuniv.nl

## Abstract

The stereochemistry of glycosidic bonds dictates the glycan structure and function. Therefore, their selective introduction is crucial in the synthesis of oligosaccharides. This study systematically maps the influence of electronic and steric properties of alcohol acceptors on the glycosylation mechanism of the 4,6-*O*-benzylidene glucosyl donor, which has been shown to engage in glycosylation reactions spanning the whole breadth of the S_N_1–S_N_2 reaction continuum. Using kinetic isotope effects (KIEs), competition experiments and the determination of stereoselectivity as a function of acceptor concentration, we have quantified the influence of electronic and steric effects in the acceptor on the stereochemical outcome of the glycosylation reaction. In this way, we were able to pinpoint the mechanism on the S_N_1–S_N_2 continuum. Our findings reveal that α-glycosides can originate from S_N_1 and S_N_2 pathways, while the β-products are always generated through an S_N_2-like mechanism. Sterically hindered and electron poor acceptors favor the S_N_1-like pathway, and the stereoselectivity of the reaction can be controlled, in part, by adjusting the concentration of the acceptor.

## Abstract 2

Acceptor-driven shifts from S_N_2- to S_N_1-like glycosylation reactions are quantified through KIEs, competition experiments, and concentration effects, providing a concise quantitative framework for mechanism and selectivity.

## Graphical abstract

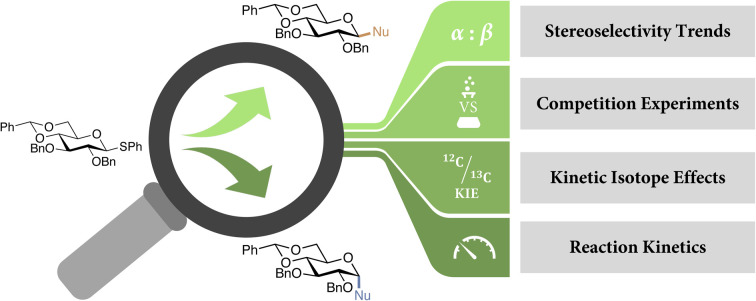

## Introduction

The central reaction in chemical oligosaccharide synthesis is the bond formation between two saccharide building blocks, *i.e.* the glycosylation reaction.^1–3^ Glycosidic bonds connecting monosaccharides can either exist as 1,2-*trans* or 1,2-*cis* diastereomers and the nature of the linkage has a profound influence on the structure and function of glycans. A common approach to synthesize glycosidic bonds is a nucleophilic substitution reaction between a glycosyl electrophile carrying a triflate leaving group^4^ at the anomeric position (the donor), and a nucleophilic alcohol (the acceptor). Controlling the stereoselectivity in glycosylation reactions remains a significant challenge and the outcome of a glycosylation reaction is affected by the interplay of multiple factors, including the reactivity of the glycosyl donor^5–10^ and acceptor.^11–13^ The reactivity of the donor and acceptor is shaped by their decoration pattern and is crucially influenced by the functional and protecting groups present on the building blocks.

Systematic reactivity studies have provided detailed insight into structure–reactivity–stereoselectivity relationships,^12–15^ and these studies allow for an understanding of how changes in the donor and acceptor structure can impact reaction outcomes. To map the reactivity of donor glycosides, competition reactions can be used in which two building blocks compete for a limiting amount of activator, establishing relative reactivity values (RRVs).^9,16,17^ Similarly, competition reactions can be used to map the reactivity of acceptor nucleophiles^18^ by making them compete for a limited amount of electrophile.^19,20^

**Fig. 1:**
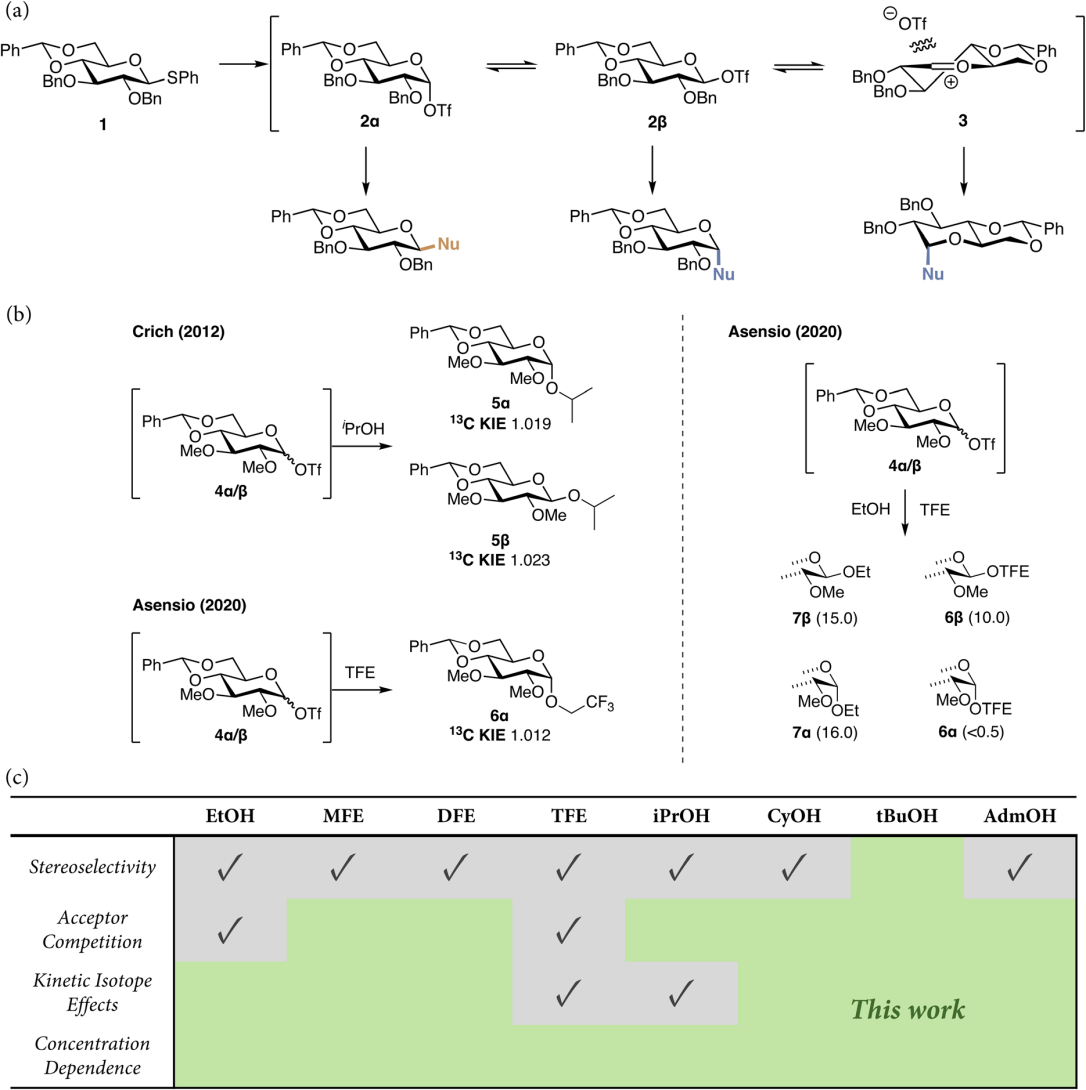
(a) Mechanistic pathways to account for the selectivity in glycosylation reactions of the 4,6-*O*-benzylidene glucosyl donor, 1. (b) Kinetic isotope effects (KIEs) for ^i^PrOH were recorded by Crich and coworkers on a similar, 2,3-di-*O*-methyl protected, glucosyl sulfoxide donor.^24^ Acceptor competition experiments and KIE measurements for TFE were performed by Asensio and colleagues.^19^ Numbers in brackets are relative formation rates for the *O*-glycosides in the competition experiments. (c) An overview of experiments performed in recent times to probe the mechanistic picture and the experiments that we present in this work.

We have recently found that the stereoselectivity of glucosylation reactions using 4,6-*O*-benzylidene protected glucosyl donors critically depends on the reactivity of the acceptor alcohol. As depicted in Fig. 1a, activation of 4,6-*O*-benzylidene protected glucosyl donor 1 leads to the formation of the α-triflate 2α, which can be detected by low-temperature NMR spectroscopy. α-triflate can epimerize by substitution with external triflate anions to provide the less stable and more reactive β-triflate 2β. This species cannot be observed using conventional NMR techniques, but through ^13^C-labeling Asensio and co-workers have been able to identify this species (4β, Fig. 1b).^19^ Subsequently, White and Boltje and co-workers have used chemical exchange saturation transfer (CEST) NMR-techniques,^21–23^ to not only show the presence of this fleeting intermediate but also, through ^19^F exchange NMR (EXSY), to determine the exchange rate of the anomeric triflates. The covalent triflates may also dissociate to provide the corresponding oxocarbenium ion-triflate pairs (3), but the lifetime of these species in solution is too short to detect them. Gas phase studies in the absence of the triflate anion have been used to characterize the glucosyl cation and various computational approaches have been used to understand its reactivity.

To account for the stereoselectivity observed in the glycosylations of donor 1, it has been postulated that reactive nucleophiles can directly displace the α-triflate, while less reactive nucleophiles require a more reactive electrophile such as the β-triflate or oxocarbenium ion. Through the use of a systematic series of partially fluorinated ethanols, we have mapped how the stereoselectivity of the reactions of donor 1 gradually shifts from β- to α-selectivity as a function of the electron density on the acceptor.

While the detection of (low abundant) reactive intermediates and the mapping of structure–reactivity–stereoselectivity relationships have allowed the sketching of the S_N_1–S_N_2 reaction continuum of glycosylation reactions of 1, it remains challenging to dissect where on the continuum a particular donor–acceptor pair will react. Kinetic isotope effects (KIEs) have been employed to dissect (enzymatic) reaction mechanisms and used to pinpoint glycosylation reaction mechanisms. Crich and coworkers determined primary ^13^C KIEs for the formation of the α- and β-products of the reaction of benzylidene glucosides and iso-propanol to reveal that both products 5α and 5β were formed in reactions with significant S_N_2-character (see Fig. 1b).^24^ Additionally, Asensio and co-workers used a similar approach to show that the reaction of trifluoroethanol (TFE) and benzylidene protected glucosyl triflates, 4α/β, takes place on the S_N_1-side of the spectrum (Fig. 1b).^19^

As the reactivity of a typical carbohydrate acceptor lies somewhere in between the reactivity of monofluoroethanol (MFE) and TFE, we set out to map KIEs for the full series of partially fluorinated ethanol acceptors, to map where along the S_N_1–S_N_2 reaction continuum the substitution reactions of these acceptors take place. We have expanded this set of model acceptors with a subset of alcohols that can report the effect of increasing steric bulk in the nucleophile, and have included iso-propanol, cyclohexanol, *tert*-butanol and adamantanol in our studies. Of note, adamantanol has previously been shown to react in an α-selective fashion with 4,6-*O*-benzylidene protected glucosyl sulfoxides,^25^ which is remarkable as it can be regarded to be a relatively electron rich nucleophile. Besides the determination of KIE values for the glycosylations of donor 1 with the set of model acceptors, we have probed their relative acceptor reactivity (RAR) through competition experiments, and we have determined how the stereoselectivity of the glycosylations changes upon changing the concentration of the acceptors. These studies have shown that RAR strongly depends on the substitution reaction mechanism, with RAR in S_N_2-type glycosylations being significantly more sensitive to the nature of the acceptor than in S_N_1-type reactions. Strikingly, more electron poor acceptors are shown to react faster in S_N_1-type glucosylation reactions. We also show that the stereoselectivity of the glucosylation reaction can be controlled, if the substitutions take place with sufficient S_N_1-character, by changing the concentration of the reactions.

## Results and discussion

### Structure–stereoselectivity relationships

The toolset of model *O*-nucleophiles that we used in this study is shown in Table 1 and it encompasses ethanol (EtOH), 2-monofluoroethanol (MFE), 2,2-difluoroethanol (DFE), and 2,2,2-trifluoroethanol (TFE) to map how electronic effects influence the glycosylation reaction mechanism, and iso-propanol (^i^PrOH), cyclohexanol (CyOH), *tert*-butanol (^*t*^BuOH) and 1-adamantanol (AdmOH) to probe the influence of acceptor size on the glycosylation outcome. The stereoselectivity of the glycosylation of all these acceptors is reported in Table 1, clearly revealing how electronic and steric effects impact the stereochemical outcome. As previously described, the series of partially fluorinated ethanols shows the direct relationship between the electron density on the nucleophile and the α/β-product ratio, with the most reactive acceptors providing the β-glucosides and the more electron-poor nucleophiles leading to the α-products. The series of gradually larger nucleophiles show how the glucosylation reactions also become more α-selective with the increasing size of the acceptors, although the effect of acceptor size seems less pronounced than the effect imposed by the electronically deactivating groups.

**Table 1:** Model glycosylation reaction results from the 4,6-*O*-benzylidene glucosyl donor with both toolsets of model *O*-nucleophiles, respectively varying in nucleophilicity and size. The stereoselectivity of the reaction is expressed as α : β and based on ^1^H-NMR of purified α/β-product mixtures. Pre-activation-based glycosylation conditions were used: donor 1 (1.0 equiv.), Tf_2_O (1.3 equiv.), Ph_2_SO (1.3 equiv.), TTBP (2.5 equiv.), DCM (0.05 M), −80 to −60 °C, then add the nucleophile (2.0 equiv.) at −80 °C and allow to warm to −40 °C

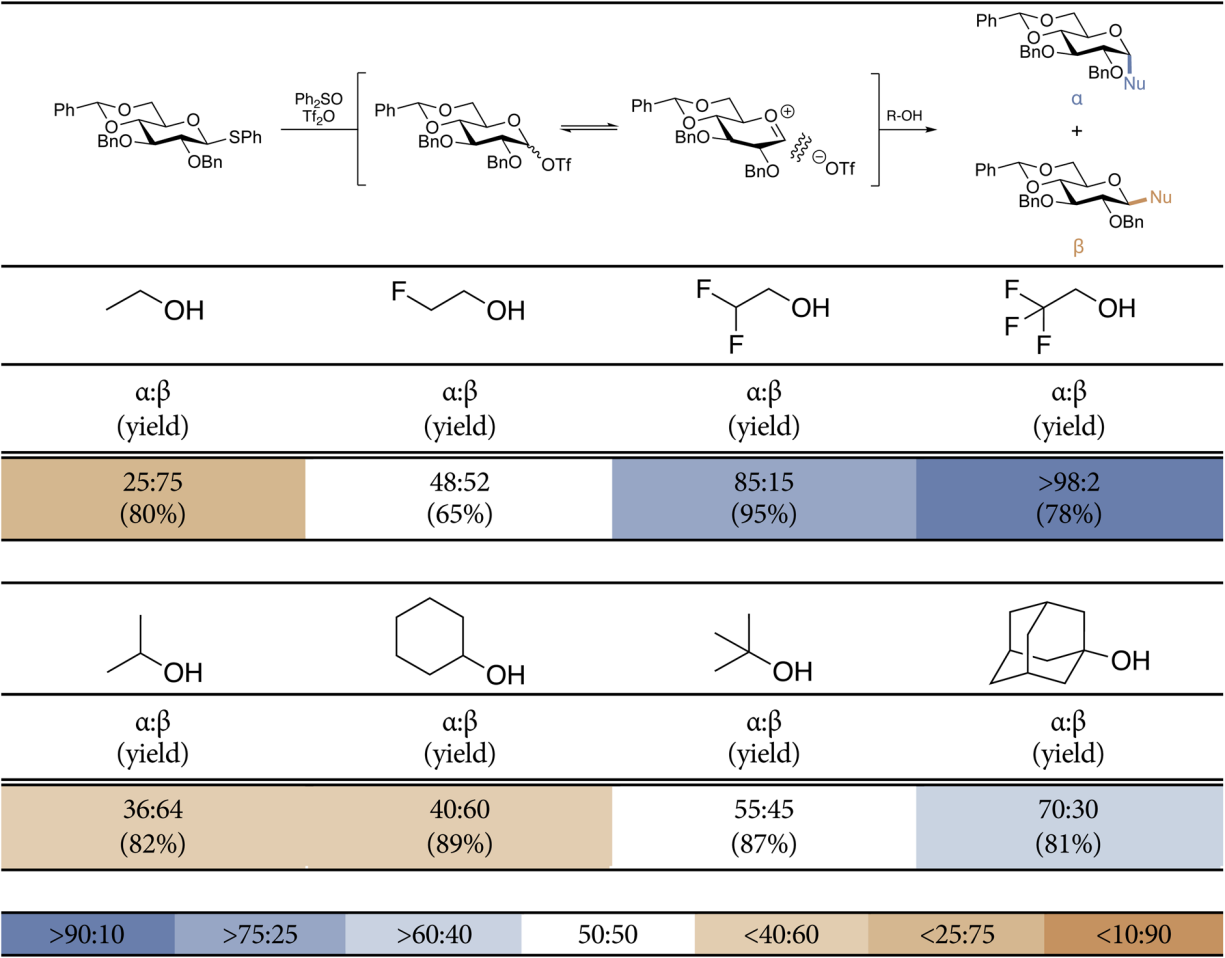

### Competition experiments

**Fig. 2:**
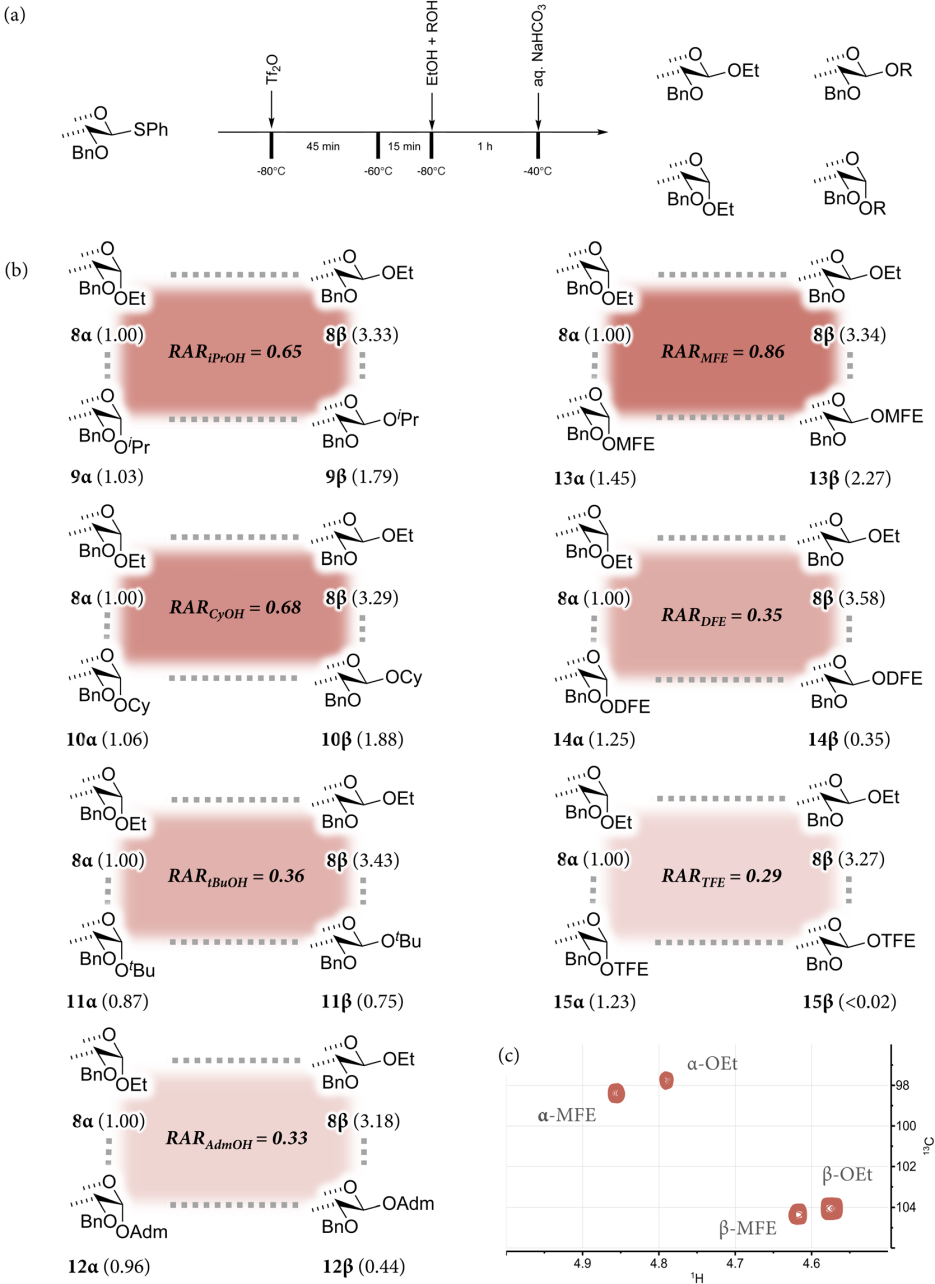
(a) Competition experiments performed with the 4,6-*O*-benzylidene glucoside donor, 1, and the sets of electronically and sterically varying acceptors. (b) Relative formation rates, shown with respect to α-*O*-ethyl glucoside. The apparent RRV values, shown in the center of each square, were calculated by adding up both α and β species. (c) An example of a quantitative 2D HSQC experiment for the competition between EtOH and MFE, allowing for the accurate determination of relative conversions towards the four different products.

To match the observed stereoselectivities to the relative reactivity of the acceptors, we next set out to probe the acceptors in a series of competition experiments, in line with the studies of Asensio and coworkers.^19^ In these experiments, the mixture of glucosyl triflates, derived from 4,6-*O*-benzylidene glucosyl donor 1, was treated with an 1 : 1 mixture of acceptors, using ethanol as the reference acceptor across all experiments (Fig. 2a). To prevent triflation of the acceptor species under these conditions, only 0.5 equiv. Tf_2_O was used in the activation, to ensure all Tf_2_O was consumed in the activation of the donor. In the experiments two equivalents of each acceptor were used to minimize the effect of depleting the reaction by one of the acceptors while the glycosylation progresses. Each reaction can give a mixture of four glycosylated products, and the relative yields reflect the kinetics of their formation. To establish the relative amount of formation of the products, the crude reaction mixtures were analysed by quantitative ^13^C-NMR to provide the product ratios as the ^1^H NMR spectra were too congested. Quantitative 2D-HSQC measurements were also recorded (Fig. 2b) to ensure that overlapping signals did not obstruct interpretation of the data (see Fig. 2c for a representative example). The relative amounts of the formed product with respect to α-*O*-ethyl glucoside are reported in Fig. 2b. Overall apparent RAR values were calculated for each of the acceptors by comparing the total amount of α- and β-products formed from the acceptors with respect to the total amount of formed α/β-ethyl glucosides. As described below, this value only provides a rough estimate of the RAR, as the relative nucleophilicity depends on the glycosylation reaction mechanism.

On analysing the competition experiment data, several trends become apparent. First, the α : β ratio of the ethanol reference products remains nearly constant across the experiments, indicating that the reactivity of EtOH is unaffected by the presence of other competing acceptors, and that the available reaction pathways for both acceptors do not influence one another. In the series of sterically hindered acceptors, the ratio of formed α-products is similar across the competition experiments (8α*vs.*9α, 10α, 11α and 12α), while the formation of the β-products decreases as the size of the acceptor increases (8β*vs.*9β, 10β, 11β and 12β). This implies that the substitution of the α-triflate is more sensitive to steric hindrance than the substitution of the β-triflate. Because the α-triflate is more stable than its β-counterpart, it may be substituted in a reaction having a later S_N_2-transition state, in which the nucleophile is closer to the anomeric carbon, thus imposing relatively stronger steric interactions for the larger acceptors.

From the competition experiments of the set of fluorinated ethanol acceptors, an important difference between the α- and β-product forming pathways becomes apparent. The rate of formation of the β-products strongly decreases with decreasing nucleophilicity (8β*vs.*13β, 14β and 15β) while the ratio of the α-products (8α*vs.*13α, 14α and 15α) shows that more product is formed with decreasing nucleophilicity. This may be the result of a developing stabilizing hydrogen bonding interaction between the incoming nucleophile and the C2–O-atom group in glucosyl triflate, which assists in the formation of the α-products. The alcohol functionalities of the weaker nucleophiles will be stronger H-bond donors and benefit more from this interaction. While further studies will be needed to confirm this hypothesis, H-bonding between donor–acceptor pairs has often been suggested and can be exploited in stereoselective H-bond guided glycosylation strategies.^15,26–28^

Overall, the competition experiments have revealed that the pathway leading to the formation of the β-products is significantly more sensitive to acceptor nucleophilicity, both in terms of electronic and steric effects, than the α-product forming pathways.

### Kinetic isotope effects

To estimate the amount of S_N_1 and S_N_2 character in the reactions of the different nucleophiles, we determined primary ^13^C KIEs for each of the product forming pathways, using partially ^13^C labeled donors for analysis. To this end, we generated a mixture of thioglucoside donors, of which 50% carries a ^13^C at the anomeric position. The benzylidene acetal in these donors was isotopically labeled with 50% ^13^C at the acetal position, to serve as an internal standard. While Crich and co-workers previously reported that non-isotopically labeled substrates can be used at natural abundance, establishing the KIE values for the whole series would be too demanding with regard to the amount of high-field NMR time required.^24^

For the determination of the KIEs, the labelled thioglycosides were fully converted to their triflate counterparts using the general pre-activation procedure for Tf_2_O/Ph_2_SO mediated glycosylations. The acceptor was then added in substoichiometric quantity (0.75 equiv.) to ensure only partial conversion of the triflate to the glycosylated product, to be able to measure the change in the ^12^C/^13^C ratio. The crude product mixture and purified anomers were analyzed using a high field NMR spectrometer (850 MHz) to ensure that the signal to noise ratio was acceptable for integration of the 1D ^13^C NMR spectra. The KIEs of the glycosylation reactions were calculated using eqn (1) where *R*_p_ is the molar activity of the heavy isotope (^13^C) in the glycosylated product, determined by the ratio between the integrals of the anomeric carbon and the benzylidene α-carbon in the product, and *R*_0_ is its molar activity in the reactant, determined by the same signal ratio in the starting thioglycoside. Finally, *F* is the fractional conversion of the starting material into the product, determined by integration of the anomeric proton of the product in the crude reaction mixture against the α-proton of 4,4,5,5-tetramethyl-2-(1-naphthyl)-1,3-dioxolane used as an internal standard. Each reaction was repeated three times and the measured isotope effects were averaged to provide the results tabularized in Table 2. The KIE of the glycosylation reaction of the *C*-nucleophile allyltrimethylsilane was also determined to provide a benchmark for an S_N_1-type glycosylation reaction. Unfortunately, it was not possible to determine accurate KIE values for the ^*t*^BuOH and AdmOH nucleophiles, as the α- and β-products could not be separated and the diagnostic ^13^C resonances overlapped in the NMR spectra, precluding accurate integration.1
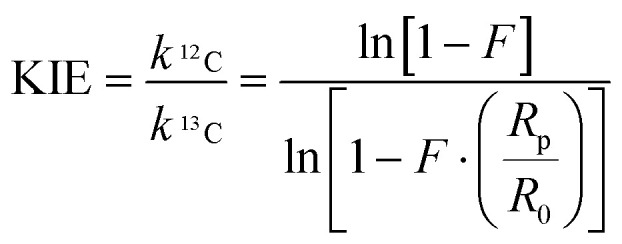
When examining the primary ^13^C KIEs, two distinct trends emerge for the formation of α- and β-glucosides. For the β-glucosides, the KIE values are consistently within the range (>1.030) of bimolecular, S_N_2-like reaction pathways, and the values only marginally diminish in going from EtOH to MFE and DFE. These values support the hypothesis that the β-glucosides are formed through an S_N_2 substitution of α-triflate. The KIE values for the formation of the α-glucosides on the other hand show a clear and gradual development from 1.031 for the most electron rich nucleophile, EtOH, to 1.009 for the most electron poor nucleophile, TFE. The KIE value obtained for the reaction leading to the formation of the α-ethanol product is similar to that for the corresponding β-glucosides (1.031), suggesting that both anomers are formed through a bimolecular S_N_2-like mechanism. The decreasing KIE values for MFE, DFE and TFE indicate that these reactions take place with increasing S_N_1-character, with a clear correlation between the increasing number of electron-withdrawing fluorine atoms and increasing S_N_1-character. The KIE for TFE closely approaches the value obtained for the reaction of the “S_N_1-type nucleophile” allyltrimethylsilane.

**Table 2:** Experimentally determined primary ^13^C kinetic isotope effects (KIEs) for the formation of α- and β-glucopyranosides using 4,6-*O*-benzylidene glucosyl donor, 1, with both sets of model *O*-nucleophiles and allyltrimethylsilane, as a reference for S_N_1-like glycosylation reactions.^a^^,^^b^^,^^c^^,^^d^ Cell shading indicates mechanistic character: increasing green saturation denotes greater S_N_2 contribution, while white corresponds to fully S_N_1 character. Pre-activation-based glycosylation conditions were used: donor 1 (1.0 equiv.), Tf_2_O (1.3 equiv.), Ph_2_SO (1.3 equiv.), TTBP (2.5 equiv.), DCM (0.05 M), −40 °C, then add the nucleophile (0.75 equiv.) and stir for 1 hour at −40 °C. Quantitative ^13^C NMR spectra were recorded on an 850 MHz spectrometer with sufficiently long relaxation delays of at least 10 times the T_1_ of the anomeric and/or benzylidene carbon

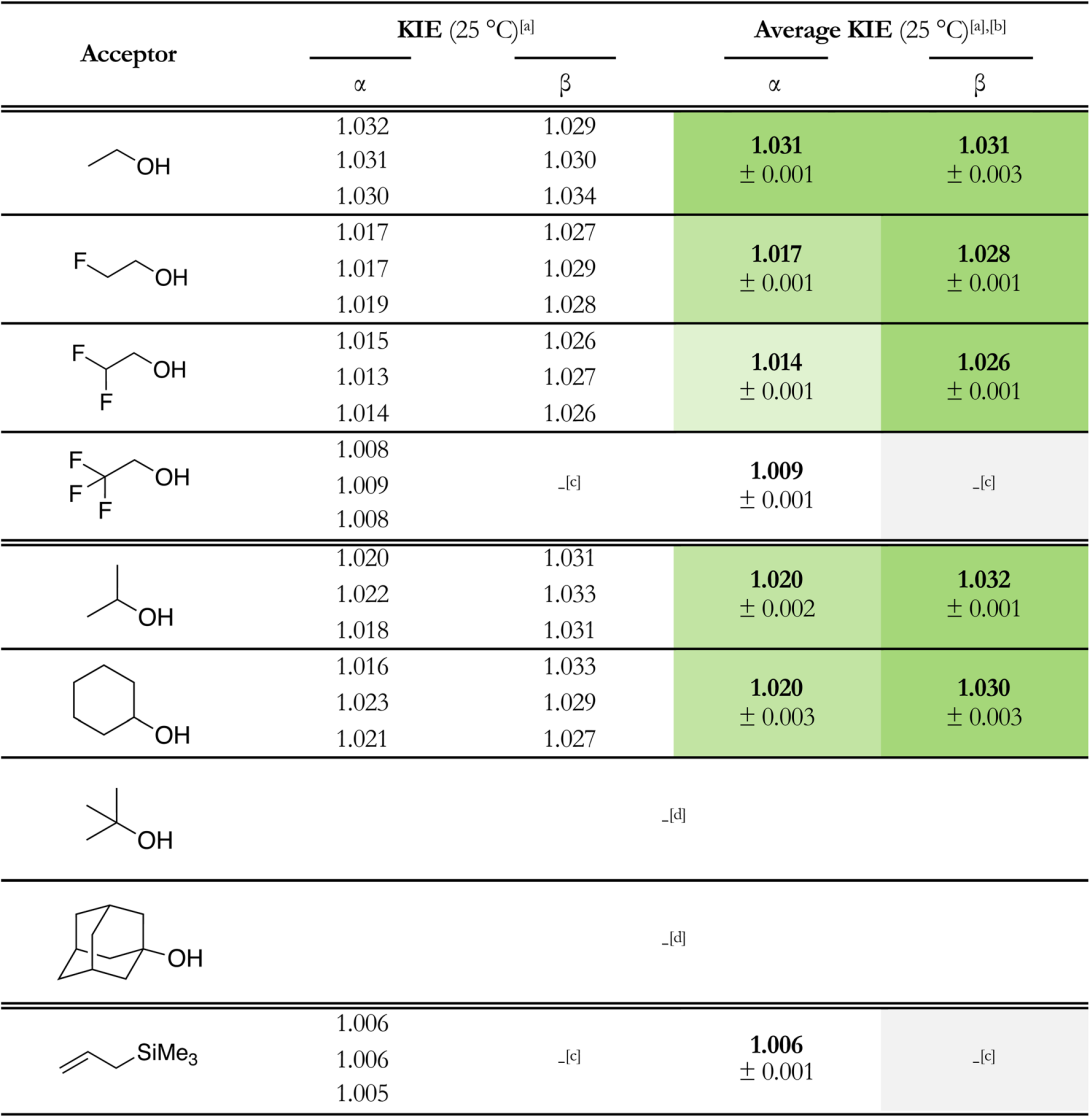

a All KIEs were measured at −40 °C and converted to 25 °C using KIE_25°C_ = exp{[233/298]ln[KIE_−40°C_]}.

b Reported errors are at 1*σ*.

c Too little β-product formed to calculate an accurate KIE.

d Reaction yielded inseparable anomers, so accurate integration, and therefore the calculation of a KIE, was not possible.

The KIE values of the reactions of ^i^PrOH and CyOH providing α-products are lower than those of the corresponding β-product forming reactions, indicating that increasing steric bulk in the acceptors causes a shift to the S_N_1-side of the reaction mechanism continuum. The increased steric bulk of the acceptors can likely be better accommodated in a more exploded transition state.

### Concentration dependence

Because we couldn't establish KIE values for the reactions of the ^*t*^BuOH and AdmOH nucleophiles, we sought to probe the reaction pathways of different acceptors through different kinetic experiments.^29^ To this end we set out to explore how the stereoselectivity of the reaction changes with changing concentration of the acceptors. Assuming that the β-products are formed in an S_N_2-like reaction (as judged from the KIE values reported in Table 2), and that the α-product may be generated in reactions, having both an S_N_2 and an S_N_1 component, we reasoned that formation of the α- and β-products, that are formed through an S_N_2-like reaction would equally depend on changing the acceptor concentration, while the formation of the α-product through an S_N_1-like reaction would be independent of the concentration of the acceptor alcohol.

To probe the influence of acceptor concentration on stereoselectivity, the pre-activation-based Tf_2_O/Ph_2_SO mediated glycosylation conditions were again used to condense glucosyl donor 1, with different quantities of the acceptor: 0.25, 0.50, 1.0, and 2.0 equivalents. The stereoselectivity was determined by integration of quantitative 1D ^13^C-NMR spectra (850 MHz) to ensure accurate integrals, as the ^1^H-NMR spectra were too complex to accurately assign, due to the presence of hydrolysis products in the reactions with substoichiometric amounts of the acceptor.

**Fig. 3:**
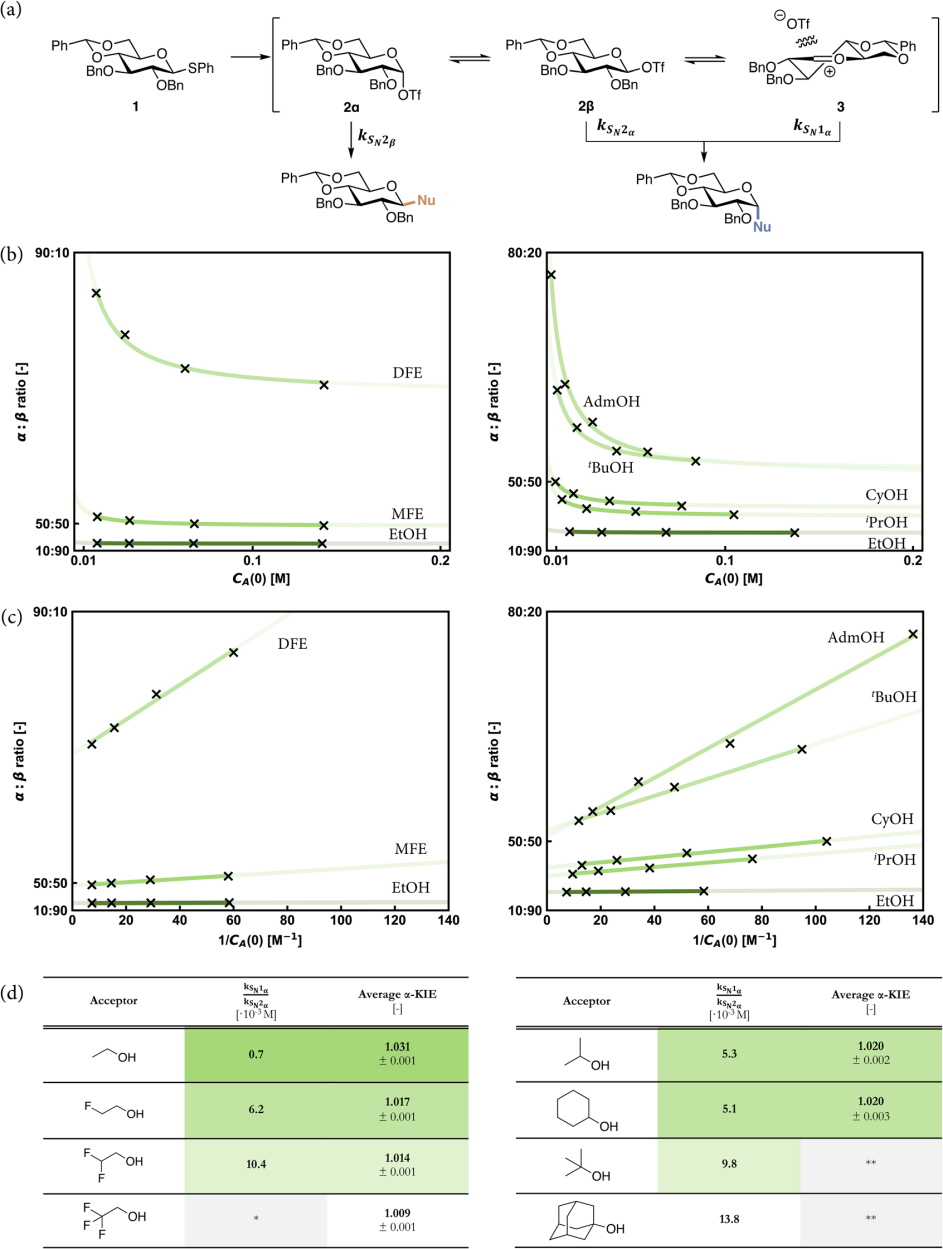
(a) Mechanistic pathways to account for the selectivity in glycosylation reactions of benzylidene glucoside donor 1. (b) Stereoselectivity data for glycosylation reactions using acceptors from both sets of model *O*-nucleophiles with different acceptor concentrations fitted to a reciprocal equation, (c) and a linear fit through the points plotted against reciprocal initial acceptor concentration. (d) A comparison between the S_N_1 *vs.* S_N_2 preference for the formation of the alpha product as computed from the concentration experiments and the computed values for the kinetic isotope effects (KIEs) for these reactions. Reported errors for the KIEs are at 1*σ*. *Concentration dependence cannot be determined for anomerically selective glycosylation reactions, as the determination of an accurate α : β ratio is not possible. **Reaction yielded inseparable anomers, so accurate integration, and therefore the calculation of a KIE, was not possible.

The stereoselectivity of the reactions as a function of acceptor concentration is depicted in Fig. 3b, with the left panel depicting the series of nucleophiles that differ in electronic properties and the right panel showing the series of acceptors with changing steric demands. From the graphs of the (partially) fluorinated acceptors, it becomes apparent that the α/β-product ratio for the reactive acceptors (EtOH and MFE) does not change with changing acceptor concentration, while the ratio of the α/β-DFE-products strongly depends on the concentration of the nucleophile. Of note, because of the high and constant α-selectivity (>98 : 2) no meaningful graph for TFE can be plotted. The series of acceptors, differing in steric demand, provides a similar picture: the α/β-product ratio for the glucosides formed from the small, reactive nucleophiles does not change with acceptor concentration, while the stereoselectivity of the reactions of the sterically more demanding and less nucleophilic acceptors, more strongly changes with increasing steric demand.

To interpret the data more quantitatively, the system of reactions, shown in Fig. 3a, can be described using a set of rate equations:2
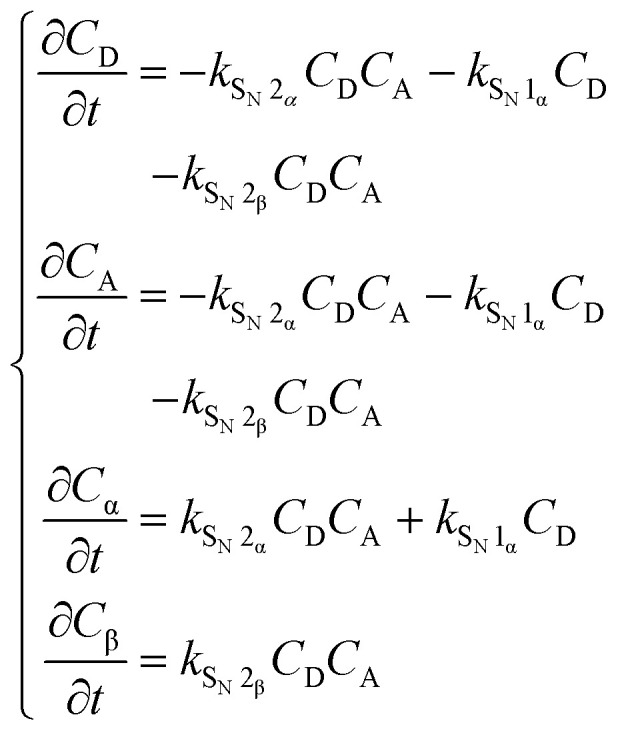
In these equations, *C*_A_, *C*_α_ and *C*_β_ are the concentrations of the acceptor, and the α- and β-products respectively. Assuming Curtin–Hammett kinetics, in which the triflate interconversion is much faster than the substitution of the triflates, the anomeric α- and β-triflates can be grouped together as one donor species, D, with its concentration expressed as *C*_D_. Assuming that the α-forming pathway can be divided into an S_N_1-like and an S_N_2-like component, while the formation of the β-products takes place solely through an S_N_2-like pathway, the set of equations can be simplified to the following expression for the product ratio at complete conversion (*t* → ∞); see the SI for the derivation of this approximation using eqn (S1)–(S15).3
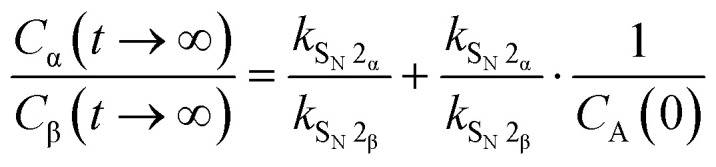
Thus, when the anomeric product ratio is plotted as a function of the reciprocal acceptor concentration, 1/*C*_A_(0) (Fig. 3c), the slope of the line represents the ratio of the rate constants for the S_N_1-pathway leading to the α-product and the S_N_2-pathway leading to the β-product (*k*_S_N_1_α__/*k*_S_N_2_β__), and the intercept of the *y*-axis provides the ratio of the S_N_2-rate constants leading to the α- and β-products (*k*_S_N_2_α__/*k*_S_N_2_β__). Dividing the slope of the lines by the y-axis intercept then gives the ratio of the rate constants for the S_N_1 and S_N_2-reactions leading to the α-product (*k*_S_N_1_α__/*k*_S_N_2_α__). This can be taken as a measure for the amount of S_N_1-character in the formation of the α-products and thus serves as an alternative for the establishment of KIE values. The values so obtained, together with the corresponding α-KIE values are summarized in Fig. 3d. Combined, these values provide a quantitative picture of how the S_N_1-character in α-product forming reactions increases with decreasing electron density and increasing steric demand for the acceptor alcohols. While we couldn't obtain KIE values for the reactions of *t*BuOH and AdmOH, the *k*_S_N_1_α__/*k*_S_N_2_α__ values for these reactions (9.8 and 13.8 respectively) show that these reactions proceed with a significant amount of S_N_1-character. Similar to the gradual development of the KIE values with increasing fluorine atoms, the *k*_S_N_1_α__/*k*_S_N_2_α__ values also gradually develop as steric demand increases. Both increasing electron-withdrawing character of the substituents and steric requirements shift the reaction mechanism continuum towards the S_N_1 side.

## Conclusions

In this study, we have systematically investigated how the mechanistic pathways of glycosylation reactions of the 4,6-*O*-benzylidene protected glucosyl donor system change as a function of structural changes in the acceptor alcohols. While it has been broadly accepted that glycosylations take place along a continuum of S_N_1-like and S_N_2-like pathways, it has remained exceedingly difficult to predict where on the continuum a given reaction will take place and how much the mechanisms change with changes in the structure of the donor and acceptor reaction partner. The 4,6-*O*-benzylidene protected glucosyl donor stands out as an exceptional model glycosylation system as the stereoselectivity of its glycosylation reactions is directly related to the reactivity of the acceptor nucleophiles. Here we have used systematic sets of acceptors that gradually change in electron density and steric surrounding of the alcohol nucleophile to map how they affect the possible reaction pathways. The combination of acceptor competition experiments, establishment of KIE values and kinetic experiments, that have revealed how stereoselectivity can depend on acceptor concentration, have clearly illustrated how the glycosylation pathways shift on the reaction mechanism continuum from an S_N_2-like pathway towards reactions proceeding with a more exploded transition state having significantly more S_N_1-character for acceptors that are less electron rich, or that have more stringent steric demands. At the same time, it has been illustrated that the substitution of α- and β-anomeric triflates with the same nucleophile does not take place with an equal amount of S_N_2/S_N_1-character. The acceptor competition experiments have revealed that acceptors that are generally considered to be “less nucleophilic”, because of the presence of more electron withdrawing substituents, can in fact react faster than their more electron rich counterparts.

Other donor systems will have to be exposed to the same systematic set of acceptors in the future to shed more light on how glycosylation mechanisms change with structural changes in the donor and how these mechanisms are impacted by the nature of the acceptor glycoside. This will provide guidelines on how to control the balance between S_N_2- and S_N_1-type substitutions, enabling rationally optimized and ever more effective glycosylation chemistry.

## Author contributions

JDCC and GAM: conceptualisation; DH, KNAV, DM, WAR, CT: investigation; JDCC and GAM: supervision; DH and JDCC: writing – original draft; DH, KNAV, DM, WAR, CT, GAM, JDCC: writing – review & editing.

## Conflicts of interest

There are no conflicts to declare.

## Supplementary Material

SC-017-D5SC07201H-s001

## Data Availability

The data supporting this article have been included as part of the supplementary information (SI). Supplementary information is available. See DOI: https://doi.org/10.1039/d5sc07201h.
