# Supplementary material for: How electronic and steric effects in acceptor alcohols shape SN1- and SN2-type glycosylation reactions
Source: Chem Sci. 2025 Nov 17;17(3):1761–70. doi: 10.1039/d5sc07201h (PMC12666880; doi:10.1039/d5sc07201h)
Supplement: SC-017-D5SC07201H-s001 [file SC-017-D5SC07201H-s001.pdf]

Supplementary Information (SI) for Chemical Science.
This journal is © The Royal Society of Chemistry 2025

Supplementary Information

"How Electronic and Steric Effects in Acceptor Alcohols Shape S_N1- and S_N2-Type Glycosylation Reactions"

Daan Hoogers[1,2], Koen N.A. van de Vrande[1,2], Dennis van der Meij[1], Wouter A. Remmerswaal[1], Coralie Tugny[1], Gijsbert A. van der Marel[1], Jeroen D. C. Codée[1,3]

[1]*Leiden University, Leiden Institute of Chemistry, Einsteinweg 55, 2333 CC Leiden, The Netherlands.*
[2]*These authors contributed equally.*
[3]*Corresponding author, email address: jcodee@chem.leidenuniv.nl.*

Concentration Dependence - Reaction Kinetics Derivation

As outlined in the main text, when assuming Curtin Hammett type kinetics the reaction system can be defined by a system of four differential equations describing the concentrations of the donor, C_D, the acceptor, C_A, and both the alpha and beta products, C_α and C_β:

$$\begin{cases} \frac{\partial C_D}{\partial t} = -k_{S_N 2_\alpha} C_D C_A - k_{S_N 1_\alpha} C_D - k_{S_N 2_\beta} C_D C_A \\ \frac{\partial C_A}{\partial t} = -k_{S_N 2_\alpha} C_D C_A - k_{S_N 1_\alpha} C_D - k_{S_N 2_\beta} C_D C_A \\ \frac{\partial C_\alpha}{\partial t} = k_{S_N 2_\alpha} C_D C_A + k_{S_N 1_\alpha} C_D \\ \frac{\partial \text{[illegible]}_\beta}{\partial t} = k_{S_N 2_\beta} C_D C_A \end{cases} \tag{S1}$$

For this system of equations, we use the initial conditions where there is only donor present. For simplicity purposes, the initial concentration was chosen to be 1.

$$\begin{cases} C_{D,i} = 1 \\ C_{\alpha,i} = 0 \\ C_{\beta,i} = 0 \end{cases} \tag{S2}$$

Also, the initial acceptor concentration, $C_{A,i}$, was kept variable, as we were interested in its effects on the product ratio at full conversion ($t \to \infty$). However, we do know that the change in acceptor is the same as that of the donor, as can be seen in **Equation S1**. This leads to the following simplified expression for C_A:

$$C_A(t) = C_D(t) + \Delta C_0 \tag{S3}$$

Where ΔC_0 is the initial offset in concentration between the acceptor and the donor (e.g., when 2 equivalents of acceptor are used, ΔC_0 will be 1). Given this relationship we can substitute **Equation S3** into the differential equations for C_α.

$$\frac{\partial C_\alpha}{\partial t} = k_{S_N 2_\alpha} C_D (C_D + \Delta C_0) + k_{S_N 1_\alpha} C_D \tag{S4}$$

which, upon expanding, becomes,

$$\frac{\partial C_\alpha}{\partial t} = k_{S_N 2_\alpha} C_D^2 + k_{S_N 2_\alpha} C_D \Delta C_0 + k_{S_N 1_\alpha} C_D \tag{S5}$$

When we do the same for the differential equation describing C_β we get:

$$\frac{\partial C_\beta}{\partial t} = k_{S_N 2_\beta} C_D^2 + k_{S_N 2_\beta} C_D \Delta C_0 \tag{S6}$$

Now, to find $C_\alpha(t \to \infty)$ and $C_\beta(t \to \infty)$, we integrate both equations over time.

$$C_\alpha(t \to \infty) = \int_0^\infty (k_{S_N2_\alpha} C_D^2 + k_{S_N2_\alpha} C_D \Delta C_0 + k_{S_N1_\alpha} C_D) dt \tag{S7}$$

And,

$$C_\beta(t \to \infty) = \int_0^\infty (k_{S_N2_\beta} C_D^2 + k_{S_N2_\beta} C_D \Delta C_0) dt \tag{S8}$$

We can then factor out these integrals and simplify the expressions to:

$$C_\alpha(t \to \infty) = k_{S_N2_\alpha} \int_0^\infty C_D(t)^2 dt + k_{S_N2_\alpha} \Delta C_0 \int_0^\infty C_D(t) dt + k_{S_N1_\alpha} \int_0^\infty C_D(t) dt \tag{S9}$$

And,

$$C_\beta(t \to \infty) = k_{S_N2_\beta} \int_0^\infty C_D(t)^2 dt - k_{S_N2_\beta} \Delta C_0 \int_0^\infty C_D(t) dt \tag{S10}$$

Now the ratio, $\frac{C_\alpha(t \to \infty)}{C_\beta(t \to \infty)}$, is given by:

$$\frac{C_\alpha(t \to \infty)}{C_\beta(t \to \infty)} = \frac{k_{S_N2_\alpha} \int_0^\infty C_D(t)^2 dt + k_{S_N2_\alpha} \Delta C_0 \int_0^\infty C_D(t) dt + k_{S_N1_\alpha} \int_0^\infty C_D(t) dt}{k_{S_N2_\beta} \int_0^\infty C_D(t)^2 dt - k_{S_N2_\beta} \Delta C_0 \int_0^\infty C_D(t) dt} \tag{S11}$$

Which simplifies to,

$$\frac{C_\alpha(t \to \infty)}{C_\beta(t \to \infty)} = \frac{k_{S_N2_\alpha}}{k_{S_N2_\beta}} + \frac{k_{S_N1_\alpha}}{k_{S_N2_\beta} \frac{\int_0^\infty C_D(t)^2 dt}{\int_0^\infty C_D(t) dt} - k_{S_N2_\beta} \Delta C_0} \tag{S12}$$

For the functions *$C_A(t)$* and *$C_D(t)$*, we assume they exhibit exponential-like decay behavior as t increases. Specifically, we assume these equations follow decay behavior of the form:

$$C_D(t) \propto C_D(0)\, e^{-\lambda t} \tag{S13}$$

And thus,

$$\frac{\int_0^\infty C_D(t)^2 dt}{\int_0^\infty C_D(t) dt} \propto \frac{C_D(0)^2}{C_D(0)} = C_D(0) = C_A(0) + \Delta C_0 \tag{S14}$$

Substituting **Equation S14** into **Equation S12**, and contracting some constants together, leaves us with the qualitative description of the acceptor concentration dependence of glycosylation stereoselectivity, which was also empirically confirmed by numerically simulating this set of differential equations:

$$\frac{C_\alpha(t \to \infty)}{C_\beta(t \to \infty)} = \frac{k_{S_N2_\alpha}}{k_{S_N2_\beta}} + \frac{k_{S_N1_\alpha}}{k_{S_N2_\beta}} \cdot \frac{1}{C_A(0)} \tag{S15}$$

General Procedures

General Methods. All chemicals (Acros, Fluka, Merck, and Sigma-Aldrich) were used as received unless stated otherwise. Dichloromethane was stored over activated 4 Å molecular sieves (beads, 8-12 mesh, Sigma-Aldrich). Before use traces of water present in the donor, diphenyl sulfoxide (Ph_2SO) and tri-*tert*-butylpyrimidine (TTBP) were removed by co-evaporation with dry toluene. The acceptors were stored in stock solutions (DCM, 0.5 M) over activated 3 Å molecular sieves (rods, size 1/16 in., Sigma-Aldrich). Trifluoromethanesulfonic anhydride (Tf_2O) was distilled over P_2O_5 and stored at −20 °C under a nitrogen atmosphere. Overnight temperature control was achieved by an FT902 Immersion Cooler (Julabo). Column chromatography was performed on silica gel 60 Å (0.04 – 0.063 mm, Screening Devices B.V.). TLC-analysis was conducted on TLC Silica gel 60 (Kieselgel 60 F254, Merck) with UV detection by (254 nm) and by spraying with 20% sulfuric acid in ethanol followed by charring at ± 150 °C or by spraying with a solution of $(NH_4)_6Mo_7O_{24} \cdot H_2O$ (25 g/l) and $(NH_4)_4Ce(SO_4)_4 \cdot 2H_2O$ (10 g/l) in 10% aq. sulfuric acid followed by charring at ± 250 °C. High-resolution mass spectra were recorded on a Thermo Finnigan LTQ Orbitrap mass spectrometer equipped with an electrospray ion source in positive mode (source voltage 3.5 kV, sheath gas flow 10, capillary temperature 275 °C) with resolution R=60.000 at m/z = 400 (mass range = 150 – 4000). ^1H and ^{13}C NMR spectra were recorded on a Bruker AV-300 NMR instrument (300 and 75 MHz respectively), a Bruker AV-400 NMR instrument (400 and 101 MHz respectively), a Bruker AV-500 NMR instrument (500 and 126 MHz respectively), a Bruker AV-600 NMR instrument (600 and 151 MHz respectively), or a Bruker AV-850 NMR instrument (850 and 214 MHz respectively). For samples measured in $CDCl_3$ chemical shifts (δ) are given in ppm relative to tetramethylsilane (TMS) as an internal standard or the residual signal of the deuterated solvent. Coupling constants (*J*) are given in Hz. To get better resolution of signals with small coupling constants or overlapping signals a gaussian window function (LB ± −1 and GB ± 0.5) was used on the ^1H NMR spectrum. All given ^{13}C-APT spectra are proton decoupled. NMR peak assignment was made using HH-COSY, and HSQC. If necessary additional HH-NOESY, HMBC and HMBC-GATED experiments were used to elucidate the structure further. The anomeric product ratios were based on the integration of ^1H NMR spectra of the purified products and was checked with the spectra of the crude reaction mixture.

General procedure for Tf_2O/Ph_2SO mediated glycosylations. Donor (0.1 mmol), Ph_2SO (26 mg, 0.13 mmol, 1.3 eq.) and TTBP (62 mg, 0.25 mmol, 2.5 eq.) were co-evaporated twice with dry toluene (4 Å molecular sieves) and dissolved in anhydrous DCM (2.0 mL, 0.05 M donor). Activated 3 Å molecular sieves (rods, size 1/16 in.) were added and the reaction mixture was stirred for 15 min at room temperature. The solution was cooled to −80 °C and Tf_2O (22 μL, 0.13 mmol, 1.3 eq.) was slowly added. The reaction mixture was allowed to warm to −60 °C in approximately 45 min,

followed by recooling to −80 °C in approximately 15 min, whereafter acceptor (0.2 mmol, 2.0 eq.) in DCM (0.4 mL, 0.5 M) was added slowly. The reaction mixture was allowed to warm to −40 °C in approximately 45 min and stirred for an additional 16 h. The reaction was quenched with sat. aq. $NaHCO_3$ solution (1.0 mL) at −40 °C and diluted with DCM. The solution was transferred to a separatory funnel and water was added, the layers were separated, and the aqueous phase was extracted twice with DCM. The combined organic layers were dried over $MgSO_4$, filtered, and concentrated under reduced pressure. Flash column chromatography yielded the glycosylation product as a mixture of anomers.

General procedure for kinetic isotope effect (KIE) experiments. For the acquisition of the quantitative ^{13}C-NMR spectra, inverse gated bi-level decoupling via Waltz-65 sequence was applied with carrier frequency between the neighboring protons, to avoid creation of heteronuclear Overhauser effect and to eliminate decoupling sidebands. The inversion-recovery technique was used for the estimation of the T_1 relaxation times, which were found 1.17 s and 1.15 s for the benzylidene and anomeric carbon respectively. Relaxation delays (15.0 s) were chosen accordingly. ^{13}C NMR spectra were typically recorded with 512 scans, which resulted in a signal-to-noise ratio between 2000 and 5000. All spectra were baseline corrected using a polynomial line fit and a 2.0 Hz exponential apodization function was applied prior to integration. Integrals were recorded by fitting a generalized Lorentzian curve to the signals of interest using the global spectral deconvolution (GSD) module from Mestrelabs until good overlap of lineshapes between the experimental and simulated spectra was reached.

Kinetic isotope effects (KIEs) of glycosylation reactions were calculated from equation (S16) where Rp is the molar activity of the minor isotope (^{13}C) in the glycosylated product, determined by integration of anomeric carbon in the product, and R_0 is the molar activity of the minor isotope (^{13}C) in the reactant, determined by integration of the anomeric carbon in starting thioglycoside. Assuming that the benzylidene carbon is far enough from the reaction center to be used as an internal standard, the anomeric carbon was integrated against the benzylidene carbon to get R_p and R_0. For any given reaction type, at least four experiments were performed and the isotope effects were averaged to give an accurate result.

$$\mathrm{KIE} = \frac{\ln[1 - F]}{\ln\left[1 - F\left(\frac{\mathrm{R_p}}{\mathrm{R_0}}\right)\right]} \qquad \text{(S16)}$$

In equation (S16), F is the fractional conversion of the starting material into the product, determined by integration of the anomeric proton of that product in the crude against the α-proton of 4,4,5,5-tetramethyl-2-(1-naphthyl)-1,3-dioxolane used as an internal standard. As the true initial reactant is an α-triflate too instable for quantitative ^{13}C NMR experiments, an excess of triflic anhydride (1.3 eq.) was used to ensure complete conversion of the starting thioglycoside into a covalent triflate species. As the ^{13}C content for the anomeric carbon is preserved in this step, the quantitative ^{13}C NMR experiments were carried out on the starting thioglycoside. In order to observe the KIE a substoichiometric amount (0.75 eq.) of acceptor was then added.

For the glycosylation reactions, 50% isotopically labelled donor (54 mg, 0.1 mmol), Ph_2SO (26 mg, 0.13 mmol, 1.3 eq.), 4,4,5,5-tetramethyl-2-(1-naphthyl)-1,3-dioxolane (13 mg, 0.05 mmol,

0.5 eq.) and TTBP (62 mg, 0.25 mmol, 2.5 eq.) were pre-mixed and transferred to an NMR tube. From this mixture, quantitative ^{1}H NMR (to determine *F*) and ^{13}C NMR (to determine R_0 and R_p) spectra were recorded. Thereafter, the mixture was co-evaporated twice with dry toluene (4 Å molecular sieves) and dissolved in anhydrous DCM (2.0 mL, 0.05 M donor). Activated 3 Å molecular sieves (rods, size 1/16 in.) were added and the reaction mixture stirred for 15 min at room temperature. The solution was cooled to −80 °C and Tf_2O (22 μL, 0.13 mmol, 1.3 eq.) was slowly added. The reaction mixture was allowed to warm to −40 °C in approximately 45 min, whereafter acceptor (0.075 mmol, 0.75 eq.) in DCM (0.15 mL, 0.5 M) was added in one portion. The reaction mixture was allowed to stir for 1 hour, whereafter the reaction was quenched by the addition of sat. aq. $NaHCO_3$ solution (1.0 mL) at −40 °C and diluted with DCM. The solution was transferred to a separatory funnel and water was added, the layers were separated, and the aqueous phase was extracted twice with DCM. The combined organic layers were dried over $MgSO_4$, filtered, and concentrated under reduced pressure. From the crude reaction mixture, a quantitative ^{1}H NMR spectrum (to determine *F*) was recorded. Flash column chromatography afforded the glycosylation products as separated anomers, of which both of them were subjected to quantitative ^{13}C NMR experiments (to determine R_0 and R_p). Experiments were performed at least four times and their KIEs were averaged to ensure accurate results.

General procedure for acceptor concentration experiments. The same quantitative ^{13}C-NMR procedure as described above for the KIE measurements was used to evaluate the integrals of the anomeric protons of the glycosylated products in the crude reaction mixture. For the glycosylation reactions, all reagents were scaled up by a factor of two to be able to be able to detect the small amount of product formed when increasingly substoichiometric amounts of acceptor alcohol were used. Donor (0.2 mmol), Ph_2SO (52 mg, 0.26 mmol, 1.3 eq.) and TTBP (124 mg, 0.5 mmol, 2.5 eq.) were co-evaporated twice with dry toluene (4 Å molecular sieves) and dissolved in anhydrous DCM (4.0 mL, 0.05 M donor). Activated 3 Å molecular sieves (rods, size 1/16 in.) were added and the reaction mixture was stirred for 15 min at room temperature. The solution was cooled to −80 °C and Tf_2O (44 μL, 0.26 mmol, 1.3 eq.) was slowly added. The reaction mixture was allowed to warm to −60 °C in approximately 45 min, followed by recooling to −80 °C in approximately 15 min, whereafter a variable amount of acceptor in DCM (0.4 mL) was added slowly. Acceptor amounts were 4.0, 8.0, 16.0 and 32.0 μL of each for ease of measurements. These were later converted to molar amounts for data analysis. 1-Adamantanol, being a solid, was weighed and added in amounts of 4.0, 8.0, 16.0 and 32.0 mg in the same amount of DCM. The reaction mixture was allowed to warm to −40 °C in approximately 45 min and stirred for an additional 16 h. The reaction was quenched with sat. aq. $NaHCO_3$ solution (1.0 mL) at −40 °C and diluted with DCM. The solution was transferred to a separatory funnel and water was added, the layers were separated, and the aqueous phase was extracted twice with DCM. The combined organic layers were dried over $MgSO_4$, filtered, and concentrated under reduced pressure. The crude reaction mixtures were subjected to quantitative ^{13}C-NMR and the anomeric ratios were plotted against (reciprocal) acceptor concentration.

Experimental Procedures

Phenyl 2,3,4,6-tetra-*O*-acetyl-1-thio-β-D-glucopyranoside (S1)

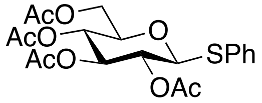

To Ac_2O (200.0 mL, 2.1 mol, 7.6 eq.) and 5 drops of 70% aq. $HClO_4$, D-glucose (50.0 g, 278.0 mmol) was added portion wise at 0 °C. After 20 minutes, HBr (33 wt% in AcOH, 228 mL, 1.4 mol, 5.0 eq.) was added and the reaction was stirred. After 16 h, the reaction mixture was diluted with water at 0 °C and extracted twice with DCM. The combined organic layers were washed with sat. aq. $NaHCO_3$ solution, brine, dried over $MgSO_4$, filtered and concentrated under reduced pressure. To a solution of the crude bromide in DMF (250 mL, 1.0 M), PhSH (32.0 mL, 314.0 mmol, 1.1 eq.) was added. The reaction mixture was cooled to 0 °C, and NaH (60 wt% dispersion in mineral oil, 14.5 g, 361.0 mmol, 1.3 eq.) was added portion wise. The mixture was stirred for 2 hours or until TLC (60:40, *n*-Pentane:EtOAc, v:v) indicated complete conversion of the starting material. Thereafter, the reaction was quenched by the addition of water at 0 °C. The resulting black suspension was extracted twice with DCM. The combined organic layers were washed with sat. aq. $NaHCO_3$, 1.0 M aq. NaOH solution, sat. aq. $Na_2S_2O_3$ solution, brine, dried over $MgSO_4$ and concentrated under reduced pressure. Flash column chromatography (90:10 to 70:30, *n*-Pentane:EtOAc, v:v) and subsequent recrystallization from boiling EtOH afforded the title compound (63.7 g, 144.6 mmol, 52%) as a white solid. Spectroscopic data were in accord with those previously recorded. TLC: R_f 0.49, (70:30, *n*-Pentane:EtOAc, v:v); ^{1}H NMR ($CDCl_3$, 400 MHz, HH-COSY, HSQC): δ 7.51 – 7.49 (m, 2H, CH_{arom}), 7.35 – 7.28 (m, 3H, CH_{arom}), 5.23 (t, *J* = 9.4 Hz, 1H, H-3), 5.05 (t, *J* = 9.8 Hz, 1H, H-4), 4.98 (t, *J* = 9.6 Hz, 1H, H-2), 4.71 (d, *J* = 10.1 Hz, 1H, H-1), 4.23 (dd, *J* = 12.3, 5.1 Hz, 1H, H-6), 4.18 (dd, *J* = 12.3, 2.8 Hz, 1H, H-6), 3.73 (ddd, *J* = 10.1, 5.0, 2.6 Hz, 1H, H-5), 2.09 (s, 3H, CH_3 Ac), 2.09 (s, 3H, CH_3 Ac), 2.03 (s, 3H, CH_3 Ac), 2.00 (s, 3H, CH_3 Ac); ^{13}C-APT NMR (101 MHz, $CDCl_3$, HSQC): δ 170.7, 170.3, 169.5, 169.4 (C=O Ac), 133.2 (CH_{arom}), 131.7(C_q), 129.1, 128.5 (CH_{arom}), 85.8 (C-1), 75.9 (C-5), 74.1 (C-3), 70.0 (C-2), 68.3 (C-4), 62.2 (C-6), 20.9, 20.9, 20.7, 20.7 (CH_3 Ac); HRMS: $[M+Na]^+$ calcd. for $C_{20}H_{24}O_9SNa^+$ 463.1033, found 463.1032.

Phenyl 1-thio-β-D-glucopyranoside (S2)

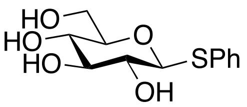

Compound **S1** (46.8 g, 106.4 mmol) was co-evaporated with anhydrous toluene and dissolved in MeOH (400 mL, 0.25 M). To the solution, NaOMe (0.54 g, 10.6 mmol, 0.1 eq.) was added and the reaction mixture was stirred at r.t. for 16 hours or until TLC (90:10, DCM:MeOH, v:v) indicated complete conversion of the starting material. The reaction was quenched by the addition of Amberlite IR120 H^+ until a pH of 4 was reached. The mixture was filtered, and the filtrate was concentrated under reduced pressure. Flash column chromatography (99:1 to 80:20, DCM:MeOH, v:v) afforded the title compound (26.0 g, 103.2 mmol, 97%) as a white solid. Spectroscopic data were in accord with those previously recorded. TLC: R_f 0.49, (80:20, DCM:MeOH, v:v); ^{1}H NMR (MeOD, 400 MHz, HH-COSY, HSQC): δ 7.61 – 7.54 (m, 2H, CH_{arom}), 7.33 – 7.24 (m, 3H, CH_{arom}), 4.60 (d, *J* = 9.8 Hz, 1H, H-1), 3.87 (dd, *J* = 12.1, 1.8 Hz, 1H, H-6), 3.67 (dd, *J* = 12.2, 5.2 Hz, 1H, H-6), 3.39 (t, *J* = 8.5 Hz, 1H, H-3), 3.35 – 3.26 (m, 2H, H-4, H-5), 3.22 (dd, *J* = 9.8, 8.6 Hz, 1H, H-2); ^{13}C-APT NMR (101 MHz, $CDCl_3$, HSQC): δ 135.3 (C_q), 132.7, 129.9, 128.3 (CH_{arom}), 89.4 (C-1), 82.0 (C-4), 79.7

(C-3), 73.7 (C-2), 71.3 (C-5), 62.8 (C-6); HRMS: $[M+Na]^+$ calcd. for $C_{12}H_{16}O_5SNa^+$ 295.0616, found 295.0617.

Phenyl 4,6-*O*-benzylidene-1-thio-β-ᴅ-glucopyranoside (S3)

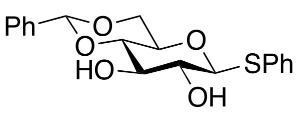

Compound **S2** (26.0 g, 103.2 mmol) was coevaporated with anhydrous DMF and consequently dissolved in dry ACN (400 mL, 0.25 M). Benzaldehyde dimethyl acetal (22.7 g, 149 mmol, 1.4 eq.) and *p*-TsOH (2.0 g, 10.6 mmol, 0.1 eq.) were added and the reaction was stirred and heated to 60 °C under reduces pressure (250-300 mbar) until TLC (95:5, DCM:MeOH, v:v) indicated complete conversion of the starting material. The partially concentrated reaction mixture was resuspended in ACN and subsequently quenched with Et_3N (40 mL). The mixture was then concentrated under reduced pressure and recrystallized from boiling EtOH, affording the title compound (33.1 g, 91.7 mmol, 89%) as a white solid. Spectroscopic data were in accord with those previously recorded. TLC: R_f 0.72, (95:5, DCM:MeOH, v:v); ^{1}H NMR ($CDCl_3$, 400 MHz, HH-COSY, HSQC): δ 7.60 – 7.54 (m, 2H, CH_{arom}), 7.54 – 7.45 (m, 2H, CH_{arom}), 7.44 – 7.33 (m, 6H, CH_{arom}), 5.55 (s, 1H, C*H*Ph), 4.66 (d, *J* = 9.7 Hz, 1H, H-1), 4.41 (dd, *J* = 10.5, 4.4 Hz, 1H, H-6), 3.93 – 3.69 (m, 2H, H-3, H-6), 3.63 – 3.42 (m, 3H, H-2, H-4, H-5), 2.92 (bd, *J* = 2.3 Hz, 1H, O*H*), 2.77 (bd, *J* = 2.5 Hz, 1H, O*H*); ^{13}C-APT NMR (101 MHz, $CDCl_3$, HSQC): δ 136.9 (C_q), 133.1 (CH_{arom}), 130.6 (C_q), 129.2, 128.5, 128.4, 126.3 (CH_{arom}), 102.0 (CHPh), 88.6 (C-1), 80.2 (C-4), 74.6 (C-3), 72.6 (C-2), 70.6 (C-5), 68.6 (C-6); HRMS: $[M+Na]^+$ calcd for $C_{19}H_{20}O_5SNa^+$ 383.0924, found 383.0923.

Phenyl 2,3-di-*O*-benzyl-4,6-*O*-benzylidene-1-thio-β-ᴅ-glucopyranoside (1)

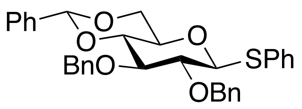

Compound S2 (33.1 g, 91.7 mmol) was co-evaporated with anhydrous toluene, dissolved in dry DMF (1.0 L, 0.1 M) and cooled to 0 °C. To the solution, NaH (60 wt% dispersion in mineral oil, 3.0 eq.) and BnBr (2.4 eq.) were added, and the reaction mixture was allowed to warm up to r.t. After stirring for 5 hours or until TLC (70:30, *n*-Pentane:Et_2O, v:v) indicated complete conversion of the starting material. The reaction was quenched by the addition of H_2O at 0 °C. The aqueous phase was extracted thrice with Et_2O, and the combined organic layers were washed with sat. aq. $NaHCO_3$ solution, brine, dried over $MgSO_4$, filtered and concentrated under reduced pressure. Recrystallization from EtOAc:*n*-Pentane afforded the title compound (44.1 g, 81.6 mmol, 89%) as a fluffy white solid. Spectroscopic data were in accord with those previously recorded. TLC: R_f 0.69, (80:20, *n*-Pentane:EtOAc, v:v); ^{1}H NMR (500 MHz, $CDCl_3$, HH-COSY, HSQC): δ 7.58 – 7.23 (m, 20H, CH_{arom}), 5.59 (s, 1H, C*H*Ph), 4.94 (d, *J* = 11.2 Hz, 1H, C*H*H Bn), 4.86 (d, *J* = 10.3 Hz, 1H, C*H*H Bn), 4.82 (d, J = 10.3 Hz, 1H, CH*H* Bn), 4.78 (d, *J* = 11.1 Hz, 1H, CH*H* Bn), 4.76 (d, *J* = 9.8 Hz, 1H, H-1), 4.39 (dd, *J* = 10.5, 5.0 Hz, 1H, H-6), 3.84 (dd, *J* = 9.4, 8.3 Hz, 1H, H-3), 3.80 (t, *J* = 10.3 Hz, 1H, H-6), 3.71 (t, *J* = 9.4 Hz, 1H, H-4), 3.51 (dd, *J* = 9.8, 8.4 Hz, 1H, H-2), 3.47 (dt, *J* = 9.8, 4.9 Hz, 1H, H-5); ^{13}C-APT NMR (126 MHz, $CDCl_3$, HSQC): δ 138.4, 138.2, 137.4, 133.2 (C_q), 132.5, 129.2, 129.1, 128.5, 128.5, 128.4, 128.4, 128.3, 128.0, 128.0, 127.9, 126.1 (CH_{arom}), 101.3 (CHPh), 88.4 (C-1), 83.2 (C-3), 81.6 (C-4), 80.6 (C-2), 76.0 (CH_2 Bn), 75.5 (CH_2 Bn), 70.4 (C-5), 68.9 (C-6); ^{13}C-GATED NMR (126 MHz, $CDCl_3$): δ 88.4 ($J_{C1,H1}$ = 156.9 Hz, C-1 β); HRMS: $[M+H]^+$ calcd. for $C_{33}H_{33}O_5S^+$ 541.2043, found 541.2039.

Phenyl 2,3,4,6-tetra-*O*-acetyl-1-thio-β-D-glucopyranoside[1-^{13}C] (S4)

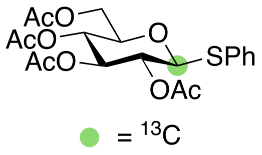

To Ac_2O (40.0 mL, 0.4 mol, 7.6 eq.) and 2 drops of 70% aq. $HClO_4$, D-glucose[1-^{13}C] (10.0 g, 55.6 mmol) was added portion wise at 0 °C. After 20 minutes, HBr (33 wt% in AcOH, 45.6 mL, 0.3 mol, 5.0 eq.) was added and the reaction was stirred. After 16 h, the reaction mixture was diluted with water at 0 °C and extracted twice with DCM. The combined organic layers were washed with sat. aq. $NaHCO_3$ solution, brine, dried over $MgSO_4$, filtered and concentrated under reduced pressure. To a solution of the crude bromide in DMF (100 mL, 0.5 M), PhSH (6.4 mL, 61.2 mmol, 1.1 eq.) was added. The reaction mixture was cooled to 0 °C, and NaH (60 wt% dispersion in mineral oil, 3.0 g, 72.3 mmol, 1.3 eq.) was added portion wise. The mixture was stirred for 2 hours or until TLC (60:40, *n*-Pentane:EtOAc, v:v) indicated complete conversion of the starting material. Thereafter, the reaction was quenched by the addition of water at 0 °C. The resulting black suspension was extracted twice with DCM. The combined organic layers were washed with sat. aq. $NaHCO_3$, 1.0 M aq. NaOH solution, sat. aq. $Na_2S_2O_3$ solution, brine, dried over $MgSO_4$ and concentrated under reduced pressure. Flash column chromatography (90:10 to 70:30, *n*-Pentane:EtOAc, v:v) and subsequent recrystallization from boiling EtOH afforded the title compound (21.1 g, 47.8 mmol, 86%) as a white solid. Spectroscopic data were in accord with those previously recorded. TLC: R_f 0.49, (70:30, *n*-Pentane:EtOAc, v:v); ^{1}H NMR ($CDCl_3$, 400 MHz, HH-COSY, HSQC): δ 7.55 – 7.45 (m, 2H, CH_{arom}), 7.38 – 7.28 (m, 3H, CH_{arom}), 5.23 (td, *J* = 9.2, 0.9 Hz, 1H, H-3), 5.05 (dd, *J* = 10.1, 9.4 Hz, 1H, H-4), 4.98 (ddd, *J* = 10.1, 9.1, 4.5 Hz, 1H, H-2), 4.71 (dd, *J* = 155.7, 10.3 Hz, 1H, H-1), 4.23 (dd, *J* = 12.3, 5.0 Hz, 1H, H-6), 4.18 (dd, *J* = 12.3, 2.7 Hz, 1H, H-6), 3.73 (ddt, *J* = 10.2, 5.1, 2.6 Hz, 1H, H-5), 2.09 (s, 3H, CH_3 Ac), 2.09 (s, 3H, CH_3 Ac), 2.02 (s, 3H, CH_3 Ac), 2.00 (s, 3H, CH_3 Ac); ^{13}C-APT NMR (101 MHz, $CDCl_3$, HSQC): δ 170.7, 170.3, 169.5, 169.4 (C=O Ac), 133.2 (d, *J* = 1.9 Hz, CH_{arom}), 131.7(d, *J* = 1.1 Hz, C_q), 129.1, 128.5 (CH_{arom}), 85.8 (C-1), 75.9 (d, *J* = 2.1 Hz, C-5), 74.1 (d, *J* = 3.1 Hz, C-3), 70.0 (d, *J* = 44.3 Hz, C-2), 68.3 (C-4), 62.2 (d, *J* = 4.6 Hz, C-6), 20.9, 20.9, 20.7, 20.7 (CH_3 Ac); ^{13}C-GATED NMR (101 MHz, $CDCl_3$): δ 85.8 ($J_{C1,H1}$ = 155.7 Hz, C-1 β);

Phenyl 1-thio-β-D-glucopyranoside[1-^{13}C] (S5)

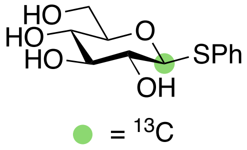

Compound **S4** (21.1 g, 47.8 mmol) was co-evaporated with anhydrous toluene and dissolved in MeOH (400 mL, 0.1 M). To the solution, NaOMe (1.0 g, 19.1 mmol, 0.4 eq.) was added and the reaction mixture was stirred at r.t. for 16 hours or until TLC (90:10, DCM:MeOH, v:v) indicated complete conversion of the starting material. The reaction was quenched by the addition of Amberlite IR120 H^+ until a pH of 4 was reached. The mixture was filtered, and the filtrate was concentrated under reduced pressure. Flash column chromatography (99:1 to 80:20, DCM:MeOH, v:v) afforded the title compound (11.0 g, 43.5 mmol, 91%) as a white solid. Spectroscopic data were in accord with those previously recorded. TLC: R_f 0.49, (80:20, DCM:MeOH, v:v); ^{1}H NMR (MeOD, 400 MHz, HH-COSY, HSQC): δ 7.62 – 7.47 (m, 2H, CH_{arom}), 7.38 – 7.16 (m, 3H, CH_{arom}), 4.59 (dd, *J* = 154.7, 9.6 Hz, 1H, H-1), 3.87 (dd, *J* = 12.2, 1.9 Hz, 1H, H-6), 3.66 (dd, *J* = 12.1, 5.4 Hz, 1H, H-6), 3.38 (td, *J* = 8.2, 1.0 Hz, 1H, H-3), 3.31 – 3.28 (m, 2H, H-4, H-5), 3.21 (ddd, *J* = 9.8, 8.7, 4.8 Hz, 1H, H-2); ^{13}C-APT NMR (101 MHz, $CDCl_3$, HSQC): δ 135.3 (d, *J* = 1.4 Hz, C_q), 132.7, 129.9, 128.3 (CH_{arom}), 89.4 (C-1), 82.0 (d, *J* = 2.1 Hz, C-4), 79.7 (d, *J* = 3.1

Hz, C-3), 73.7 (d, J = 41.4 Hz, C-2), 71.3 (C-5), 62.8 (d, J = 4.6 Hz, C-6); ^{13}C-GATED NMR (101 MHz, $CDCl_3$): δ 89.4 ($J_{C1,H1}$ = 154.7 Hz, C-1 β).

Phenyl 4,6-*O*-benzylidene[α-^{12}C/^{13}C]-1-thio-β-ᴅ-glucopyranoside[1-^{12}C/^{13}C] (S6)

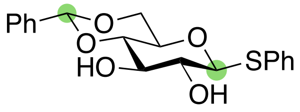

● = ^{12}C:^{13}C (50:50)

S5 (0.9 g, 3.1 mmol, 0.5 eq.) and its unlabeled counterpart, **S2**, (0.9 g, 3.1 mmol, 0.5 eq.) were dissolved a 1:1 ratio in anhydrous DMF (12.5 mL, 0.1 M) and cooled to 0 °C. To the solution, benzaldehyde[α-^{13}C] dimethyl acetal (0.5 mL, 3.7 mmol, 0.6 eq.), unlabeled benzaldehyde dimethyl acetal (0.5 g, 3.7 mmol, 0.6 eq.), and $HBF_4{\cdot}OEt_2$ (0.8 mL, 6.2 mmol, 1.0 eq.) were added and the mixture was allowed to warm up to r.t. After stirring for 16 hours or until TLC (90:10, DCM:MeOH, v:v) indicated complete conversion of the starting material, the reaction was quenched by the addition of Et_3N until a pH of 9 – 10 was reached. The mixture was concentrated under reduced pressure and redissolved in EtOAc. The organic phase was washed with H_2O, brine, dried over Na_2SO_4, filtered and concentrated under reduced pressure. Flash column chromatography (70:30 to 50:50, *n*-Pentane:EtOAc, v:v) afforded the title compound (1.6 g, 4.5 mmol, 72%) as a white solid. TLC: R_f 0.71, (95:5, DCM:MeOH, v:v); ^{1}H NMR ($CDCl_3$, 400 MHz, HH-COSY, HSQC): δ 7.57 – 7.53 (m, 2H, CH_{arom}), 7.48 (m, 2H, CH_{arom}), 7.41 – 7.33 (m, 6H, CH_{arom}), 5.54 (s, 0.5H, C*H*Ph), 5.54 (d, J = 162.6 Hz, 0.5H, C*H*Ph[α-^{13}C]), 4.65 (d, J = 9.7 Hz, 0.5H, H-1), 4.64 (dd, J = 155.9, 9.7 Hz, 0.5H, H-1[1-^{13}C]), 4.42 – 4.36 (m, 1H, H-6), 3.91 – 3.83 (m, 2H, H-3, H-6), 3.59 – 3.51 (m, 2H, H-2, H-4), 3.48 (ddt, J = 10.0, 8.4, 2.0 Hz, 1H, H-5), 2.77 (d, J = 2.2 Hz, 1H, O*H*), 2.65 (d, J = 2.0 Hz, 1H, O*H*); ^{13}C-APT NMR (101 MHz, $CDCl_3$, HSQC): δ 136.9 (C_q), 133.1 (CH_{arom}), 130.6 (C_q), 129.2, 128.5, 128.4, 126.3 (CH_{arom}), 102.0 (CHPh), 88.6 (C-1), 80.2 (C-4), 74.6 (C-3), 72.6 (C-2), 72.6 (d, J = 44.0 Hz, C-2[1-^{13}C]), 70.6 (C-5), 68.6 (C-6), 68.6 (d, J = 4.5 Hz, C-6[1-^{13}C]); ^{13}C-GATED NMR (101 MHz, $CDCl_3$): δ 88.6 ($J_{C1,H1}$ = 155.9 Hz, C-1 β).

Phenyl 2,3-di-*O*-benzyl-4,6-*O*-benzylidene[α-^{12}C/^{13}C]-1-thio-β-ᴅ-glucopyranoside[1-^{12}C/^{13}C] (S7)

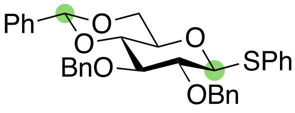

● = ^{12}C:^{13}C (50:50)

Compound **S6** (1.6 g, 4.5 mmol) was co-evaporated with anhydrous toluene, dissolved in dry DMF (22.5 mL, 0.2 M) and cooled to 0 °C. To the solution, NaH (60 wt% dispersion in mineral oil, 0.5 g, 11.7 mmol, 2.6 eq.) and BnBr (1.2 mL, 9.9 mmol, 2.2 eq.) were added, and the reaction mixture was allowed to warm up to r.t. After stirring for 16 hours or until TLC (70:30, *n*-Pentane:Et_2O, v:v) indicated complete conversion of the starting material. The reaction was quenched by the addition of MeOH at 0 °C. The aqueous phase was extracted thrice with Et_2O, and the combined organic layers were washed with sat. aq. $NaHCO_3$ solution, brine, dried over $MgSO_4$, filtered and concentrated under reduced pressure. Recrystallization from EtOAc:*n*-Pentane afforded the title compound (1.6 g, 2.9 mmol, 65%) as a fluffy white solid. TLC: R_f 0.69, (80:20, *n*-Pentane:EtOAc, v:v); ^{1}H NMR (500 MHz, $CDCl_3$, HH-COSY, HSQC): δ 7.58 – 7.23 (m, 20H, CH_{arom}), 5.59 (s, 0.5H, C*H*Ph), 5.59 (d = 161.8 Hz, 0.5H, C*H*Ph[α-^{13}C]), 4.94 (d, J = 11.2 Hz, 1H, C*H*H Bn), 4.86 (d, J = 10.3 Hz, 1H, C*H*H Bn), 4.82 (d, J = 10.3 Hz, 1H, CH*H* Bn), 4.78 (d, J = 11.1 Hz, 1H, CH*H* Bn), 4.76 (d, J = 9.8 Hz, 1H, H-1), 4.76 (d, J = 156.9, 9.8 Hz, 1H, H-1[1-^{13}C]), 4.39 (dd, J = 10.5, 5.0 Hz, 1H, H-6), 3.84 (dd, J = 9.4, 8.3 Hz, 1H, H-3), 3.80 (t, J = 10.3 Hz, 1H, H-6), 3.71 (t, J = 9.4 Hz, 1H, H-4), 3.58 – 3.45 (m, 2H, H-2, H-5); ^{13}C-APT NMR (126 MHz, $CDCl_3$, HSQC): δ 138.4, 138.2, 137.4, 133.2 (C_q), 132.5, 129.2, 129.1, 128.5, 128.5, 128.4, 128.4,

128.3, 128.0, 128.0, 127.9, 126.1 (CH_{arom}), 101.3 (CHPh), 88.4 (C-1), 83.2 (C-3), 81.6 (C-4), 80.6 (C-2), 80.6 (d, J = 40.4 Hz, C-2[1-^{13}C]), 76.0 (CH_2 Bn), 75.5 (CH_2 Bn), 70.4 (C-5), 68.9 (C-6), 68.9 (d, J = 4.2 Hz, C-6[1-^{13}C]); ^{13}C-GATED NMR (126 MHz, $CDCl_3$): δ 88.4 ($J_{C1,H1}$ = 156.9 Hz, C-1 β);

Glycosylation Products

Ethyl 2,3-di-*O*-benzyl-4,6-*O*-benzylidene-α/β-D-glucopyranoside (S8)

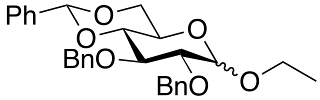

The title compound was prepared from donor **1** and ethanol using the general procedure for Tf_2O/Ph_2SO mediated glycosylations. Flash column chromatography (80:20 to 60:40, *n*-Pentane:Et_2O, v:v) yielded glycosylation product **S8** (40 mg, 84 μmol, 84%, α:β = 20:80) as a white solid. Spectroscopic data were in accord with those previously recorded. TLC: R_f 0.48, (80:20, *n*-Pentane:EtOAc, v:v); Data for the β-anomer: ^{1}H NMR (500 MHz, $CDCl_3$, HH-COSY, HSQC, HMBC): δ 7.53 – 7.21 (m, 15H, CH_{arom}), 5.56 (s, 1H, C*H*Ph), 4.92 (d, *J* = 10.9 Hz, 1H, C*H*H Bn), 4.91 (d, *J* = 11.1 Hz, 1H, C*H*H Bn), 4.80 (d, *J* = 11.5 Hz, 1H, CH*H* Bn), 4.77 (d, *J* = 10.9 Hz, 1H, CH*H* Bn), 4.51 (d, *J* = 7.7 Hz, 1H, H-1), 4.35 (dd, *J* = 10.5, 5.0 Hz, 1H, H-6), 3.98 (dq, *J* = 9.4, 7.0 Hz, 1H, C*H*HCH_3), 3.79 (t, *J* = 10.3 Hz, 1H, H-6), 3.74 (t, J = 9.0 Hz, 1H, H-3), 3.68 (dd, *J* = 9.9, 8.8 Hz, 1H, H-4), 3.66 (dq, *J* = 9.4, 7.0 Hz, 1H, CH*H*CH_3), 3.46 (t, *J* = 8.2 Hz, 1H, H-2), 3.40 (td, *J* = 9.7, 5.0 Hz, 1H, H-5), 1.29 (t, *J* = 7.1 Hz, 3H, CH_2CH_3); ^{13}C-APT NMR (126 MHz, $CDCl_3$, HSQC, HMBC): δ 138.7, 138.6, 137.5 (C_q), 129.0, 128.5, 128.4, 128.4, 128.3, 128.1, 127.8, 127.7, 126.1 (CH_{arom}), 104.1 (C-1), 101.3 (CHPh), 82.3 (C-2), 81.6 (C-4), 81.0 (C-3), 75.5 (CH_2 Bn), 75.2 (CH_2 Bn), 69.0 (C-6), 66.2 (CH_2CH_3), 66.1 (C-5), 15.5 (CH_2CH_3); ^{13}C-GATED NMR (126 MHz, $CDCl_3$): δ 104.1 ($J_{C1,H1}$ = 160.1 Hz, C-1 β); Data for the α-anomer: ^{1}H NMR (500 MHz, $CDCl_3$, HH-COSY, HSQC, HMBC): δ 7.53 – 7.21 (m, 15H, CH_{arom}), 5.55 (s, 1H, C*H*Ph), 4.92 (d, *J* = 11.2 Hz, 1H, C*H*H Bn), 4.85 (d, *J* = 12.2 Hz, 1H, C*H*H Bn), 4.85 (d, *J* = 11.2 Hz, 1H, CH*H* Bn), 4.73 (d, *J* = 3.7 Hz, 1H, H-1), 4.69 (d, *J* = 12.1 Hz, 1H, CH*H* Bn), 4.25 (dd, *J* = 10.2, 4.8 Hz, 1H, H-6), 4.06 (t, *J* = 9.3 Hz, 1H, H-3), 3.88 (td, *J* = 10.0, 4.9 Hz, 1H, H-5), 3.73 (dq, *J* = 9.4, 7.1 Hz, 1H, C*H*HCH_3), 3.70 (t, *J* = 10.4 Hz, 1H, H-6), 3.61 (t, *J* = 9.4 Hz, 1H, H-4), 3.55 (dd, *J* = 9.3, 3.7 Hz, 1H, H-2), 3.53 (dq, *J* = 9.3, 7.0 Hz, 1H, CH*H*CH_3), 1.28 (t, *J* = 7.1 Hz, 3H, CH_2CH_3); ^{13}C-APT NMR (126 MHz, $CDCl_3$, HSQC, HMBC): δ 139.0, 138.4, 137.6 (C_q), 129.0, 128.6, 128.4, 128.4, 128.2, 128.1, 128.0, 127.7, 126.1 (CH_{arom}), 101.3 (CHPh), 97.8 (C-1), 82.4 (C-4), 79.5 (C-2), 78.8 (C-3), 75.5 (CH2 Bn), 73.8 (CH2 Bn), 69.2 (C-6), 63.8 (CH_2CH_3), 62.5 (C-5), 15.2 (CH_2CH_3); ^{13}C-GATED NMR (126 MHz, $CDCl_3$): δ 97.8 ($J_{C1,H1}$ = 168.1 Hz, C-1 α); HRMS: $[M+H]^+$ calcd. for $C_{29}H_{33}O_6^+$ 477.2272, found 477.2270.

2-Fluoroethyl 2,3-di-*O*-benzyl-4,6-*O*-benzylidene-α/β-D-glucopyranoside (S9)

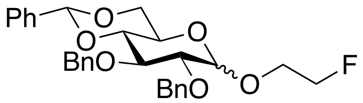

The title compound was prepared from donor **1** and 2-fluoroethanol using the general procedure for Tf_2O/Ph_2SO mediated glycosylations. Flash column chromatography (80:20 to 60:40, *n*-Pentane:Et_2O, v:v) yielded glycosylation product **S9** (32 mg, 65 μmol, 65%, α:β = 48:52) as a white solid. Spectroscopic data were in accord with those previously recorded. TLC: R_f 0.23, (80:20, *n*-Pentane:EtOAc, v:v); Data for the β-anomer: ^{1}H NMR (500 MHz, $CDCl_3$, HH-COSY, HSQC, HMBC): δ 7.53 – 7.21 (m, 15H, CH_{arom}), 5.56 (s, 1H, C*H*Ph), 4.92 (d, *J* = 11.1 Hz, 1H, C*H*H Bn), 4.91 (d, *J* = 11.4 Hz, 1H, C*H*H Bn), 4.81 (d, *J* = 11.4 Hz, 1H,

CH*H* Bn), 4.77 (d, *J* = 10.8 Hz, 1H, CH*H* Bn), 4.61 (ddd, *J* = 47.2, 5.2, 2.6 Hz, 2H, CH_2CH_2F), 4.56 (d, *J* = 7.7 Hz, 1H, H-1), 4.35 (dd, *J* = 10.3, 4.8 Hz, 1H, H-6), 4.10 (dddd, *J* = 32.3, 12.4, 5.1, 2.4 Hz, 1H, C*H*HCH_2F), 3.88 (dddd, *J* = 26.1, 12.0, 6.8, 3.5 Hz, 1H, CH*H*CH_2F), 3.79 (t, *J* = 10.3 Hz, 1H, H-6), 3.75 (t, *J* = 9.1 Hz, 1H, H-3), 3.69 (t, *J* = 9.3 Hz, 1H, H-4), 3.50 (dd, *J* = 8.8, 7.7 Hz, 1H, H-2), 3.42 (td, *J* = 9.6, 5.0 Hz, 1H, H-5); ^{13}C-APT NMR (126 MHz, $CDCl_3$, HSQC, HMBC): δ 138.5, 138.3, 137.3 (C_q), 129.0, 128.4, 128.3, 128.3, 128.3, 128.0, 127.8, 127.7, 126.0 (CH_{arom}), 104.3 (C-1), 101.2 (CHPh), 82.5 (d, *J* = 170.2 Hz, CH_2CH_2F), 82.0 (C-2), 81.4 (C-4), 80.8 (C-3), 75.4 (CH_2 Bn), 75.2 (CH_2 Bn), 69.3 (d, *J* = 20.0 Hz, CH_2CH_2F), 68.7 (C-6), 66.1 (H-5); ^{13}C-GATED NMR (126 MHz, $CDCl_3$): δ 104.3 ($J_{C1,H1}$ = 159.7 Hz, C-1 β); Data for the α-anomer: ^{1}H NMR (500 MHz, $CDCl_3$, HH-COSY, HSQC, HMBC): δ 7.53 – 7.21 (m, 15H, CH_{arom}), 5.55 (s, 1H, C*H*Ph), 4.92 (d, *J* = 11.5 Hz, 1H, C*H*H Bn), 4.85 (d, *J* = 12.2 Hz, 1H, C*H*H Bn), 4.85 (d, *J* = 11.1 Hz, 1H, CH*H* Bn), 4.79 (d, *J* = 3.7 Hz, 1H, H-1), 4.70 (d, *J* = 12.0 Hz, 1H, CH*H* Bn), 4.62 (ddd, *J* = 47.6, 4.7, 3.7 Hz, 2H, CH2C*H*$_2$F), 4.26 (dd, *J* = 10.2, 4.8 Hz, 1H, H-6), 4.07 (t, *J* = 9.3 Hz, 1H, H-3), 3.91 (td, *J* = 10.1, 5.0 Hz, 1H, H-5), 3.87 (dddd, *J* = 19.1, 12.6, 6.7, 2.7 Hz, 1H, C*H*HCH_2F), 3.78 (dddd, *J* = 32.0, 12.4, 5.1, 2.5 Hz, 1H, CH*H*CH_2F), 3.70 (t, *J* = 10.3 Hz, 1H, H-6), 3.62 (t, *J* = 9.5 Hz, 1H, H-4), 3.58 (dd, *J* = 9.4, 3.8 Hz, 1H, H-2); ^{13}C-APT NMR (126 MHz, $CDCl_3$, HSQC, HMBC): δ 138.9, 138.3, 137.5 (C_q), 129.1, 128.6, 128.5, 128.4, 128.2, 128.1, 128.1, 127.7, 126.2 (CH_{arom}), 101.4 (CHPh), 98.5 (C-1), 82.7 (d, *J* = 170.2 Hz, CH_2CH_2F), 82.2 (C-4), 79.4 (C-2), 78.6 (C-3), 75.5 (CH_2 Bn), 73.8 (CH_2 Bn), 69.1 (C-6), 67.3 (d, *J* = 20.1 Hz, CH_2CH_2F), 62.7 (C-5); ^{13}C-GATED NMR (126 MHz, $CDCl_3$): δ 98.5 ($J_{C1,H1}$ = 168.9 Hz, C-1 α); HRMS: $[M+H]^+$ calcd. for $C_{29}H_{32}FO_6^+$ 495.2177, found 495.2175.

2,2-Difluoroethyl 2,3-di-*O*-benzyl-4,6-*O*-benzylidene-α/β-D-glucopyranoside (S10)

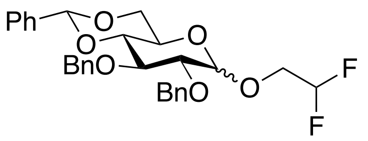

The title compound was prepared from donor **1** and 2,2-difluoroethanol using the general procedure for Tf_2O/Ph_2SO mediated glycosylations. Flash column chromatography (80:20 to 60:40, *n*-Pentane:Et_2O, v:v) yielded glycosylation product **S10** (49 mg, 95 μmol, 95%, α:β = 85:15) as a white solid. Spectroscopic data were in accord with those previously recorded. TLC: R_f 0.31, (80:20, *n*-Pentane:EtOAc, v:v); Data for the α-anomer: ^{1}H NMR (500 MHz, $CDCl_3$, HH-COSY, HSQC, HMBC): δ 7.54 – 7.29 (m, 15H, CH_{arom}), 5.99 (tt, *J* = 55.4, 4.3 Hz, 1H, CH_2CHF_2), 5.59 (s, 1H, C*H*Ph), 4.95 (d, *J* = 11.2 Hz, 1H, C*H*H Bn), 4.88 (d, *J* = 12.0 Hz, 1H, C*H*H Bn), 4.87 (d, J = 11.3 Hz, CH*H* Bn), 4.79 (d, *J* = 3.8 Hz, 1H, H-1), 4.70 (d, *J* = 12.0 Hz, 1H, CH*H* Bn), 4.29 (dd, *J* = 10.3, 4.9 Hz, 1H, H-6), 4.07 (t, *J* = 9.3 Hz, 1H, H-3), 3.89 (td, *J* = 10.0, 5.0 Hz, 1H, H-5), 3.82 (dddd, *J* = 24.1, 12.6, 7.1, 4.3 Hz, 1H, C*H*HCHF_2), 3.78 (dddd, *J* = 23.4, 12.1, 7.1, 4.2 Hz, 1H, CH*H*CHF_2), 3.73 (t, *J* = 10.3 Hz, 1H, H-6), 3.66 (t, *J* = 9.5 Hz, 1H, H-4), 3.61 (dd, J = 9.4, 3.8 Hz, 1H, H-2); ^{13}C-APT NMR (126 MHz, $CDCl_3$, HSQC, HMBC): δ 138.8, 138.2, 137.4 (C_q), 129.1, 128.7, 128.5, 128.4, 128.2, 128.2, 128.1, 127.8, 126.1 (CH_{arom}), 114.2 (t, *J* = 241.5 Hz, CH_2CHF_2), 101.4 (CHPh), 98.9 (C-1), 82.0 (C-4), 79.2 (C-2), 78.4 (C-3), 75.5 (CH_2 Bn), 74.0 (CH_2 Bn), 68.9 (C-6), 67.4 (t, J = 28.8 Hz, CH_2CHF_2), 63.0 (C-5); ^{13}C-GATED NMR (126 MHz, $CDCl_3$): δ 98.9 ($J_{C1,H1}$ = 169.4 Hz, C-1 α); Data for the β-anomer: ^{1}H NMR (500 MHz, $CDCl_3$, HH-COSY, HSQC, HMBC): δ 7.53 – 7.29 (m, 15H, CH_{arom}), 5.94 (dddd, *J* = 55.8, 54.8, 4.9, 3.3 Hz, 1H, CH_2CHF_2), 5.59 (s, 1H, C*H*Ph), 4.94 (d, *J* = 11.4 Hz, 1H, C*H*H Bn), 4.88 (d, *J* = 10.8 Hz, 1H, C*H*H Bn), 4.83 (d, *J* = 11.4 Hz, 1H, CH*H* Bn), 4.79 (d, *J* = 10.8 Hz, 1H, CH*H* Bn), 4.57 (d, *J* = 7.7 Hz, 1H, H-1), 4.37 (dd, *J* = 10.5, 4.9 Hz, 1H, H-6), 4.04 (dddd, *J* = 30.1, 24.3, 9.8, 4.9 Hz, 1H, C*H*HCHF_2), 3.85 (dddd, *J* = 36.0, 23.8, 10.0, 3.4 Hz, 1H, CH*H*CHF_2), 3.81 (t,

J = 10.3 Hz, 1H, H-6), 3.78 (t, J = 9.0 Hz, 1H, H-3), 3.71 (t, J = 9.3 Hz, 1H, H-4), 3.51 (dd, J = 8.7, 7.6 Hz, 1H, H-2), 3.44 (ddd, J = 10.4, 9.3, 4.9 Hz, 1H, H-5); ^{13}C-APT NMR (126 MHz, $CDCl_3$, HSQC, HMBC): δ 138.4, 138.1, 137.2 (C_q), 129.0, 128.4, 128.3, 128.3, 128.2, 128.0, 127.9, 127.7, 126.0 (CH_{arom}), 114.0 (t, J = 241.1 Hz, CH_2CHF_2), 104.3 (C-1), 101.2 (CHPh), 81.8 (C-2), 81.3 (C-4), 80.7 (C-3), 75.5 (CH_2 Bn), 75.2 (CH_2 Bn), 68.9 (dd, J = 29.6, 27.1 Hz, CH_2CHF_2), 68.6 (C-6), 66.2 (C-5); ^{13}C-GATED NMR (126 MHz, $CDCl_3$): δ 104.3 ($J_{C1,H1}$ = 161.5 Hz, C-1 β); HRMS: $[M+H]^+$ calcd. for $C_{29}H_{31}F_2O_6^+$ 513.2083, found 513.2081.

2,2,2-Trifluoroethyl 2,3-di-*O*-benzyl-4,6-*O*-benzylidene-α/β-D-glucopyranoside (S11)

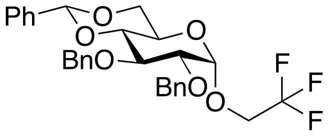

The title compound was prepared from donor **1** and 2,2,2-trifluoroethanol using the general procedure for Tf_2O/Ph_2SO mediated glycosylations. Flash column chromatography (80:20 to 60:40, *n*-Pentane:Et_2O, v:v) yielded glycosylation product **S11** (49 mg, 95 µmol, 95%, α:β = >98:2) as a white solid. Spectroscopic data were in accord with those previously recorded. TLC: R_f 0.38, (80:20, *n*-Pentane:EtOAc, v:v); ^{1}H NMR (500 MHz, $CDCl_3$, HH-COSY, HSQC, HMBC): δ 7.50 – 7.26 (m, 15H), 5.55 (s, 1H), 4.92 (d, J = 11.2 Hz, 1H, C*H*H Bn), 4.84 (d, J = 12.0 Hz, 1H, C*H*H Bn), 4.84 (d, J = 11.2 Hz, 1H, CH*H* Bn), 4.80 (d, J = 3.8 Hz, 1H, H-1), 4.67 (d, J = 12.0 Hz, 1H, CH*H* Bn), 4.26 (dd, J = 10.2, 4.9 Hz, 1H, H-6), 4.05 (t, J = 9.3 Hz, 1H, H-3), 3.93 (dq, J = 8.7, 2.0 Hz, 2H, CH_2CF_3), 3.85 (td, J = 10.1, 5.4 Hz, 1H, H-5), 3.70 (t, J = 10.4 Hz, 1H, H-6), 3.63 (t, J = 9.5 Hz, 1H, H-4), 3.59 (dd, J = 9.3, 3.8 Hz, 2H, H-2); ^{13}C-APT NMR (126 MHz, $CDCl_3$, HSQC, HMBC): δ 138.6, 138.0, 137.2 (C_q), 129.0, 128.5, 128.3, 128.3, 128.1, 128.0, 128.0, 127.7, 126.0 (CH_{arom}), 123.7 (q, J = 278.9 Hz, CH_2CF_3), 101.3 (CHPh), 98.8 (C-1), 81.8 (C-4), 79.0 (C-2), 78.2 (C-3), 75.4 (CH_2 Bn), 73.7 (CH_2 Bn), 68.7 (C-6), 65.1 (q, J = 35.0 Hz, CH_2CF_3), 63.1 (C-5); ^{13}C-GATED NMR (126 MHz, $CDCl_3$): δ 98.8 ($J_{C1,H1}$ = 170.4 Hz, C-1 α); HRMS: $[M+H]^+$ calcd. for $C_{29}H_{30}F_3O_6^+$ 531.1989, found 531.1986.

***Iso*-propyl 2,3-di-*O*-benzyl-4,6-*O*-benzylidene-α/β-D-glucopyranoside (S12)**

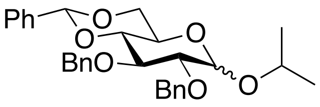

The title compound was prepared from donor **1** and *iso*-propanol using the general procedure for Tf_2O/Ph_2SO mediated glycosylations. Flash column chromatography (80:20 to 60:40, *n*-Pentane:Et_2O, v:v) yielded glycosylation product **S12** (40 mg, 82 µmol, 82%, α:β = 36:64) as a white solid. Spectroscopic data were in accord with those previously recorded. TLC: R_f 0.40, (80:20, *n*-Pentane:EtOAc, v:v); Data for the β-anomer: ^{1}H NMR (500 MHz, $CDCl_3$, HH-COSY, HSQC, HMBC): δ 7.50 – 7.47 (m, 2H, CH_{arom}), 7.39 – 7.26 (m, 13H, CH_{arom}), 5.56 (s, 1H, C*H*Ph), 4.92 (d, J = 10.8 Hz, 1H, C*H*H Bn), 4.90 (d, J = 11.4 Hz, 1H, C*H*H Bn), 4.79 (d, J = 11.4 Hz, 1H, CH*H* Bn), 4.76 (d, J = 10.8 Hz, 1H, CH*H* Bn), 4.58 (d, J = 7.7 Hz, 1H, H-1), 4.33 (dd, J = 10.4, 4.9 Hz, 1H, H-6), 4.01 (h, J = 6.2 Hz, 1H, C*H*$(CH_3)_2$ ^{i}Pr), 3.80 (t, J = 10.3 Hz, 1H, H-6), 3.73 (t, J = 9.1 Hz, 1H, H-3), 3.68 (t, J = 9.3 Hz, 1H, H-4), 3.45 (dd, J = 8.8, 7.8 Hz, 1H, H-2), 3.85 3.40 (ddd, J = 10.1, 9.0, 5.0 Hz, 1H, H-5), 1.28 (d, J = 6.2 Hz, 3H, CH(C*H*$_3$)$_2$ ^{i}Pr), 1.25 (d, J = 6.3 Hz, 3H, CH(C*H*$_3$)$_2$ ^{i}Pr); ^{13}C-APT NMR (126 MHz, $CDCl_3$, HSQC, HMBC): δ 139.1, 138.6, 137.6 (C_q), 129.0, 128.5, 128.4, 128.4, 128.2, 128.1, 127.8, 127.7, 126.1 (CH_{arom}), 102.7 (C-1), 101.3 (CHPh), 82.3 (C-2), 81.6 (C-4), 81.2 (C-3), 75.5 (CH_2 Bn), 75.2 (CH_2 Bn), 72.8 (*C*H$(CH_3)_2$ ^{i}Pr), 69.0 (C-6), 66.2 (C-5), 23.8 (CH$(CH_3)_2$ ^{i}Pr), 22.3 (CH(*C*H$_3$)$_2$ ^{i}Pr); ^{13}C-GATED NMR (126 MHz, $CDCl_3$): δ 102.7 ($J_{C1,H1}$ = 158.9 Hz, C-1 β); Data for the α-anomer: ^{1}H NMR (500 MHz, $CDCl_3$, HH-COSY, HSQC, HMBC): δ 7.54 – 7.51 (m,

2H, CH_{arom}), 7.43 – 7.29 (m, 13H, CH_{arom}), 5.56 (s, 1H, C*H*Ph), 4.93 (d, *J* = 11.1 Hz, 1H, C*H*H Bn), 4.85 (d, *J* = 11.2 Hz, 1H, CH*H* Bn), 4.84 (d, *J* = 3.9 Hz, 1H, H-1), 4.82 (d, *J* = 11.2 Hz, 1H, C*H*H Bn), 4.69 (d, *J* = 12.1 Hz, 1H, CH*H* Bn), 4.25 (dd, *J* = 10.2, 4.9 Hz, 1H, H-6), 4.06 (t, *J* = 9.3 Hz, 1H, H-3), 3.93 (td, *J* = 10.1, 4.9 Hz, 1H, H-5), 3.88 (h, *J* = 6.2 Hz, 1H, C*H*(CH_3)$_2$ ^{i}Pr), 3.70 (t, *J* = 10.4 Hz, 1H, H-6), 3.61 (t, *J* = 9.4 Hz, 1H, H-4), 3.55 (dd, *J* = 9.3, 3.8 Hz, 1H, H-2), 1.27 (d, J = 6.3 Hz, 3H, CH(C*H*$_3$)$_2$ ^{i}Pr), 1.21 (d, J = 6.2 Hz, 3H, CH(C*H*$_3$)$_2$ ^{i}Pr); ^{13}C-APT NMR (126 MHz, $CDCl_3$, HSQC, HMBC): δ 138.9, 138.3, 137.4 (C_q), 128.9, 128.4, 128.3, 128.2, 128.1, 128.0, 127.9, 127.6, 126.0 (CH_{arom}), 101.3 (CHPh), 96.3 (C-1), 82.6 (C-4), 79.4 (C-2), 78.9 (C-3), 75.5 (CH_2 Bn), 73.6 (CH_2 Bn), 70.0 (*C*H(CH_3)$_2$ ^{i}Pr), 69.2 (C-6), 62.5 (C-5), 23.4 (CH(*C*H$_3$)$_2$ ^{i}Pr), 21.5 (CH(*C*H$_3$)$_2$ ^{i}Pr); ^{13}C-GATED NMR (126 MHz, $CDCl_3$): δ 96.3 ($J_{C1,H1}$ = 166.8 Hz, C-1 α).

***cyclo*-Hexyl 2,3-di-*O*-benzyl-4,6-*O*-benzylidene-α/β-D-glucopyranoside (S13)**

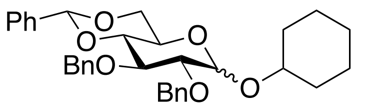

The title compound was prepared from donor **1** and *cyclo*-hexanol using the general procedure for Tf_2O/Ph_2SO mediated glycosylations. Flash column chromatography (80:20 to 60:40, *n*-Pentane:Et_2O, v:v) yielded glycosylation product **S13** (XX mg, 89 µmol, 89%, α:β = 40:60) as a white solid. Spectroscopic data were in accord with those previously recorded. TLC: R_f 0.42, (80:20, *n*-Pentane:EtOAc, v:v); Data for the β-anomer: ^{1}H NMR (500 MHz, $CDCl_3$, HH-COSY, HSQC, HMBC): δ 7.54 – 7.21 (m, 15H, CH_{arom}), 5.56 (s, 1H, C*H*Ph), 4.94 (d, *J* = 10.9 Hz, 1H, C*H*H Bn), 4.90 (d, *J* = 11.4 Hz, 1H, C*H*H Bn), 4.79 (d, *J* = 11.4 Hz, 1H, CH*H* Bn), 4.76 (d, *J* = 10.8 Hz, 1H, CH*H* Bn), 4.62 (d, *J* = 7.8 Hz, 1H, H-1), 4.33 (dd, *J* = 10.5, 5.0 Hz, 1H, H-6), 3.79 (t, *J* = 10.3 Hz, 1H, H-6), 3.75 – 3.66 (m, 3H, H-3, H-4, CH Cy), 3.46 (t, *J* = 8.2 Hz, 1H, H-2), 3.39 (ddd, *J* = 10.0, 8.9, 5.0 Hz, 1H, H-5), 2.01 – 1.18 (m, 10H, 5x CH_2 Cy); ^{13}C-APT NMR (126 MHz, $CDCl_3$, HSQC, HMBC): δ 138.8, 138.6, 137.5 (C_q), 129.0, 128.5, 128.4, 128.3, 128.3, 128.1, 127.8, 127.7, 126.1 (CH_{arom}), 102.5 (C-1), 101.2 (CHPh), 82.3 (C-2), 81.6 (C-4), 81.2 (C-3), 78.3 (CH Cy), 75.5 (CH_2 Bn), 75.2 (CH_2 Bn), 69.0 (C-6), 66.1 (C-5), 33.9 (CH_2 Cy), 32.1 (CH_2 Cy), 25.7 (CH_2 Cy); ^{13}C-GATED NMR (126 MHz, $CDCl_3$): δ 102.5 ($J_{C1,H1}$ = 159.2 Hz, C-1 β); Data for the α-anomer: ^{1}H NMR (500 MHz, $CDCl_3$, HH-COSY, HSQC, HMBC): δ 4.84 (d, *J* = 11.2 Hz, 1H, C*H*H bn), 4.69 (d, *J* = 12.1 Hz, 1H, CH*H* Bn), 4.26 (dd, *J* = 10.2, 4.9 Hz, 1H, H-6), 4.07 (t, *J* = 9.3 Hz, 1H, H-3), 3.96 (d, *J* = 4.8 Hz, 1H, H-5), 3.61 (t, *J* = 9.4 Hz, 1H, H-4), 3.55 (dd, *J* = 9.3, 3.8 Hz, 2H, H-2); ^{13}C-APT NMR (126 MHz, $CDCl_3$, HSQC, HMBC): δ 101.3 (CHPh), 96.2 (C-1), 82.6 (C-4), 79.5 (C-2), 78.8 (C-3), 76.1 (CH Cy), 75.4 (CH_2 Bn), 73.4 (CH_2 Bn), 69.2 (C-6), 62.6 (C-5), 33.6 (CH_2 Cy), 31.6 (CH_2 Cy), 24.2 (CH_2 Cy); ^{13}C-GATED NMR (126 MHz, $CDCl_3$): δ 96.2 ($J_{C1,H1}$ = 168.0 Hz, C-1 α).

***tert*-Butyl 2,3-di-*O*-benzyl-4,6-*O*-benzylidene-α/β-D-glucopyranoside (S14)**

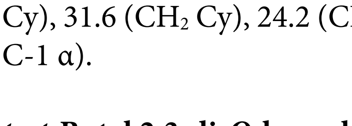

The title compound was prepared from donor **1** and *tert*-butanol using the general procedure for Tf_2O/Ph_2SO mediated glycosylations. Flash column chromatography (80:20 to 60:40, *n*-Pentane:Et_2O, v:v) yielded glycosylation product **S14** (XX mg, XX µmol, XX%, α:β = XX:XX) as a white solid. Spectroscopic data were in accord with those previously recorded. TLC: R_f 0.45, (80:20, *n*-Pentane:EtOAc, v:v); Data for the β-anomer: ^{1}H NMR (500 MHz, $CDCl_3$, HH-COSY, HSQC, HMBC): δ 7.55 – 7.22 (m, 15H, CH_{arom}), 5.55 (s, 1H, C*H*Ph), 4.95 – 4.87 (m, 2H, CH_2 Bn), 4.86 – 4.74 (m, 2H, CH_2 Bn), 4.68 (d, *J* = 7.8 Hz, 1H, H-1), 4.29 (dd, *J* = 10.5, 5.0 Hz, 1H, H-6), 3.79 (t, *J*

= 10.3 Hz, 1H, H-6), 3.75 (t, *J* = 9.0 Hz, 1H, H-3), 3.68 (t, *J* = 9.3 Hz, 1H, H-4), 3.47 – 3.40 (dd, *J* = 7.8, 8.5, 1H, H-2), 3.39 (dt, *J* = 9.7, 4.9 Hz, 1H, H-5), 1.30 (s, 9H, $C(CH_3)_3$ ^{t}Bu); ^{13}C-APT NMR (126 MHz, $CDCl_3$, HSQC, HMBC): δ 138.8, 138.5, 137.6 (C_q), 129.0, 128.4, 128.4, 128.3, 128.2, 128.1, 127.8, 127.7, 126.2 (CH_{arom}), 101.2 (CHPh), 98.5 (C-1), 82.4 (C-2), 81.7 (C-4), 81.5 (C-3), 76.5 (*C*$(CH_3)_3$ ^{t}Bu), 75.6 (CH_2 Bn), 75.3 (CH_2 Bn), 69.1 (C-6), 66.1 (C-5), 29.0 (C(*C*$H_3)_3$ ^{t}Bu); ^{13}C-GATED NMR (126 MHz, $CDCl_3$): δ 98.5 ($J_{C1,H1}$ = 157.8 Hz, C-1 β); Diagnostic peaks α-anomer: ^{1}H NMR (500 MHz, $CDCl_3$, HH-COSY, HSQC, HMBC): δ 7.55 – 7.22 (m, 15H, CH_{arom}), 5.57 (s, 1H, C*H*Ph), 5.08 (d, *J* = 3.8 Hz, 1H, H-1), 4.93 (d, *J* = 11.2 Hz, 1H, C*H*H Bn), 4.83 (d, *J* = 11.1 Hz, 1H, CH*H* Bn), 4.79 (d, *J* = 11.9 Hz, 1H, C*H*H Bn), 4.70 (d, *J* = 11.9 Hz, 1H, CH*H* Bn), 4.22 (dd, *J* = 10.2, 5.0 Hz, 1H, H-6), 4.07 (t, *J* = 9.3 Hz, 1H, H-3), 4.03 (dd, *J* = 10.0, 4.9 Hz, 1H, H-5), 3.79 (t, *J* = 10.3 Hz, 1H, H-6), 3.60 (t, *J* = 9.4 Hz, 1H, H-4), 3.53 (dd, *J* = 9.4, 3.8 Hz, 1H, H-2), 1.28 (s, 9H, $C(CH_3)_3$ ^{t}Bu); ^{13}C-APT NMR (126 MHz, $CDCl_3$, HSQC, HMBC): δ 101.2 (CHPh), 92.8 (C-1), 82.9 (C-4), 79.6 (C-2), 78.9 (C-3), 75.7 (*C*$(CH_3)_3$ ^{t}Bu), 75.4 (CH_2 Bn), 73.6 (CH_2 Bn), 69.3 (C-6), 62.2 (C-5), 28.8 (C(*C*$H_3)_3$ ^{t}Bu); ^{13}C-GATED NMR (126 MHz, $CDCl_3$): δ 92.8 ($J_{C1,H1}$ = 166.2 Hz, C-1 α).

Adamantyl 2,3-di-*O*-benzyl-4,6-*O*-benzylidene-α/β-D-glucopyranoside (S15)

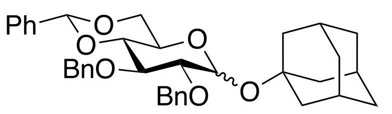

The title compound was prepared from donor **1** and 1-adamantanol using the general procedure for Tf_2O/Ph_2SO mediated glycosylations. Flash column chromatography (80:20 to 60:40, *n*-Pentane:Et_2O, v:v) yielded glycosylation product **S15** (47 mg, 81 µmol, 81%, α:β = 78:22) as a white solid. TLC: R_f 0.48, (80:20, *n*-Pentane:EtOAc, v:v); Data for the α-anomer: ^{1}H NMR (500 MHz, $CDCl_3$, HH-COSY, HSQC, HMBC): δ 7.53 – 7.23 (m, 15H, CH_{arom}), 5.56 (s, 1H, C*H*Ph), 5.23 (d, *J* = 3.8 Hz, 1H, H-1), 4.92 (d, *J* = 11.2 Hz, 1H, C*H*H Bn), 4.83 (d, *J* = 11.2 Hz, 1H, C*H*H Bn), 4.77 (d, *J* = 12.0 Hz, 1H, CH*H* Bn), 4.70 (d, *J* = 12.1 Hz, 1H, CH*H* Bn), 4.23 (dd, *J* = 10.2, 4.9 Hz, 1H, H-6), 4.12 – 4.03 (m, 2H, H-3, H-5), 3.67 (t, *J* = 10.3 Hz, 1H, H-6), 3.60 (t, *J* = 9.4 Hz, 1H, H-4), 3.52 (dd, *J* = 9.4, 3.8 Hz, 1H, H-2), 2.19 – 1.09 (m, 3H, CH Adamantyl), 1.92 – 1.77 (m, 6H, CH_2 Adamantyl), 1.71 – 1.54 (m, 6H, CH_2 Adamantyl); ^{13}C-APT NMR (126 MHz, $CDCl_3$, HSQC, HMBC): δ 139.2, 138.5, 137.7 (C_q), 129.0, 128.5, 128.4, 128.3, 128.2, 128.1, 127.9, 127.6, 126.1 (CH_{arom}), 101.3 (CHPh), 91.2 (C-1), 82.9 (C-4), 79.6 (C-2), 78.8 (C-3), 75.6 (C_q Adamantyl), 75.4 (CH_2 Bn), 73.4 (CH_2 Bn), 69.3 (C-6), 62.2 (C-5), 42.7 (CH Adamantyl), 36.4 (CH_2 Adamantyl), 30.8 (CH_2 Adamantyl); ^{13}C-GATED NMR (126 MHz, $CDCl_3$): δ 91.2 ($J_{C1,H1}$ = 166.9 Hz, C-1 α); Diagnostic peaks β-anomer: ^{1}H NMR (500 MHz, $CDCl_3$, HH-COSY, HSQC, HMBC): δ 5.55 (s, 1H, C*H*Ph), 4.89 (d, *J* = 11.4 Hz, 1H, C*H*H Bn), 4.29 (dd, *J* = 10.5, 5.0 Hz, 1H, H-6), 3.79 (t, *J* = 10.3 Hz, 1H, H-6), 3.45 (t, *J* = 8.3 Hz, 1H, H-2), 3.40 (dt, *J* = 9.5, 4.8 Hz, 1H, H-5); ^{13}C-APT NMR (126 MHz, $CDCl_3$, HSQC, HMBC): δ 101.2 (CHPh), 96.8 (C-1), 82.4 (C-2), 81.7 (C-4), 81.5 (C-3), 75.8 (C_q), 75.3 (CH_2 Bn), 75.0 (CH_2 Bn), 69.1 (C-6), 66.1 (C-5), 42.9 (CH Adamantyl), 35.9 (CH_2 Adamantyl), 31.3 (CH_2 Adamantyl); ^{13}C-GATED NMR (126 MHz, $CDCl_3$): δ 96.8 ($J_{C1,H1}$ = 158.6 Hz, C-1 β).

Competition Experiment Data

<u>Data - 2-Monofluoroethanol</u>

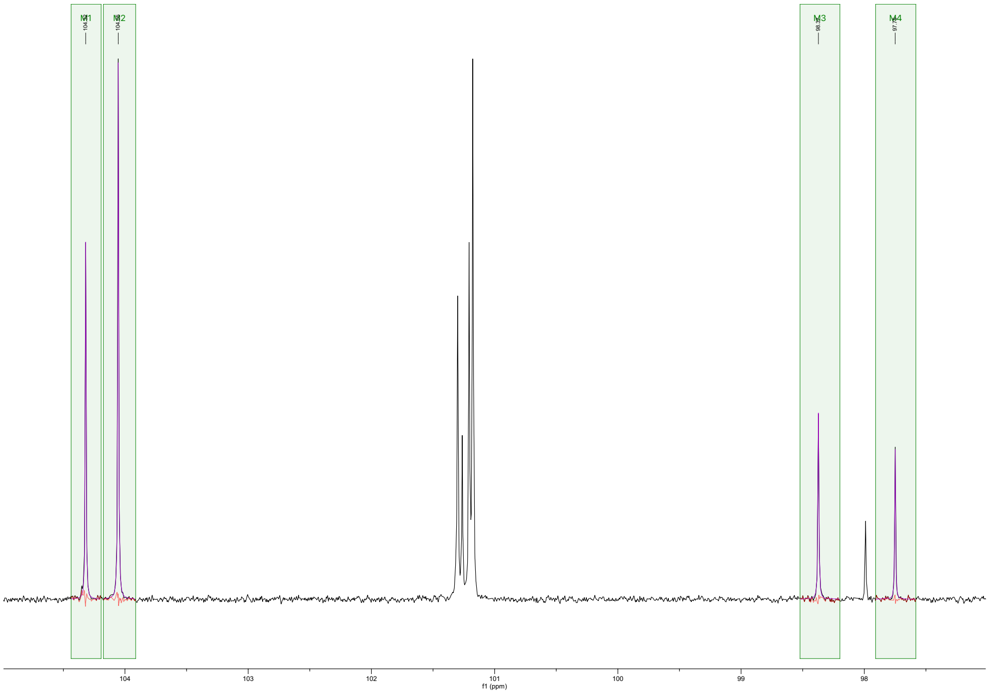

Supplementary Figure 1. ^{13}C-NMR (inverse-gated decoupling, D_1 = 15 s), 214 MHz, $CDCl_3$ of crude product mixture for acceptor competition between EtOH and MFE.

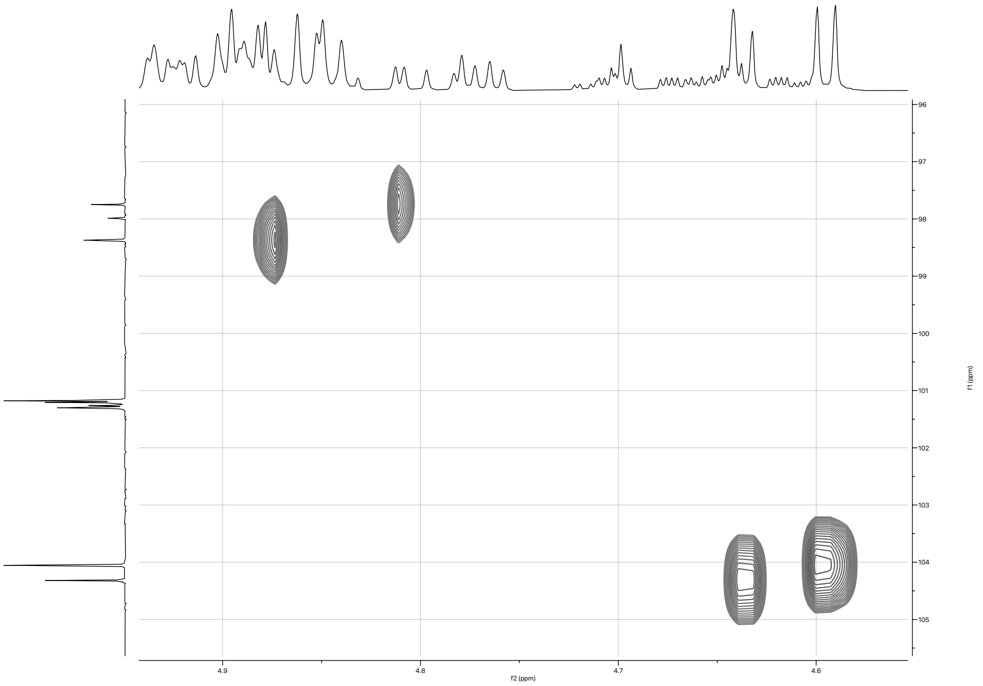

Supplementary Figure 2. Quantitative 2D HSQC-NMR of region of interest for the competition

Data - 2,2-Difluoroethanol

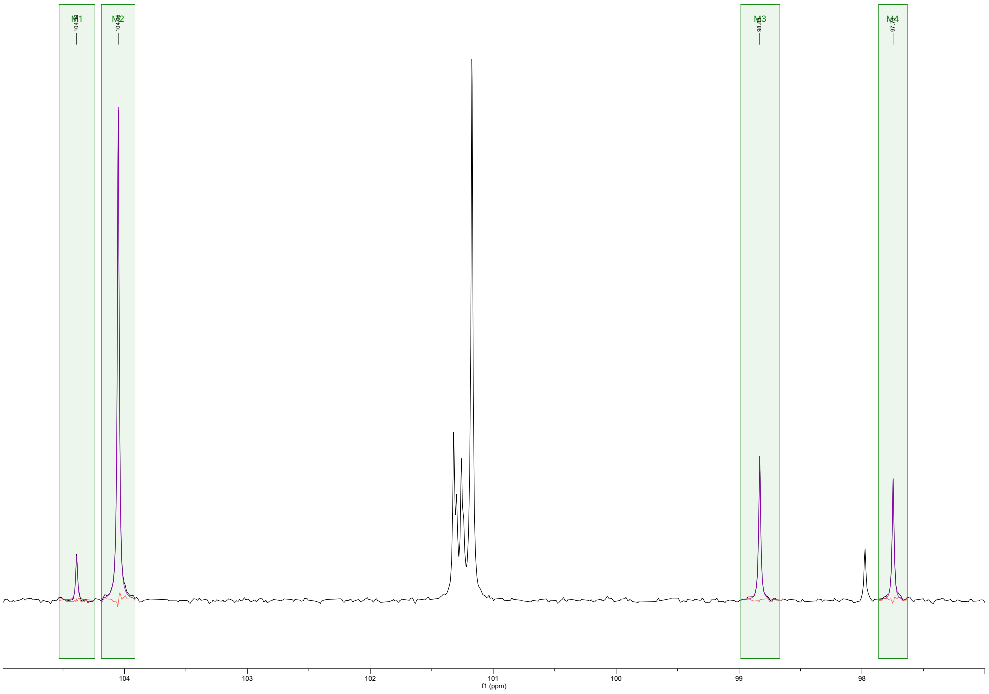

Supplementary Figure 3. ^{13}C-NMR (inverse-gated decoupling, D_1 = 15 s), 214 MHz, $CDCl_3$ of crude product mixture for acceptor competition between EtOH and DFE.

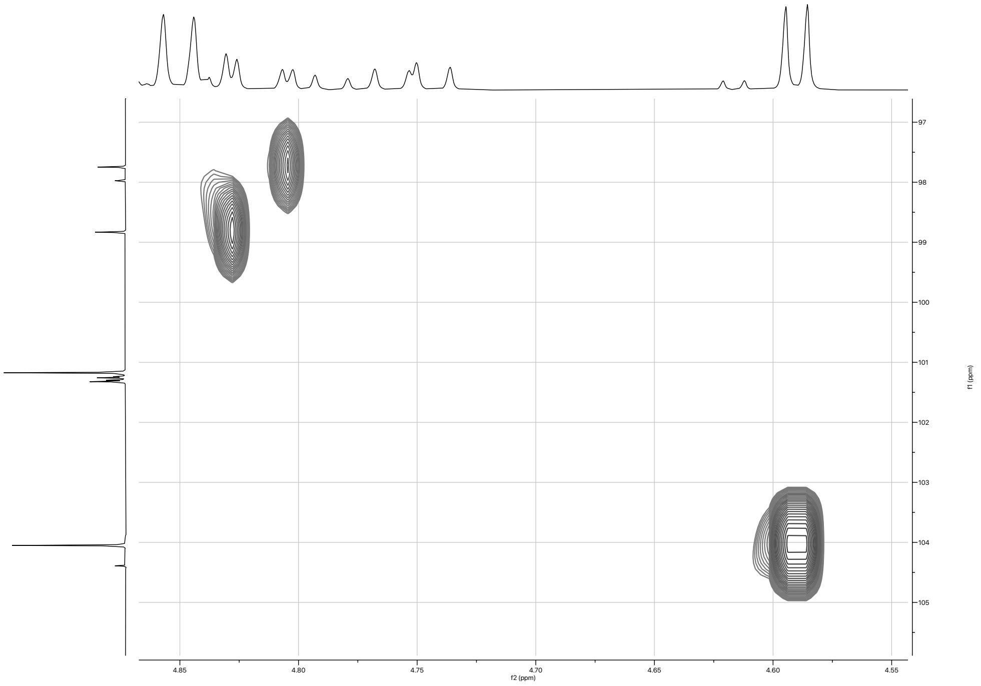

Supplementary Figure 4. Quantitative 2D HSQC-NMR of region of interest for the competition

Data - 2,2,2-Trifluoroethanol

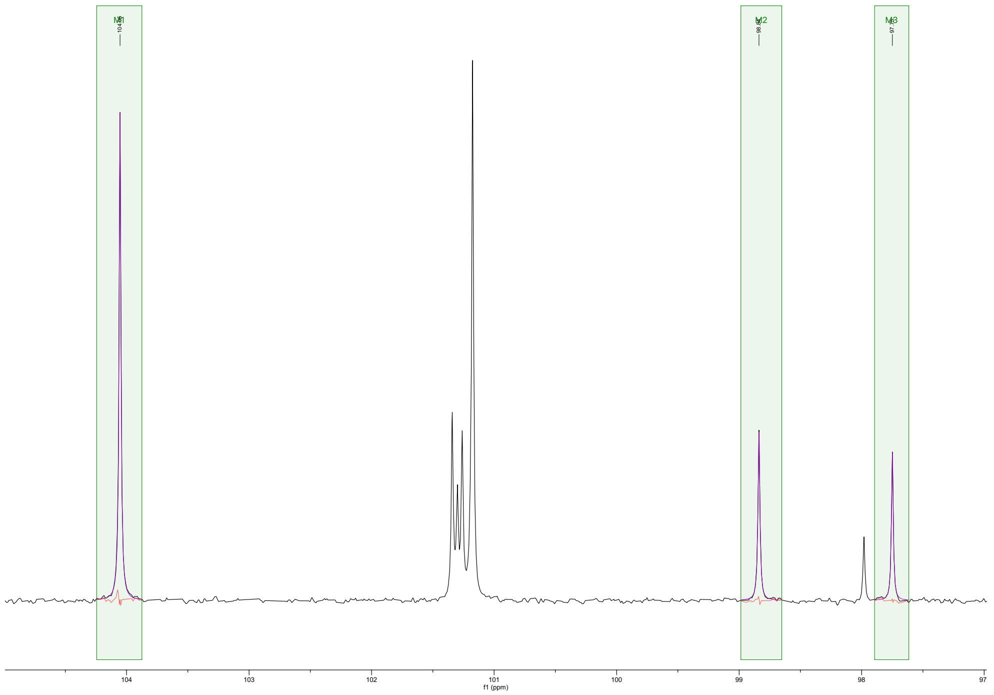

Supplementary Figure 5. ^{13}C-NMR (inverse-gated decoupling, D_1 = 15 s), 214 MHz, $CDCl_3$ of crude product mixture for acceptor competition between EtOH and TFE.

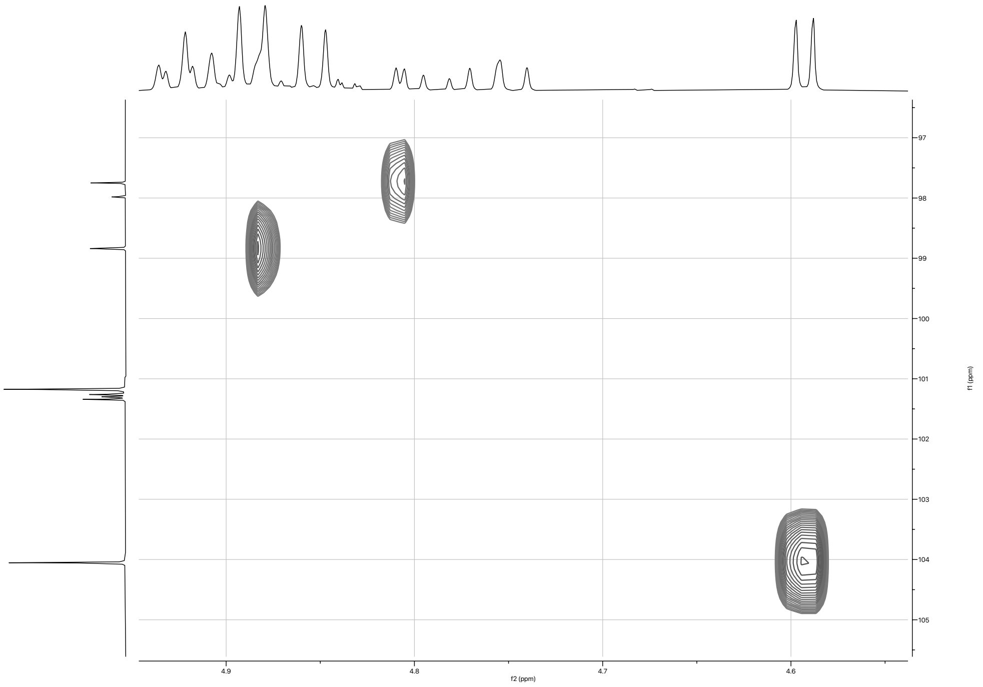

Supplementary Figure 6. Quantitative 2D HSQC-NMR of region of interest for the competition

Data - *iso*-Propanol

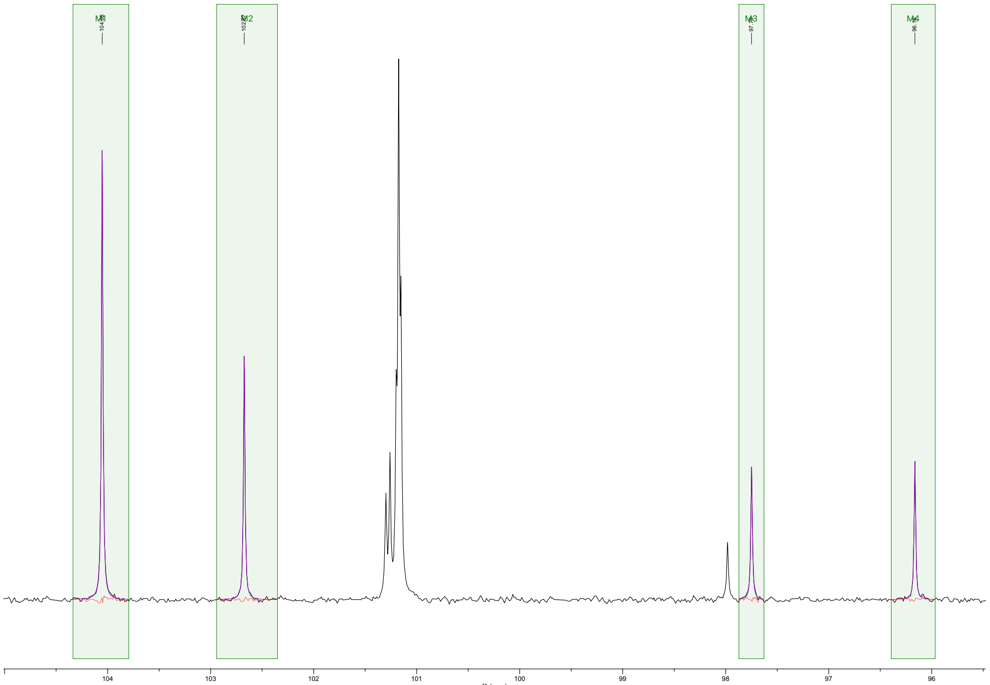

Supplementary Figure 7. ^{13}C-NMR (inverse-gated decoupling, D_1 = 15 s), 214 MHz, $CDCl_3$ of crude product mixture for acceptor competition between EtOH and iPrOH.

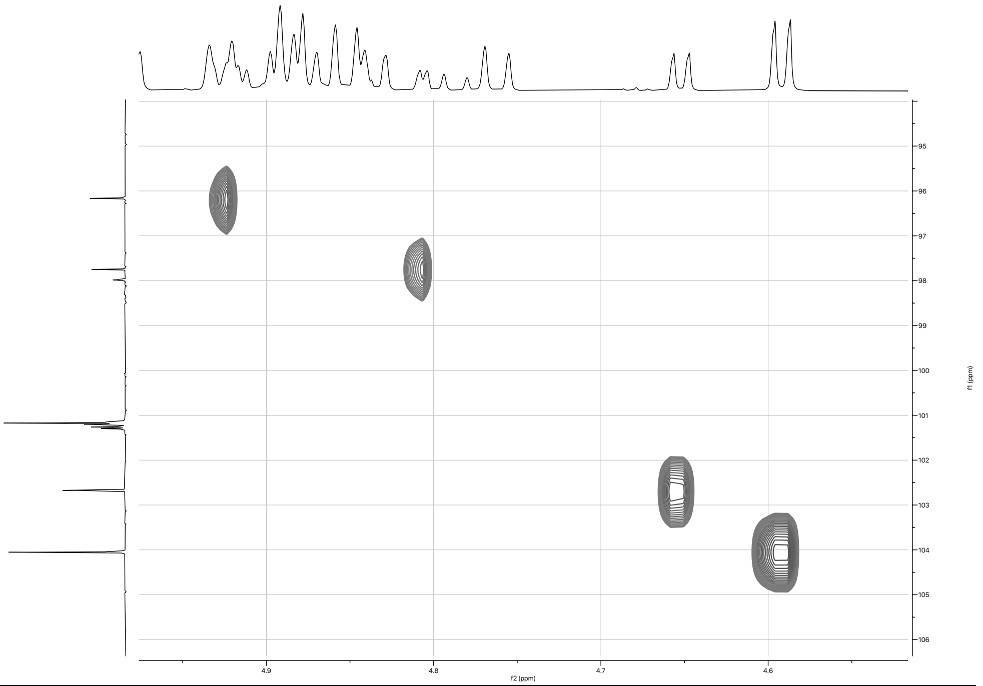

Supplementary Figure 8. Quantitative 2D HSQC-NMR of region of interest for the competition

Data - *cyclo*-Hexanol

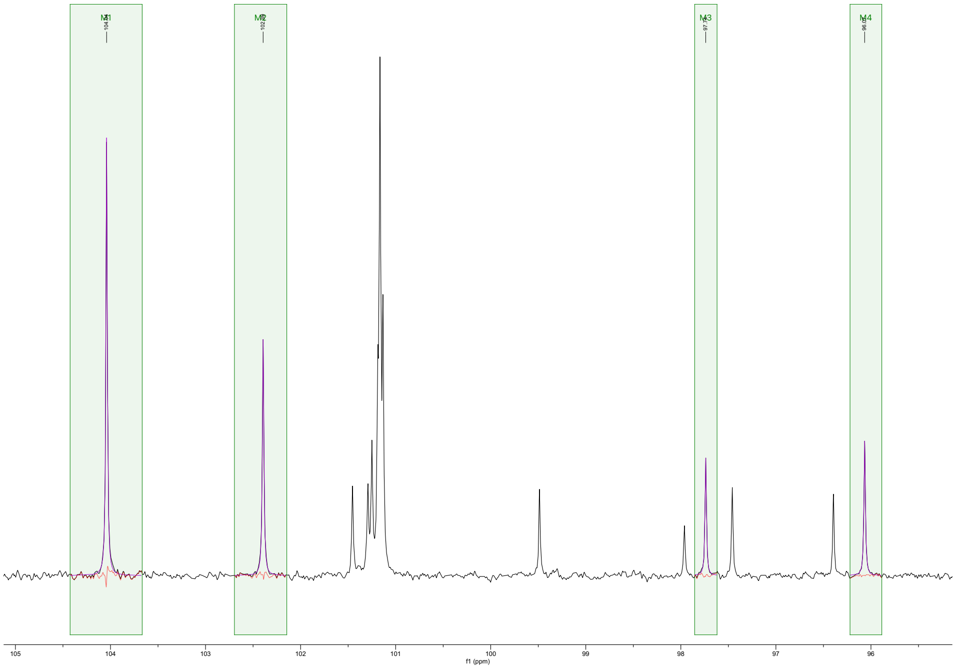

Supplementary Figure 9. ^{13}C-NMR (inverse-gated decoupling, D_1 = 15 s), 214 MHz, $CDCl_3$ of crude product mixture for acceptor competition between EtOH and CyOH.

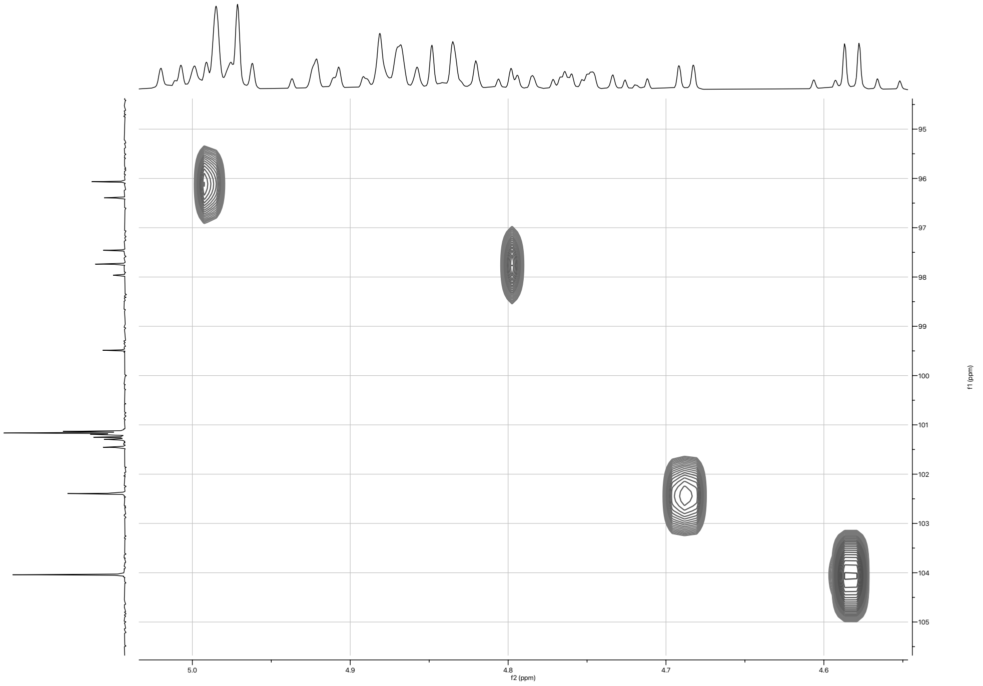

Supplementary Figure 10. Quantitative 2D HSQC-NMR of region of interest for the competition

Data - *tert*-Butanol

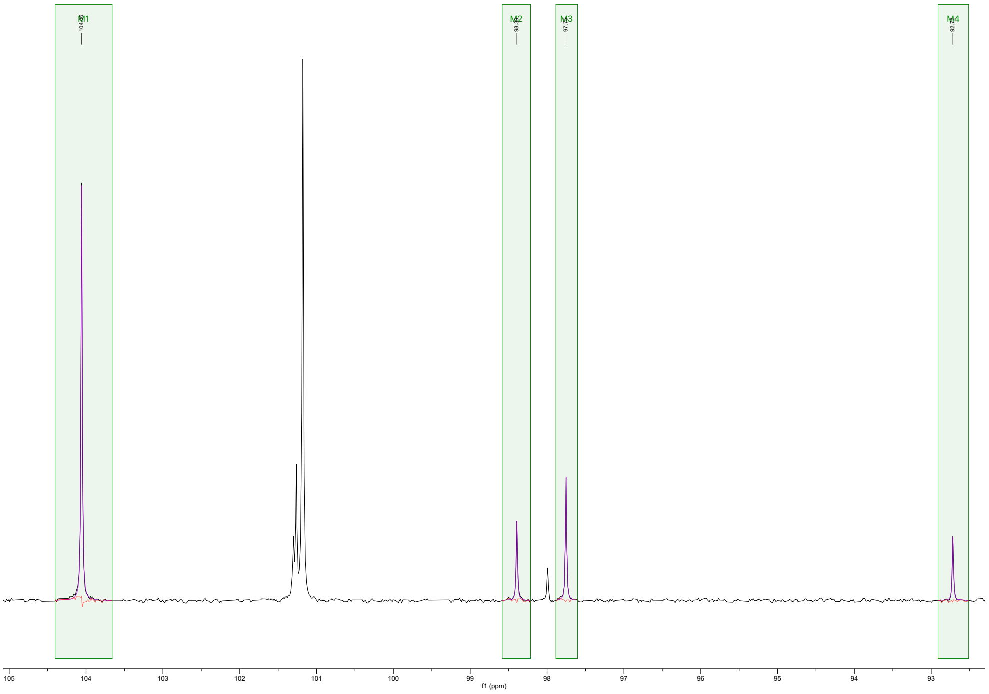

Supplementary Figure 11. ^{13}C-NMR (inverse-gated decoupling, D_1 = 15 s), 214 MHz, $CDCl_3$ of crude product mixture for acceptor competition between EtOH and tBuOH.

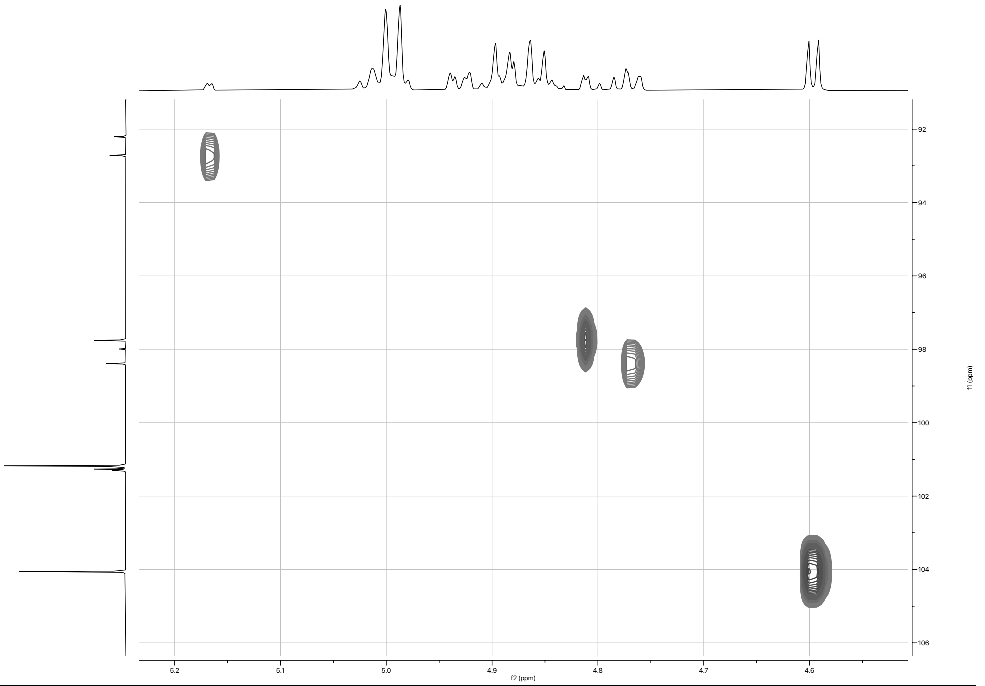

Supplementary Figure 12. Quantitative 2D HSQC-NMR of region of interest for the competition

Data - 1-Adamantanol

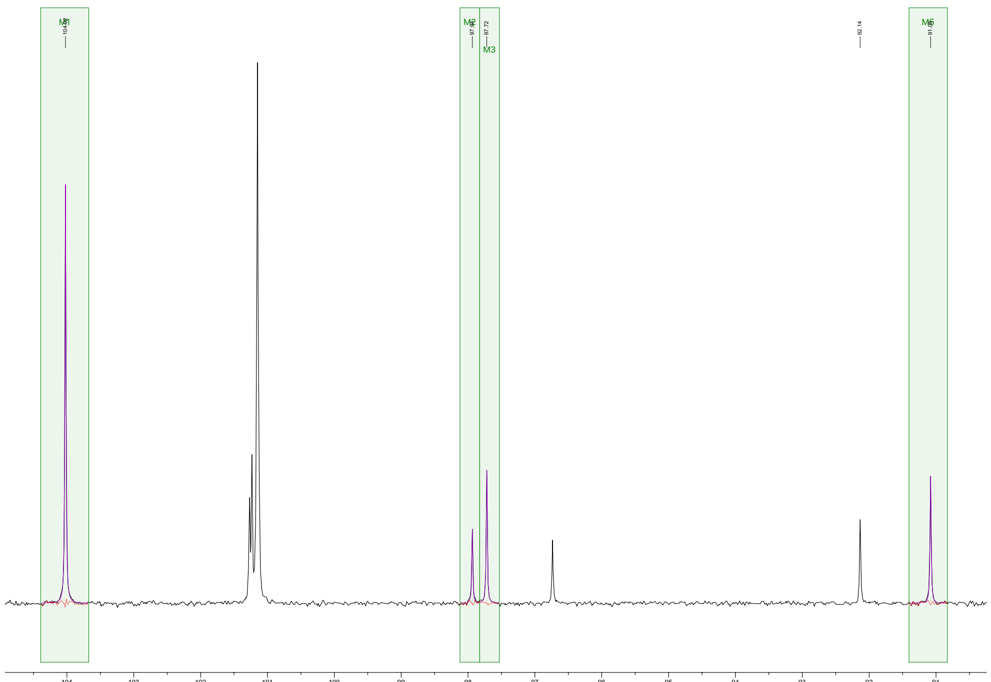

Supplementary Figure 13. ^{13}C-NMR (inverse-gated decoupling, D_1 = 15 s), 214 MHz, $CDCl_3$ of crude product mixture for acceptor competition between EtOH and AdmOH.

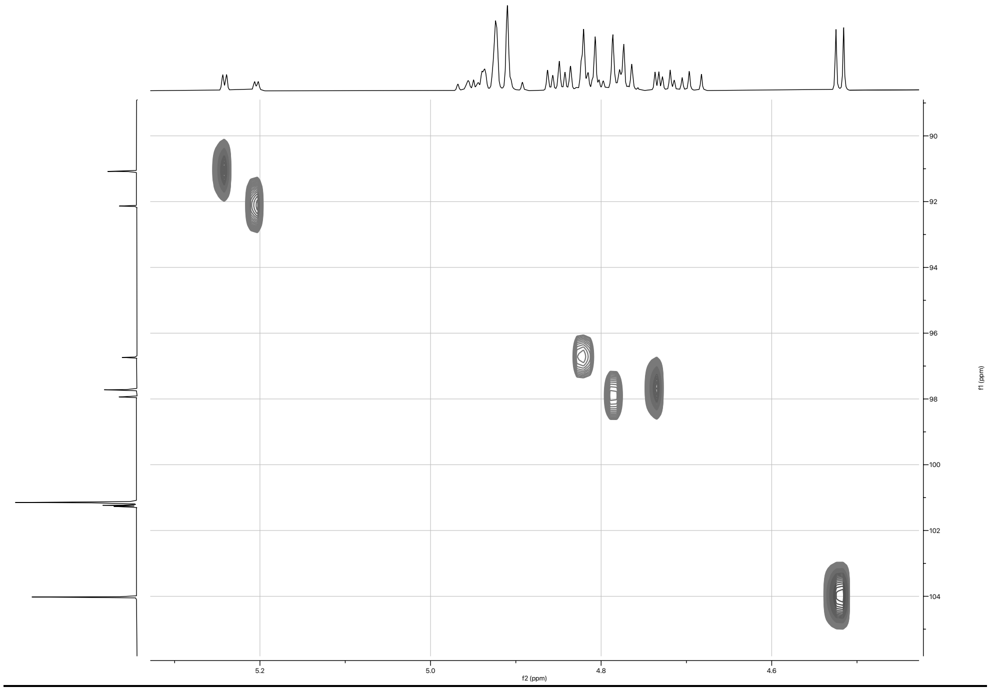

Supplementary Figure 14. Quantitative 2D HSQC-NMR of region of interest for the competition

Kinetic Isotope Effect (KIE) Data

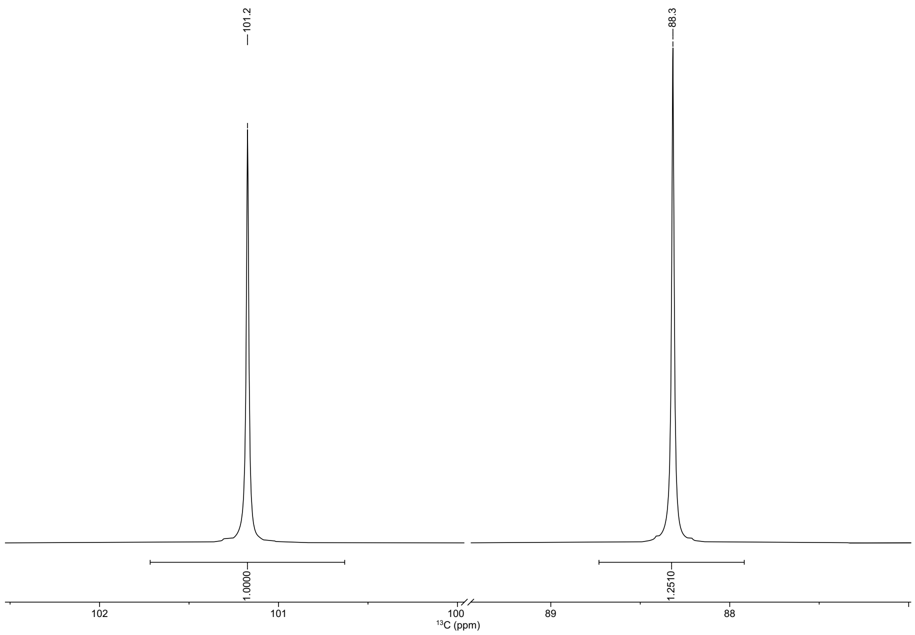

Supplementary Figure 15. ^{13}C-NMR (inverse-gated decoupling, D_1 = 15 s), 214 MHz, $CDCl_3$ of compound **S7**, Ph_2SO, TTBP and internal standard, 4,4,5,5-tetramethyl-2-(1-naphthyl)-1,3-dioxolane, zoomed in on the peaks of interest. In this case, R_0 = 1.2510.

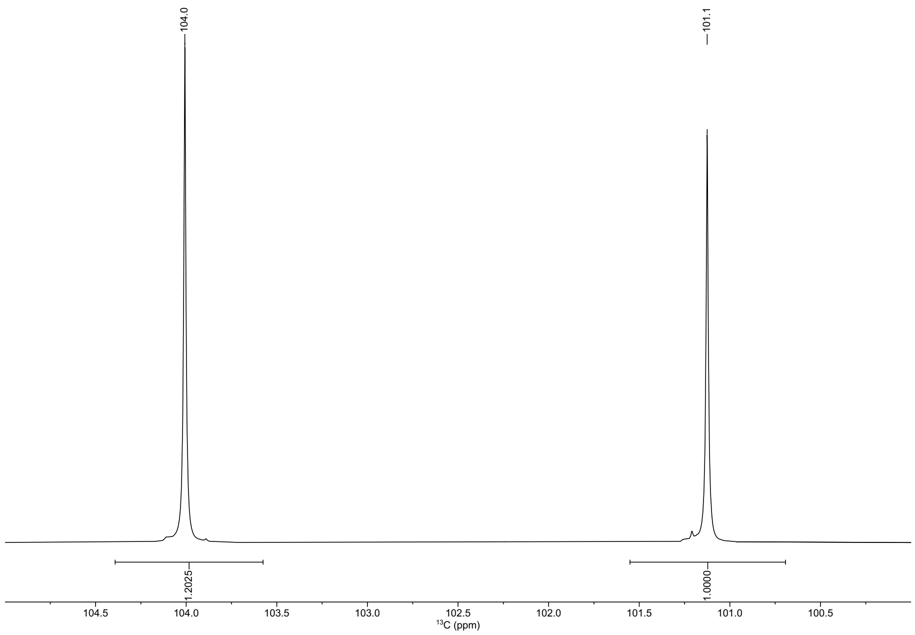

Supplementary Figure 16. ^{13}C-NMR (inverse-gated decoupling, D_1 = 15 s), 214 MHz, $CDCl_3$ of purified β-*O*-ethyl glucoside **S16-β**, zoomed in on the peaks of interest. In this case, R_p = 1.2025.

Supplementary Table 1. Integral data and KIE calculations for the glycosylation reaction towards the formation of α-*O*-ethyl glucosides.

Entry	R_0	R_p	*F*	KIE @ 233K	KIE @ 298 K	Average KIE
1	1.2510	1.2130	0.39	1.0405	1.032	
2	1.2510	1.2130	0.38	1.0401	1.031	1.031 ± 0.001
3	1.2510	1.2156	0.40	1.0380	1.030	

Supplementary Table 2. Integral data and KIE calculations for the glycosylation reaction towards the formation of β-*O*-ethyl glucosides.

Entry	R_0	R_p	*F*	KIE @ 233K	KIE @ 298 K	Average KIE
1	1.2510	1.2111	0.20	1.0369	1.029	
2	1.2510	1.2077	0.16	1.0391	1.030	1.031 ± 0.003
3	1.2510	1.2036	0.18	1.0435	1.034	

Supplementary Table 3. Integral data and KIE calculations for the glycosylation reaction towards the formation of α-*O*-monofluoroethyl glucosides.

Entry	R_0	R_p	*F*	KIE @ 233K	KIE @ 298 K	Average KIE
1	1.2510	1.2296	0.33	1.0214	1.017	
2	1.2510	1.2301	0.38	1.0218	1.017	1.017 ± 0.001
3	1.2510	1.2278	0.37	1.0240	1.019	

Supplementary Table 4. Integral data and KIE calculations for the glycosylation reaction towards the formation of β-*O*-monofluoroethyl glucosides.

Entry	R_0	R_p	*F*	KIE @ 233K	KIE @ 298 K	Average KIE
1	1.2510	1.2176	0.41	1.0361	1.028	
2	1.2510	1.2157	0.38	1.0372	1.029	1.028 ± 0.001
3	1.2510	1.2193	0.42	1.0345	1.027	

Supplementary Table 5. Integral data and KIE calculations for the glycosylation reaction towards the formation of α-*O*-difluoroethyl glucosides.

Entry	R_0	R_p	*F*	KIE @ 233K	KIE @ 298 K	Average KIE
1	1.2510	1.2373	0.65	1.0196	1.015	
2	1.2510	1.2393	0.67	1.0173	1.013	1.014 ± 0.001
3	1.2510	1.2381	0.62	1.0176	1.014	

Supplementary Table 6. Integral data and KIE calculations for the glycosylation reaction towards the formation of β-*O*-difluoroethyl glucosides.

Entry	R_0	R_p	*F*	KIE @ 233K	KIE @ 298 K	Average KIE
1	1.2510	1.2146	0.18	1.0331	1.026	
2	1.2510	1.2135	0.19	1.0344	1.027	1.026 ± 0.001
3	1.2510	1.2151	0.21	1.0334	1.026	

Supplementary Table 7. Integral data and KIE calculations for the glycosylation reaction towards the formation of α-*O*-trifluoroethyl glucosides.

Entry	R_0	R_p	*F*	KIE @ 233K	KIE @ 298 K	Average KIE
1	1.2510	1.2448	0.75	1.0107	1.008	
2	1.2510	1.2446	0.72	1.0104	1.008	1.008 ± 0.001
3	1.2510	1.2446	0.78	1.0120	1.009	

Supplementary Table 8. Integral data and KIE calculations for the glycosylation reaction towards the formation of α-*O*-isopropyl glucosides.

Entry	R_0	R_p	*F*	KIE @ 233K	KIE @ 298 K	Average KIE
1	1.2344	1.2099	0.35	1.0254	1.020	
2	1.2344	1.2076	0.35	1.0278	1.022	1.020 ± 0.002
3	1.2344	1.2121	0.36	1.0232	1.018	

Supplementary Table 9. Integral data and KIE calculations for the glycosylation reaction towards the formation of β-*O*-isopropyl glucosides.

Entry	R_0	R_p	*F*	KIE @ 233K	KIE @ 298 K	Average KIE
1	1.2344	1.1980	0.41	1.0399	1.031	
2	1.2344	1.1961	0.43	1.0430	1.033	1.032 ± 0.001
3	1.2344	1.1975	0.40	1.0402	1.031	

Supplementary Table 10. Integral data and KIE calculations for the glycosylation reaction towards the formation of α-*O*-cyclohexyl glucosides.

Entry	R_0	R_p	*F*	KIE @ 233K	KIE @ 298 K	Average KIE
1	1.2344	1.2134	0.31	1.0210	1.016	
2	1.2344	1.2048	0.32	1.0299	1.023	1.020 ± 0.004
3	1.2344	1.2072	0.29	1.0269	1.021	

Supplementary Table 11. Integral data and KIE calculations for the glycosylation reaction towards the formation of β-*O*-cyclohexyl glucosides.

Entry	R_0	R_p	*F*	KIE @ 233K	KIE @ 298 K	Average KIE
1	1.2344	1.1903	0.26	1.0432	1.034	
2	1.2344	1.1963	0.24	1.0366	1.029	1.030 ± 0.003
3	1.2344	1.1982	0.26	1.0353	1.027	

Acceptor Concentration Data

Data - Ethanol

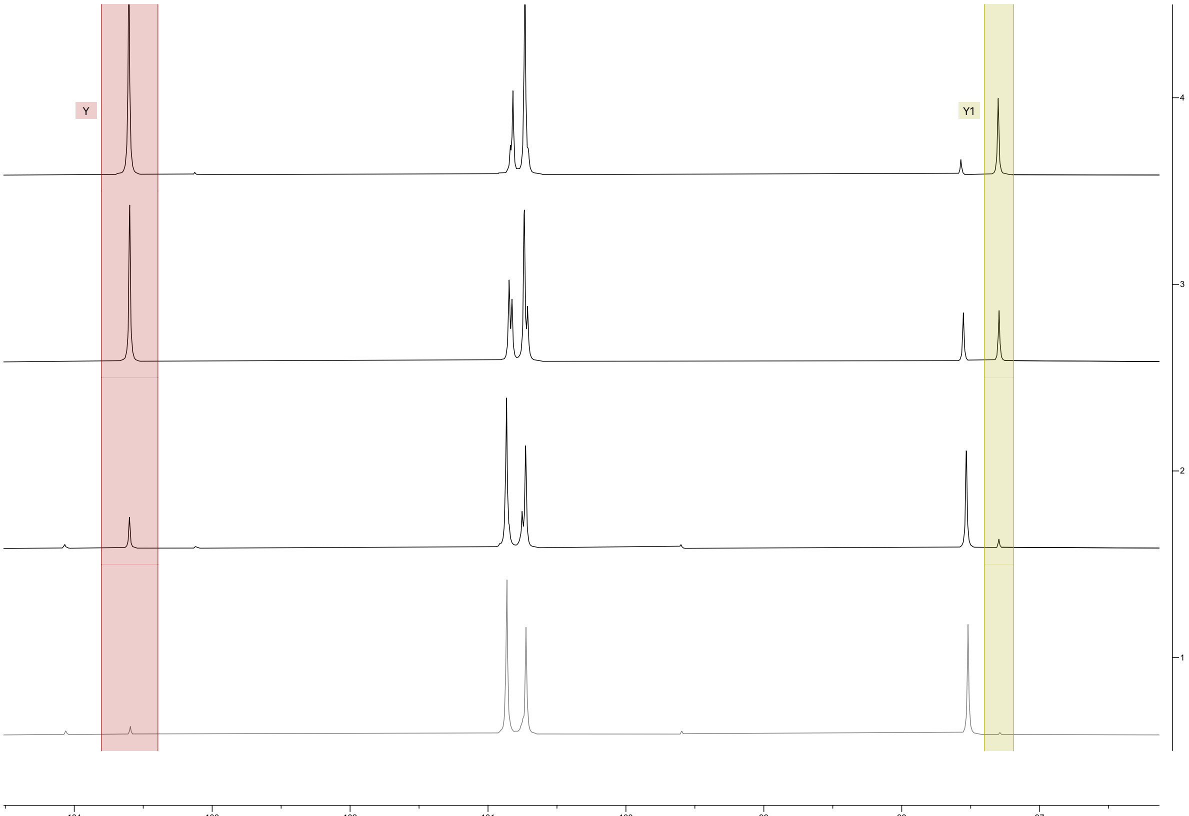

Supplementary Figure 17. Stacked ^{13}C-NMR (inverse-gated decoupling, D_1 = 15 s), 214 MHz, $CDCl_3$ of crude *O*-ethyl glucoside **S8**, zoomed in on the peaks of interest. Acceptor amounts: 1 = 4.0 µL; 2 = 8.0 µL; 3 = 16.0 µL; 4 = 32.0 µL.

Supplementary Table 12. Acceptor concentration data and the fitted kinetics parameter ratios for the glycosylation reaction towards α/β-*O*-ethyl glucosides.

Entry	V_A [µL]	m_A [mg]	n_A [mmol]	C_A [M]	$1/C_A$ [M^{-1}]	α/β [-]	$k_{SN1\alpha}/k_{SN2\beta}$ [$\cdot 10^{-3}$ M]	$k_{SN2\alpha}/k_{SN2\beta}$ [M]	$k_{SN1\alpha}/k_{SN2\alpha}$ [$\cdot 10^{-3}$]
**1**	4.0	3.156	0.069	0.017	58.388	0.348	0.235	0.335	0.702
**2**	8.0	6.312	0.137	0.034	29.194	0.341			
**3**	16.0	12.624	0.274	0.069	14.597	0.340			
**4**	32.0	25.248	0.548	0.137	7.298	0.335			

Data - 2-Fluoroethanol

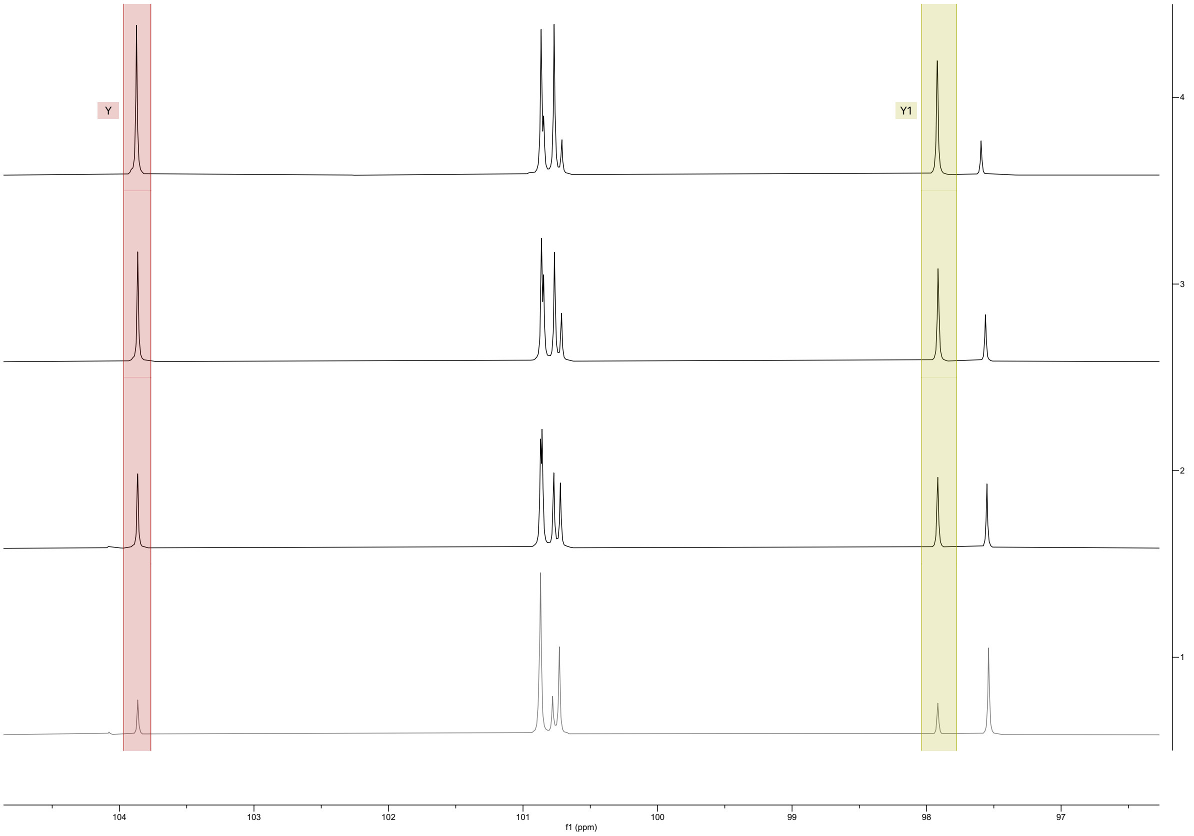

Supplementary Figure 18. Stacked ^{13}C-NMR (inverse-gated decoupling, D_1 = 15 s), 214 MHz, $CDCl_3$ of crude *O*-monofluoroethyl glucoside **S9**, zoomed in on the peaks of interest. Acceptor amounts: 1 = 4.0 µL; 2 = 8.0 µL; 3 = 16.0 µL; 4 = 32.0 µL.

Supplementary Table 13. Acceptor concentration data and the fitted kinetics parameter ratios for the glycosylation reaction towards α/β-*O*-monofluoroethyl glucosides.

Entry	V_A [µL]	m_A [mg]	n_A [mmol]	C_A [M]	$1/C_A$ [M^{-1}]	α/β [-]	$k_{SN1\alpha}/k_{SN2\beta}$ [$\cdot 10^{-3}$ M]	$k_{SN2\alpha}/k_{SN2\beta}$ [M]	$k_{SN1\alpha}/k_{SN2\alpha}$ [$\cdot 10^{-3}$]
**1**	4.0	4.416	0.069	0.017	58.024	1.225	5.609	0.913	6.145
**2**	8.0	8.832	0.138	0.034	29.012	1.105			
**3**	16.0	17.664	0.276	0.069	14.506	0.996			
**4**	32.0	35.328	0.551	0.138	7.253	0.935			

Data - 2,2-Difluoroethanol

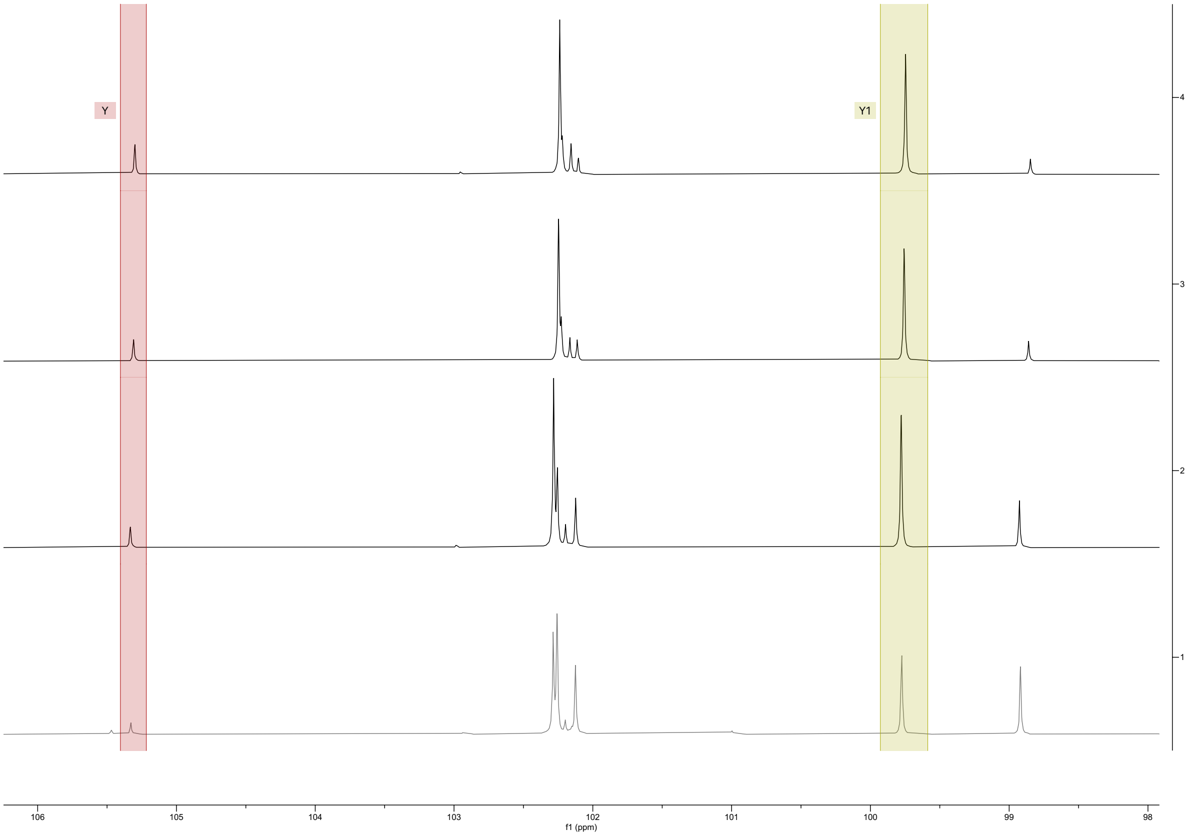

Supplementary Figure 19. Stacked ^{13}C-NMR (inverse-gated decoupling, D_1 = 15 s), 214 MHz, $CDCl_3$ of crude *O*-difluoroethyl glucoside **S10**, zoomed in on the peaks of interest. Acceptor amounts: 1 = 4.0 µL; 2 = 8.0 µL; 3 = 16.0 µL; 4 = 32.0 µL.

Supplementary Table 14. Acceptor concentration data and the fitted kinetics parameter ratios for the glycosylation reaction towards α/β-*O*-difluoroethyl glucosides.

**Entry**	V_A [µL]	m_A [mg]	n_A [mmol]	C_A [M]	$1/C_A$ [M^{-1}]	α/β [-]	$k_{SN1\alpha}/k_{SN2\beta}$ [$\cdot 10^{-3}$ M]	$k_{SN2\alpha}/k_{SN2\beta}$ [M]	$k_{SN1\alpha}/k_{SN2\alpha}$ [$\cdot 10^{-3}$]
**1**	4.0	5.184	0.064	0.016	62.512	8.648	55.227	5.297	10.426
**2**	8.0	10.368	0.128	0.032	31.256	7.263			
**3**	16.0	20.736	0.256	0.064	15.628	6.148			
**4**	32.0	41.472	0.512	0.128	7.814	5.602			

Data - *iso*-Propanol

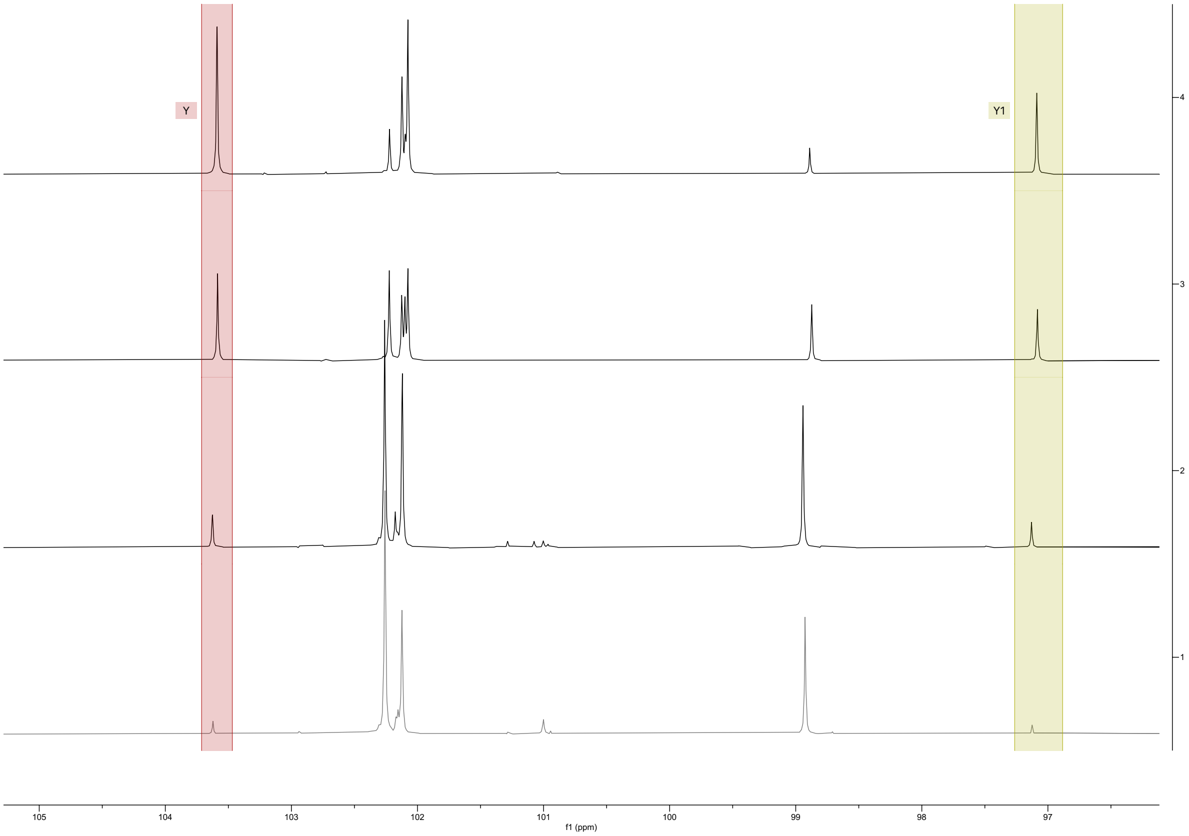

Supplementary Figure 20. Stacked ^{13}C-NMR (inverse-gated decoupling, D_1 = 15 s), 214 MHz, $CDCl_3$ of crude *O*-isopropyl glucoside **S12**, zoomed in on the peaks of interest. Acceptor amounts: 1 = 4.0 µL; 2 = 8.0 µL; 3 = 16.0 µL; 4 = 32.0 µL.

Supplementary Table 15. Acceptor concentration data and the fitted kinetics parameter ratios for the glycosylation reaction towards α/β-*O*-isopropyl glucosides.

**Entry**	**V_A** [µL]	**m_A** [mg]	**n_A** [mmol]	**C_A** [M]	**$1/C_A$** [M^{-1}]	**α/β** [-]	**$k_{SN1\alpha}/k_{SN2\beta}$** [$\cdot 10^{-3}$ M]	**$k_{SN2\alpha}/k_{SN2\beta}$** [M]	**$k_{SN1\alpha}/k_{SN2\alpha}$** [$\cdot 10^{-3}$]
**1**	4.0	3.144	0.052	0.013	76.463	0.771	2.896	0.547	5.291
**2**	8.0	6.288	0.105	0.026	38.232	0.651			
**3**	16.0	12.576	0.209	0.052	19.116	0.613			
**4**	32.0	25.152	0.419	0.105	9.558	0.571			

Data - *cyclo*-Hexanol

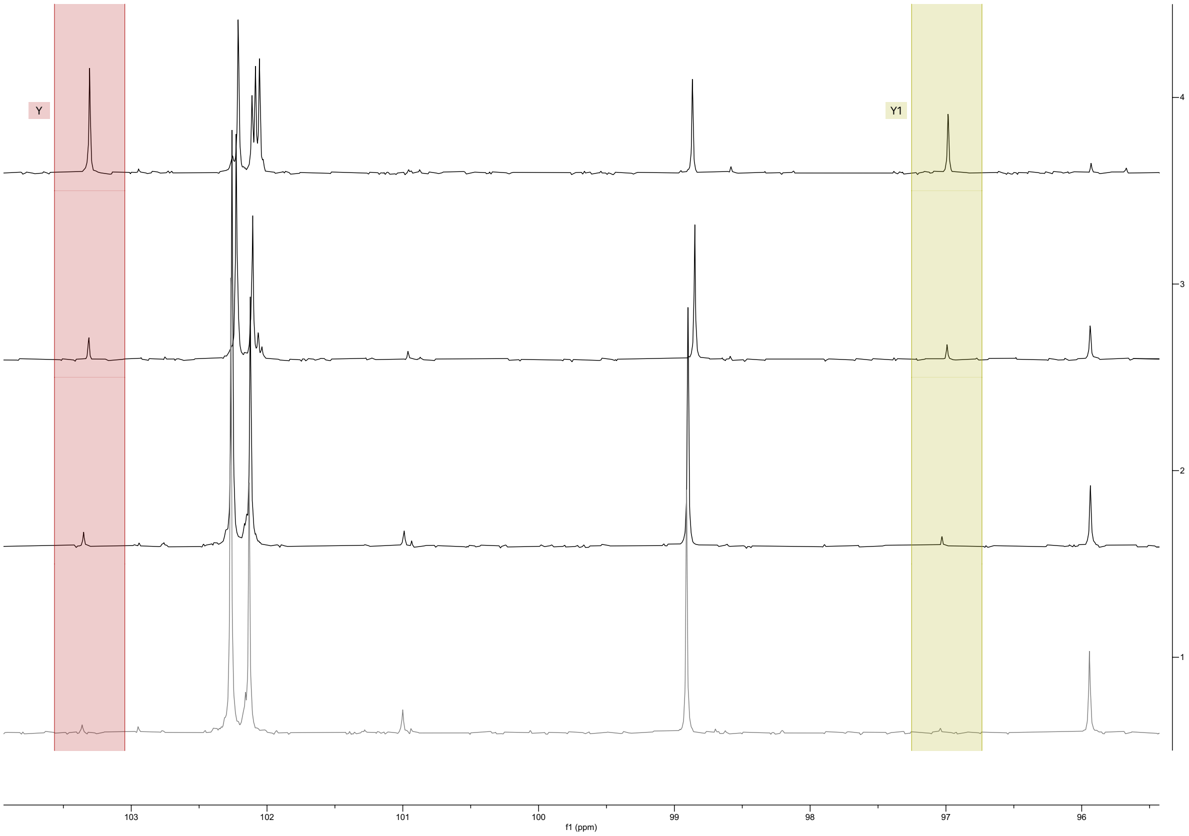

Supplementary Figure 21. Stacked ^{13}C-NMR (inverse-gated decoupling, D_1 = 15 s), 214 MHz, $CDCl_3$ of crude *O*-cyclohexyl glucoside **S13**, zoomed in on the peaks of interest. Acceptor amounts: 1 = 4.0 μL; 2 = 8.0 μL; 3 = 16.0 μL; 4 = 32.0 μL.

Supplementary Table 16. Acceptor concentration data and the fitted kinetics parameter ratios for the glycosylation reaction towards α/β-*O*-cyclohexyl glucosides.

Entry	V_A [μL]	m_A [mg]	n_A [mmol]	C_A [M]	$1/C_A$ [M^{-1}]	α/β [-]	$k_{SN1\alpha}/k_{SN2\beta}$ [$\cdot 10^{-3}$ M]	$k_{SN2\alpha}/k_{SN2\beta}$ [M]	$k_{SN1\alpha}/k_{SN2\alpha}$ [$\cdot 10^{-3}$]
**1**	4.0	3.848	0.038	0.010	104.114	1.000	3.364	0.657	5.119
**2**	8.0	7.696	0.077	0.019	52.057	0.847			
**3**	16.0	15.392	0.154	0.038	26.029	0.752			
**4**	32.0	30.784	0.307	0.077	13.014	0.686			

Data - *tert*-Butanol

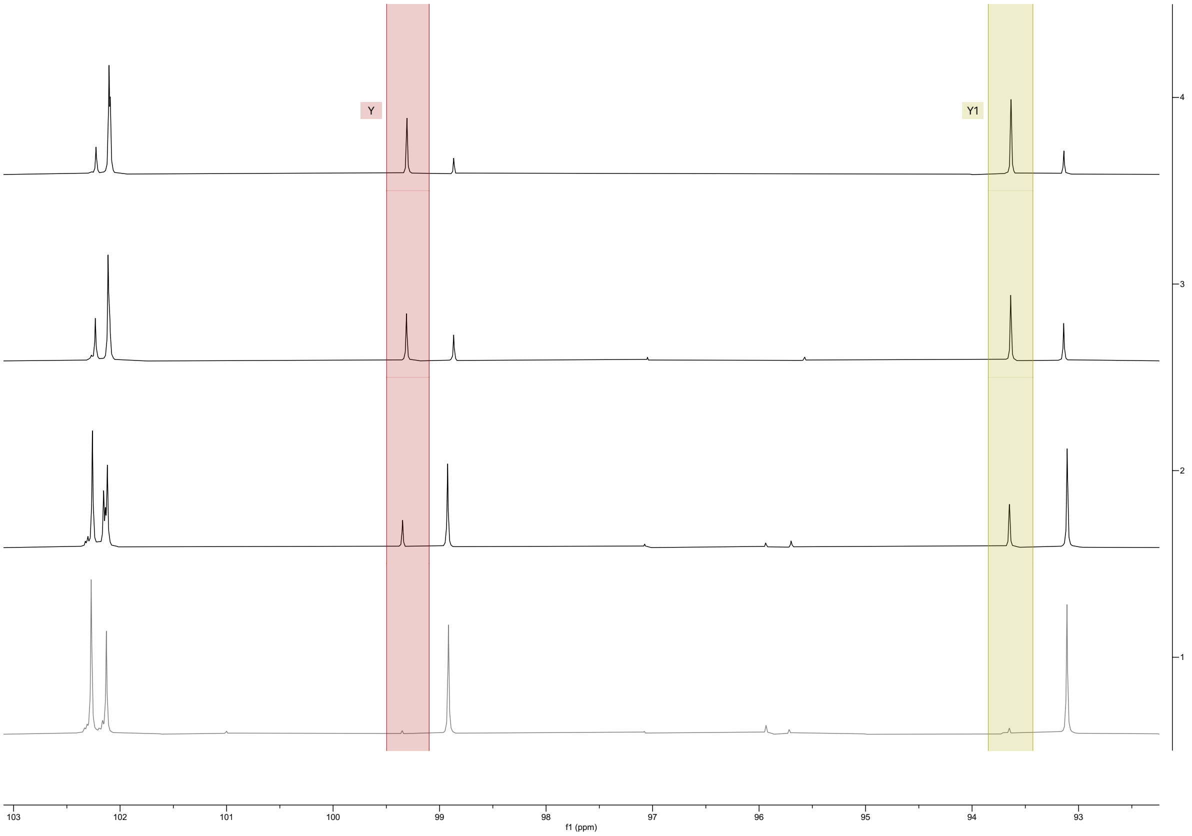

Supplementary Figure 22. Stacked ^{13}C-NMR (inverse-gated decoupling, D_1 = 15 s), 214 MHz, $CDCl_3$ of crude *O*-tertbutyl glucoside **S14**, zoomed in on the peaks of interest. Acceptor amounts: 1 = 4.0 μL; 2 = 8.0 μL; 3 = 16.0 μL; 4 = 32.0 μL.

Supplementary Table 17. Acceptor concentration data and the fitted kinetics parameter ratios for the glycosylation reaction towards α/β-*O*-tertbutyl glucosides.

Entry	V_A [μL]	m_A [mg]	n_A [mmol]	C_A [M]	$1/C_A$ [M^{-1}]	α/β [-]	$k_{SN1\alpha}/k_{SN2\beta}$ [$\cdot 10^{-3}$ M]	$k_{SN2\alpha}/k_{SN2\beta}$ [M]	$k_{SN1\alpha}/k_{SN2\alpha}$ [$\cdot 10^{-3}$]
**1**	4.0	3.124	0.042	0.011	94.904	2.201	11.246	1.145	9.820
**2**	8.0	6.248	0.084	0.021	47.452	1.710			
**3**	16.0	12.496	0.169	0.042	23.726	1.402			
**4**	32.0	24.992	0.337	0.084	11.863	1.270			

Data - 1-Adamantanol

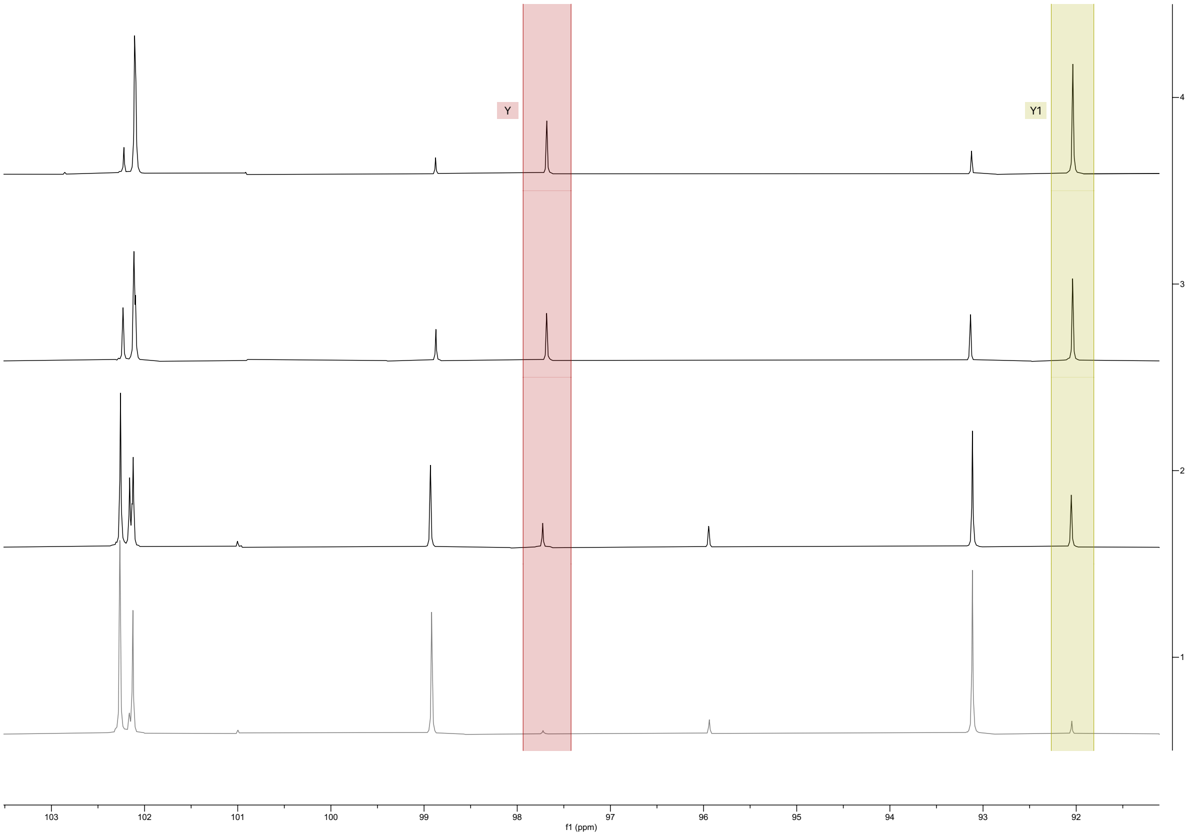

Supplementary Figure 23. Stacked ^{13}C-NMR (inverse-gated decoupling, D_1 = 15 s), 214 MHz, $CDCl_3$ of crude *O*-adamantyl glucoside **S15**, zoomed in on the peaks of interest. Acceptor amounts: 1 = 4.0 μL; 2 = 8.0 μL; 3 = 16.0 μL; 4 = 32.0 μL.

Supplementary Table 18. Acceptor concentration data and the fitted kinetics parameter ratios for the glycosylation reaction towards α/β-*O*-adamantyl glucosides.

Entry	V_A [μL]	m_A [mg]	n_A [mmol]	C_A [M]	$1/C_A$ [M^{-1}]	α/β [-]	$k_{SN1\alpha}/k_{SN2\beta}$ [$\cdot10^{-3}$ M]	$k_{SN2\alpha}/k_{SN2\beta}$ [M]	$k_{SN1\alpha}/k_{SN2\alpha}$ [$\cdot10^{-3}$]
**1**	-	4.000	0.029	0.007	136.240	3.710	17.390	1.259	13.811
**2**	-	8.000	0.059	0.015	68.120	2.277			
**3**	-	16.000	0.117	0.029	34.060	1.781			
**4**	-	32.000	0.235	0.059	17.030	1.710			

NMR Spectra of New Compounds

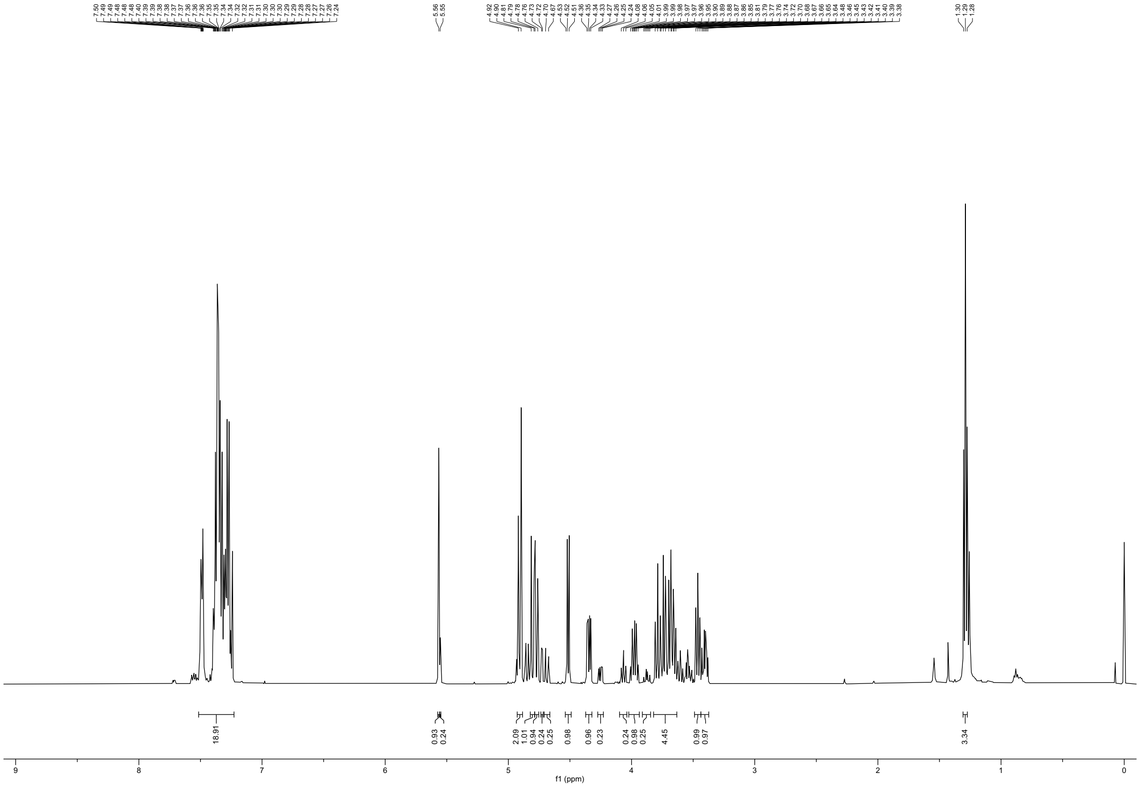

Supplementary Figure 24. ^{1}H-NMR, 500 MHz, $CDCl_3$ of compound **S8**

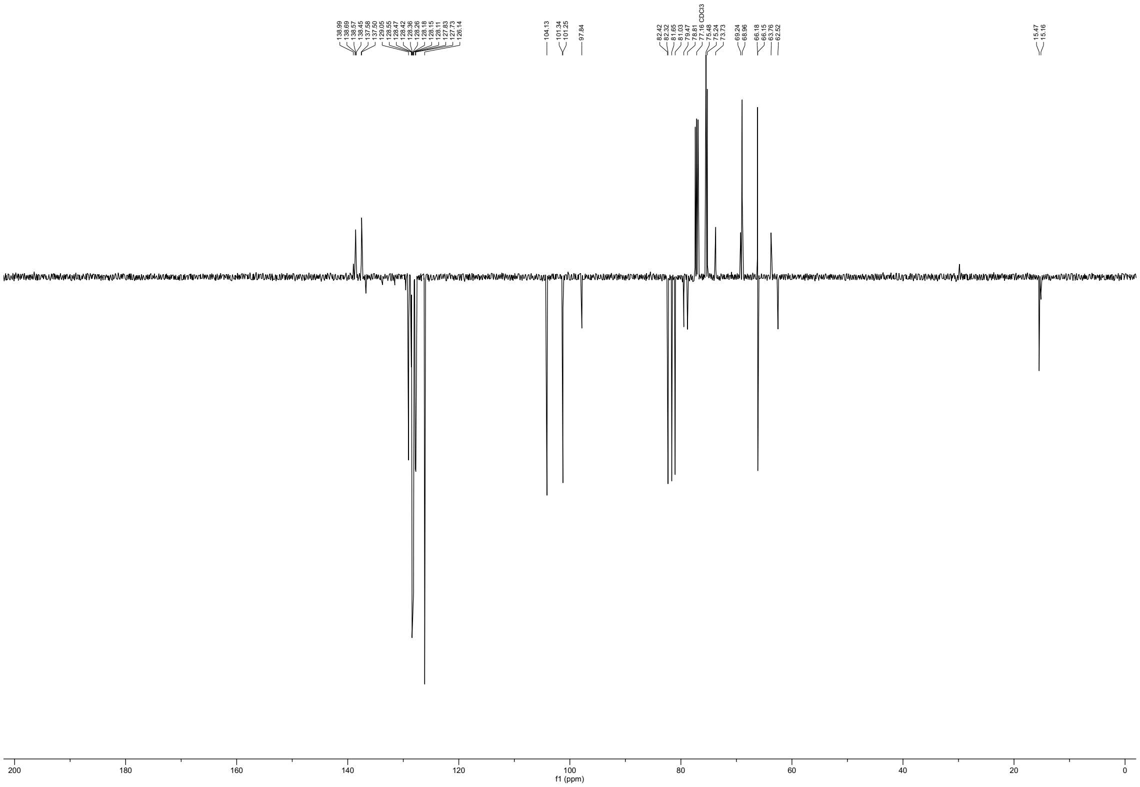

Supplementary Figure 25. ^{13}C-NMR, 126 MHz, $CDCl_3$ of compound **S8**

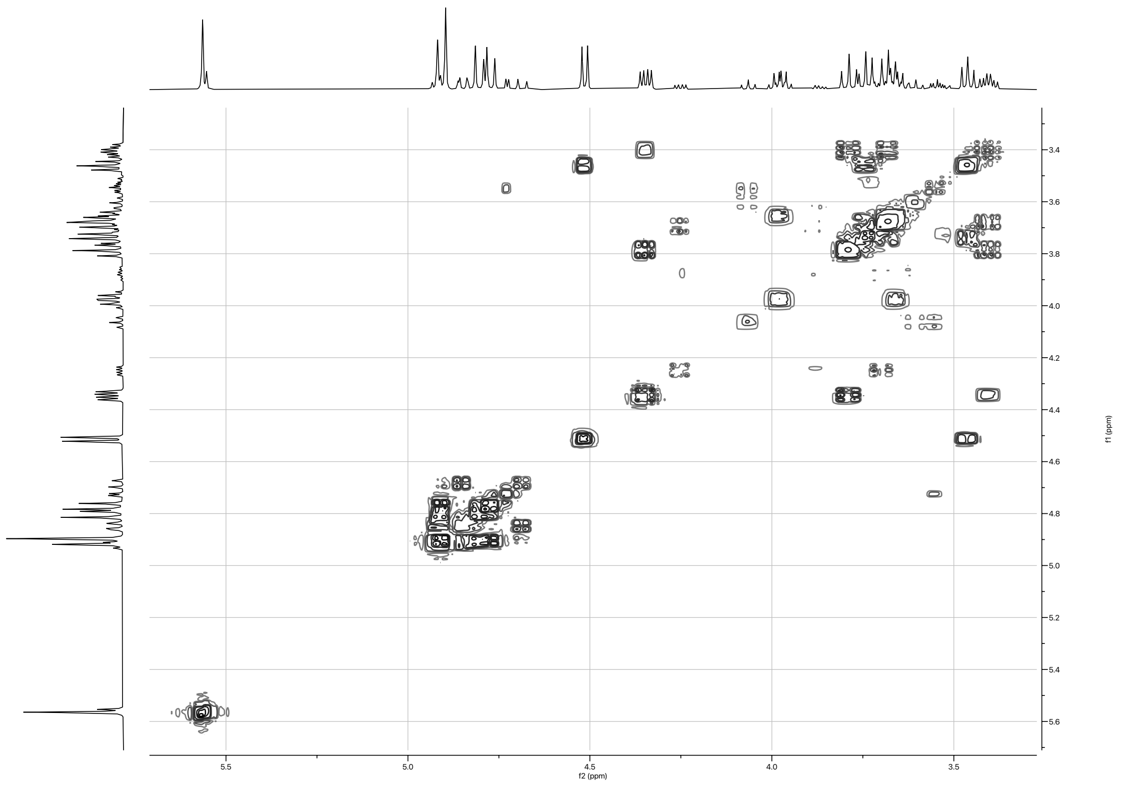

Supplementary Figure 26. HH-COSY NMR, $CDCl_3$ of compound **S8**

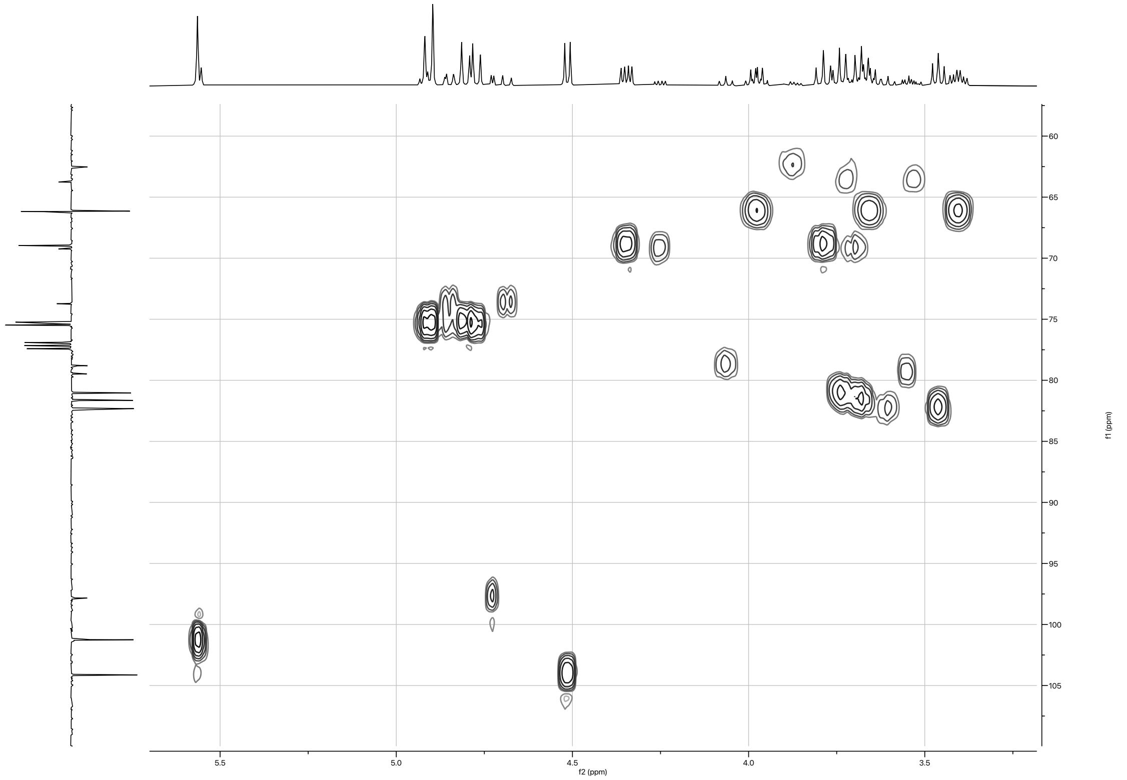

Supplementary Figure 27. HSQC NMR, $CDCl_3$ of compound **S8**

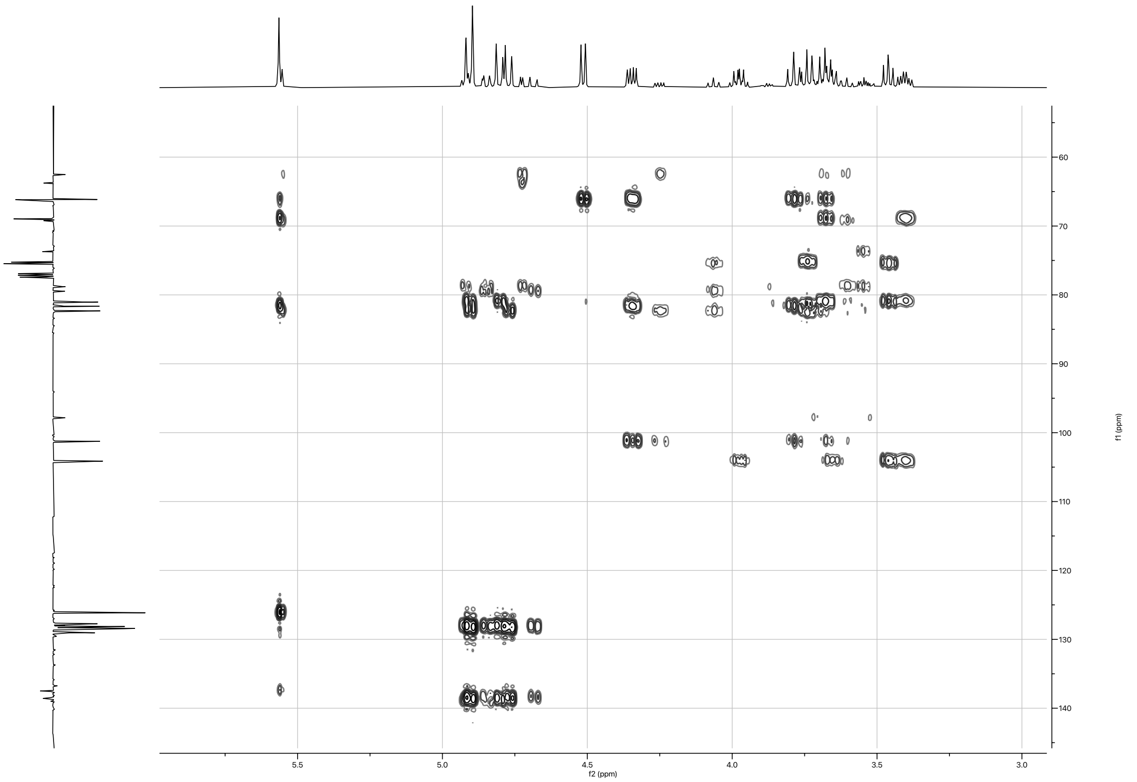

Supplementary Figure 28. HMBC NMR, $CDCl_3$ of compound **S8**

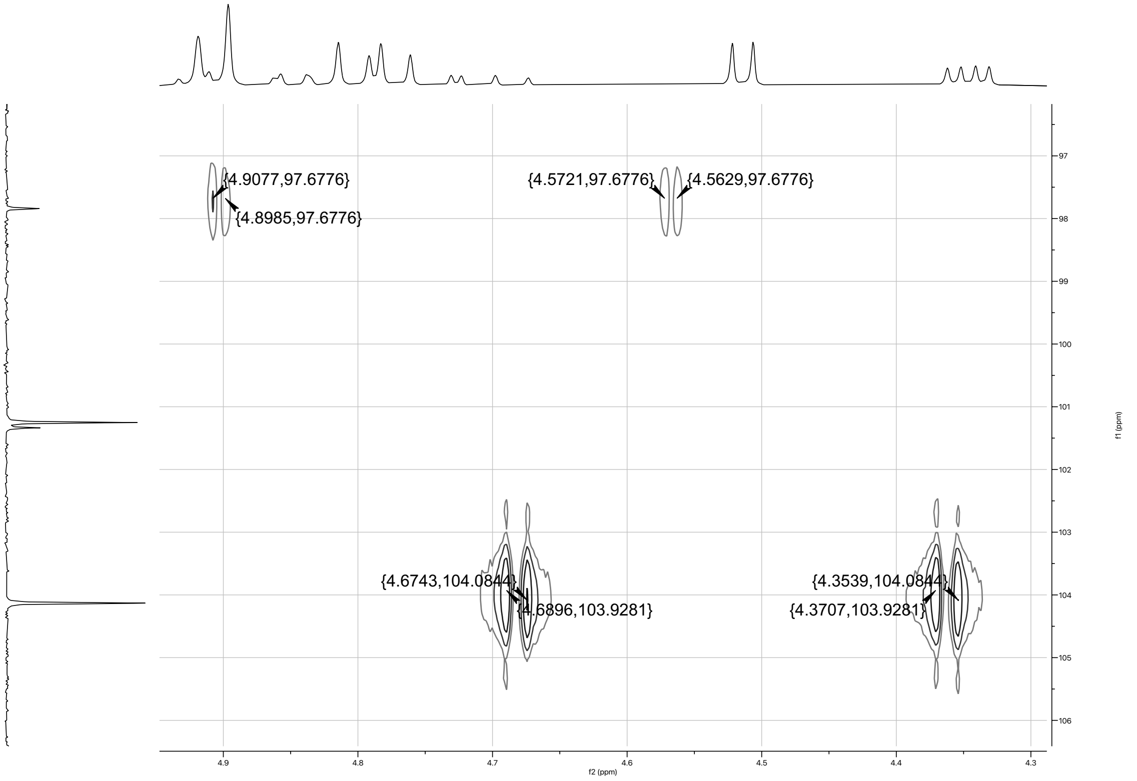

Supplementary Figure 29. HMBC-GATED NMR, $CDCl_3$ of compound **S8**

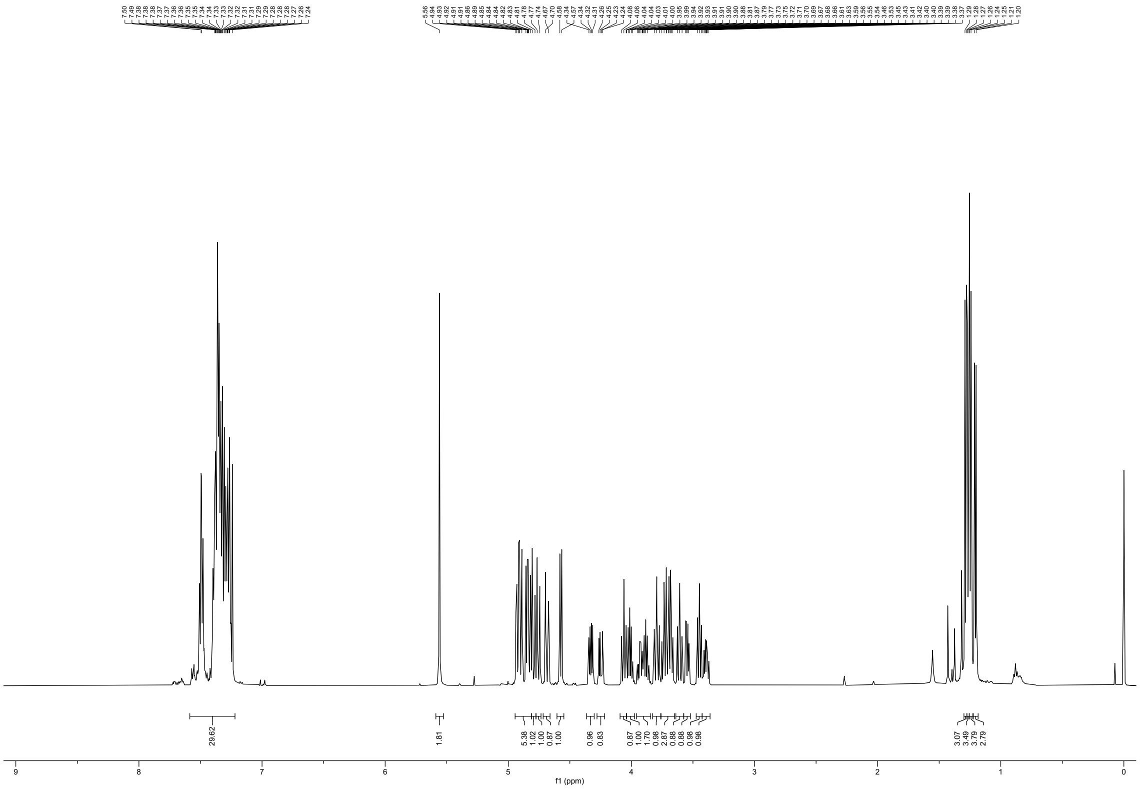

Supplementary Figure 30. ^{1}H-NMR, 500 MHz, $CDCl_3$ of compound **S12**

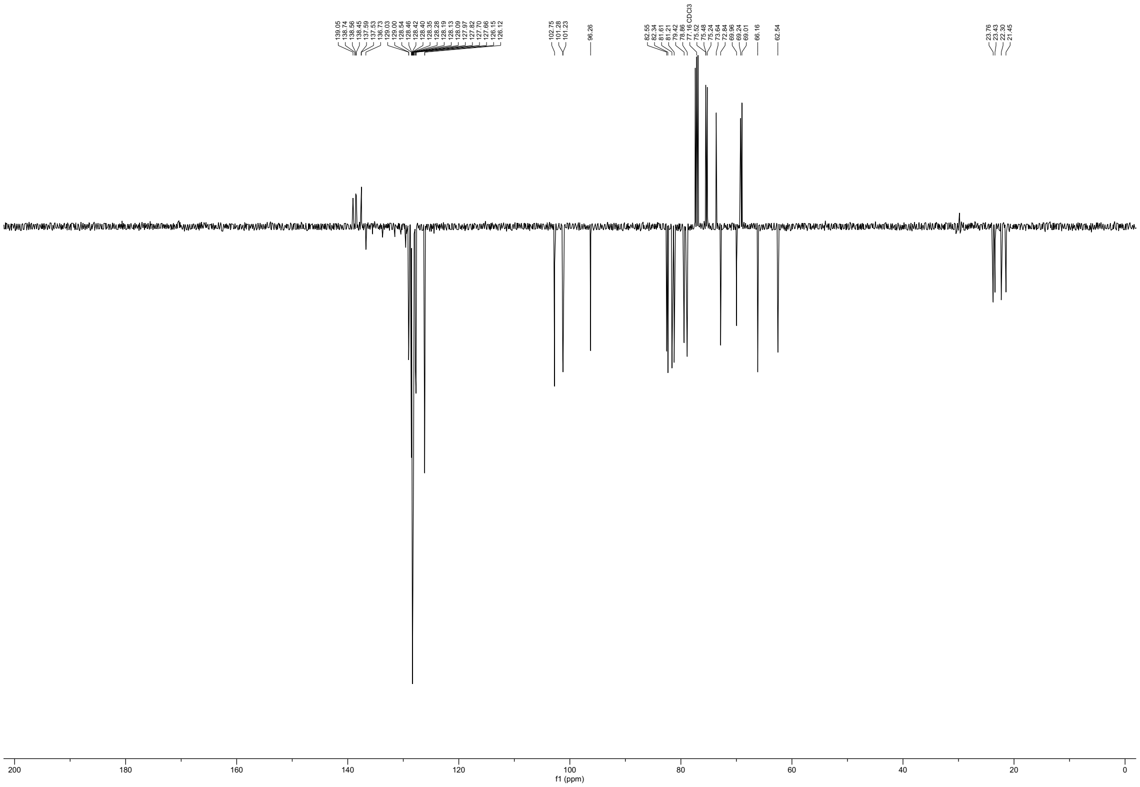

Supplementary Figure 31. ^{13}C-NMR, 126 MHz, $CDCl_3$ of compound **S12**

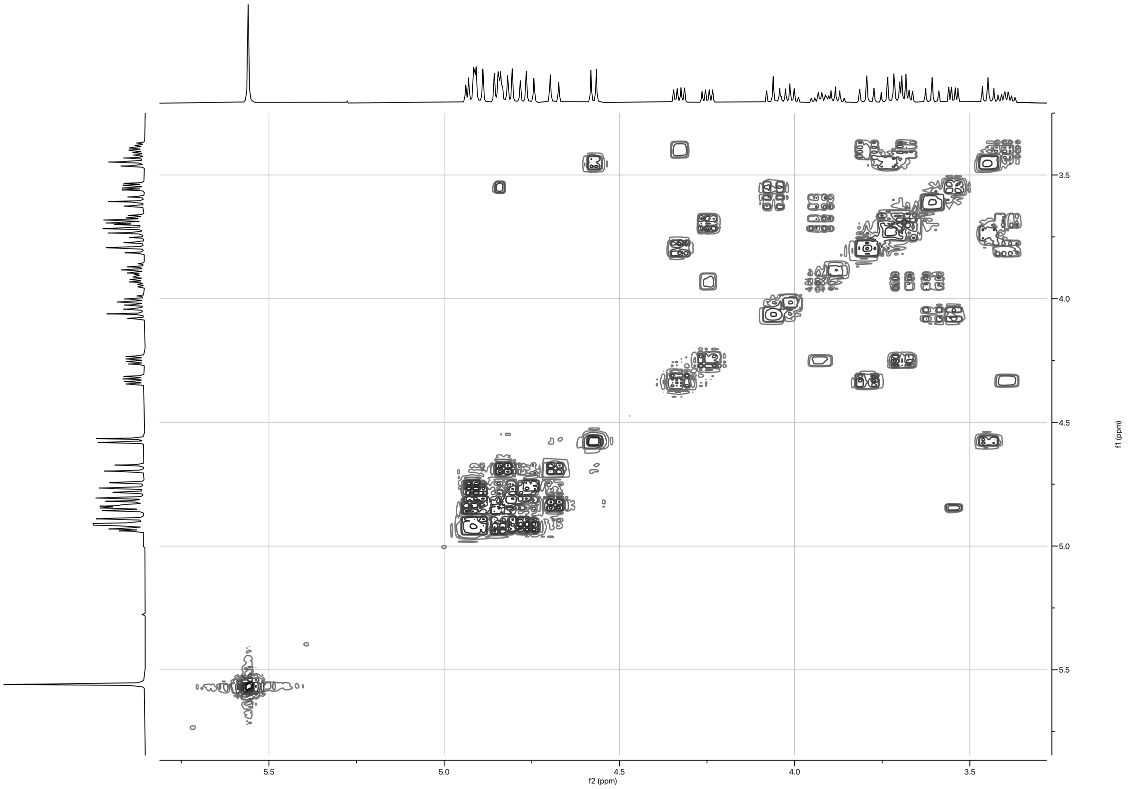

Supplementary Figure 32. HH-COSY NMR, $CDCl_3$ of compound **S12**

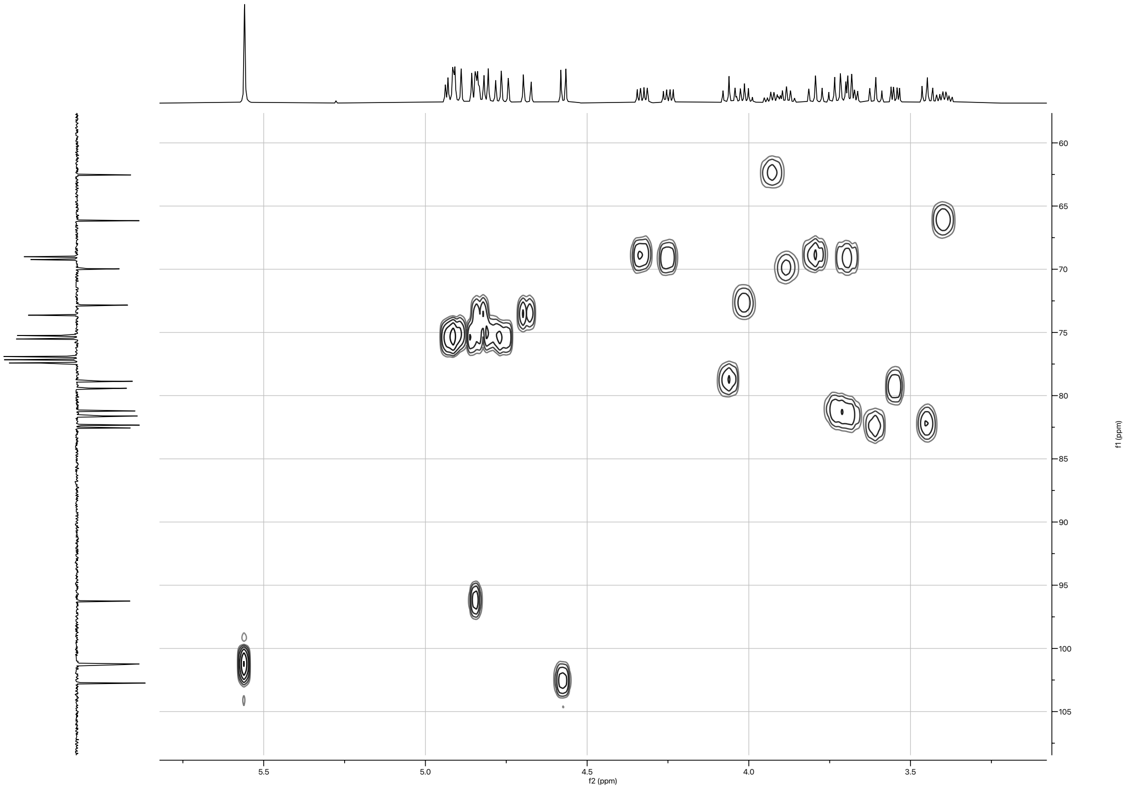

Supplementary Figure 33. HSQC NMR, $CDCl_3$ of compound **S12**

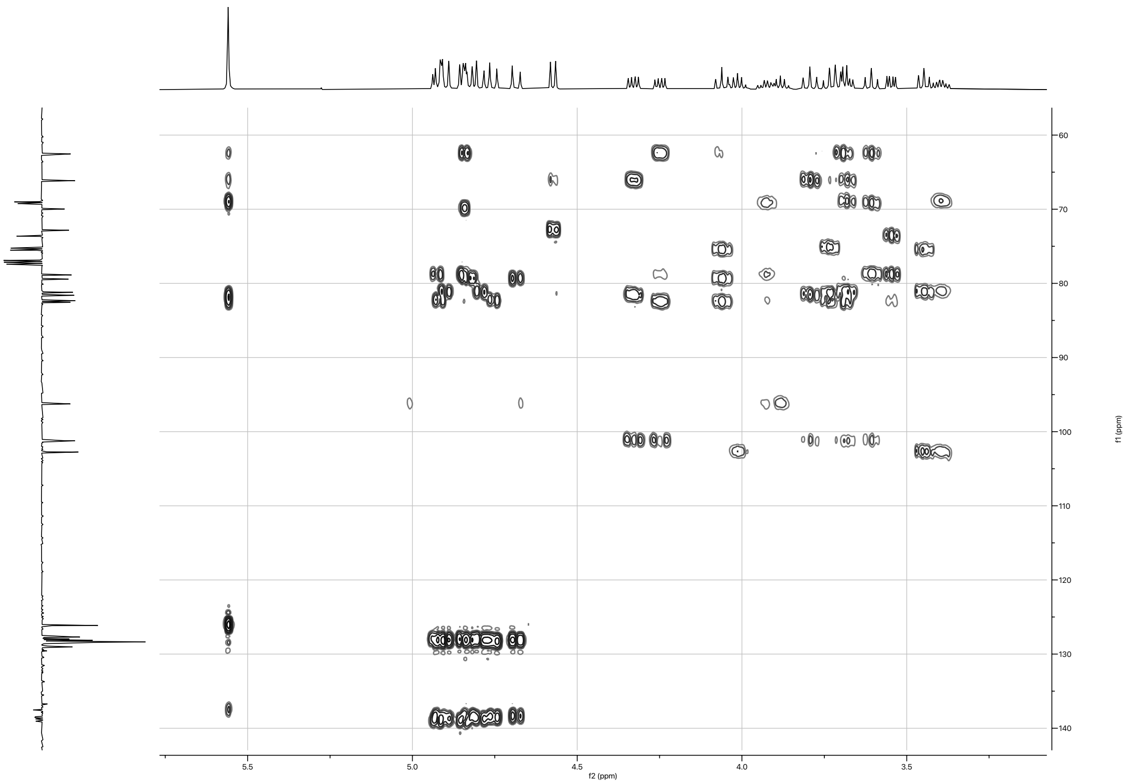

Supplementary Figure 34. HMBC NMR, $CDCl_3$ of compound **S12**

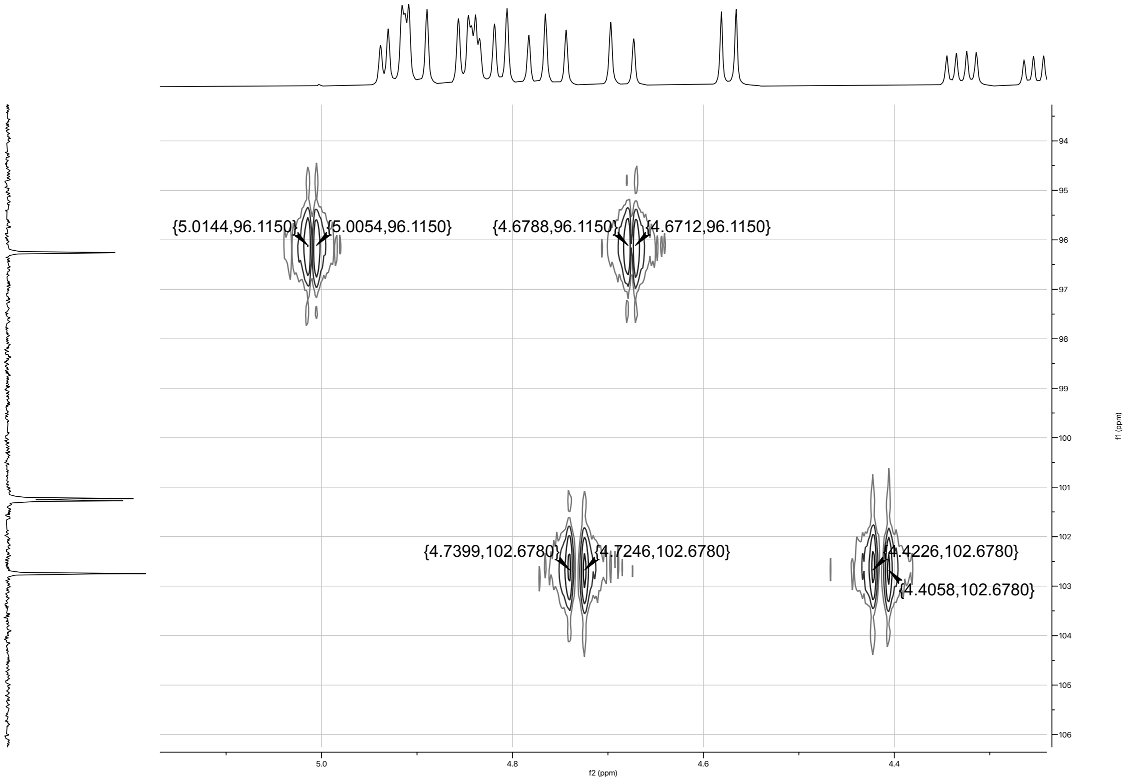

Supplementary Figure 35. HMBC-GATED NMR, $CDCl_3$ of compound **S12**

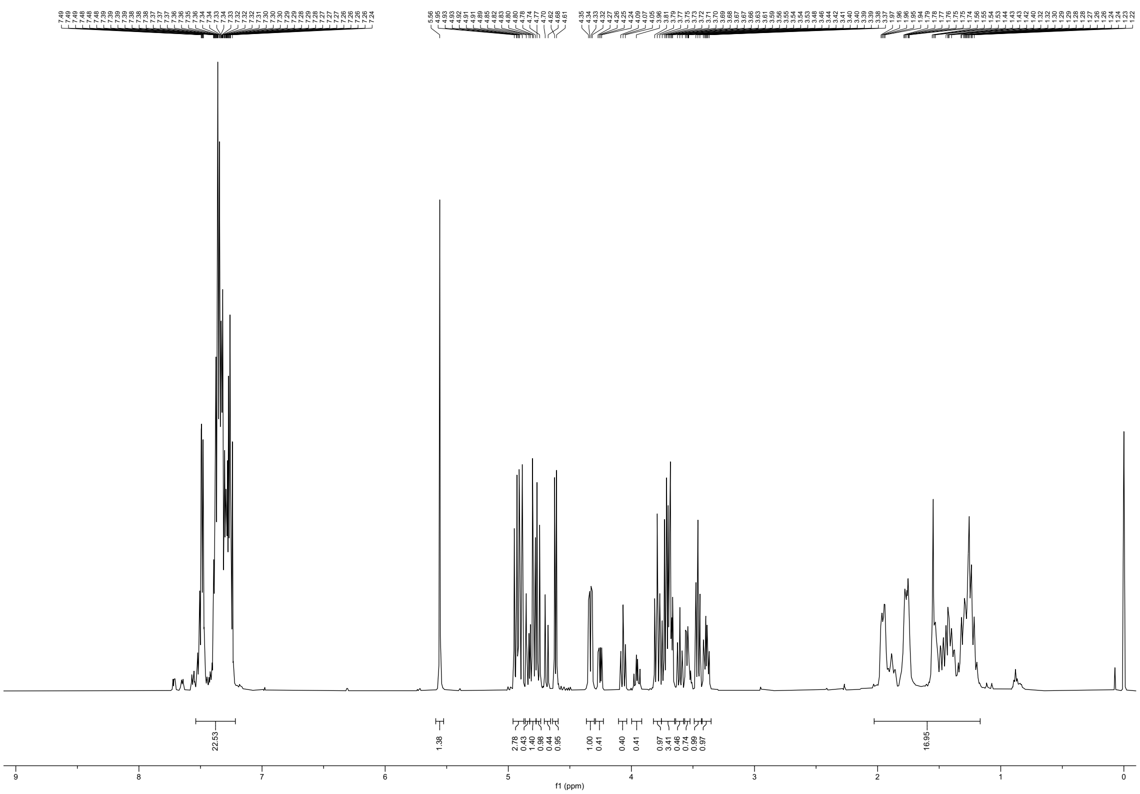

Supplementary Figure 36. ^{1}H-NMR, 500 MHz, $CDCl_3$ of compound **S13**

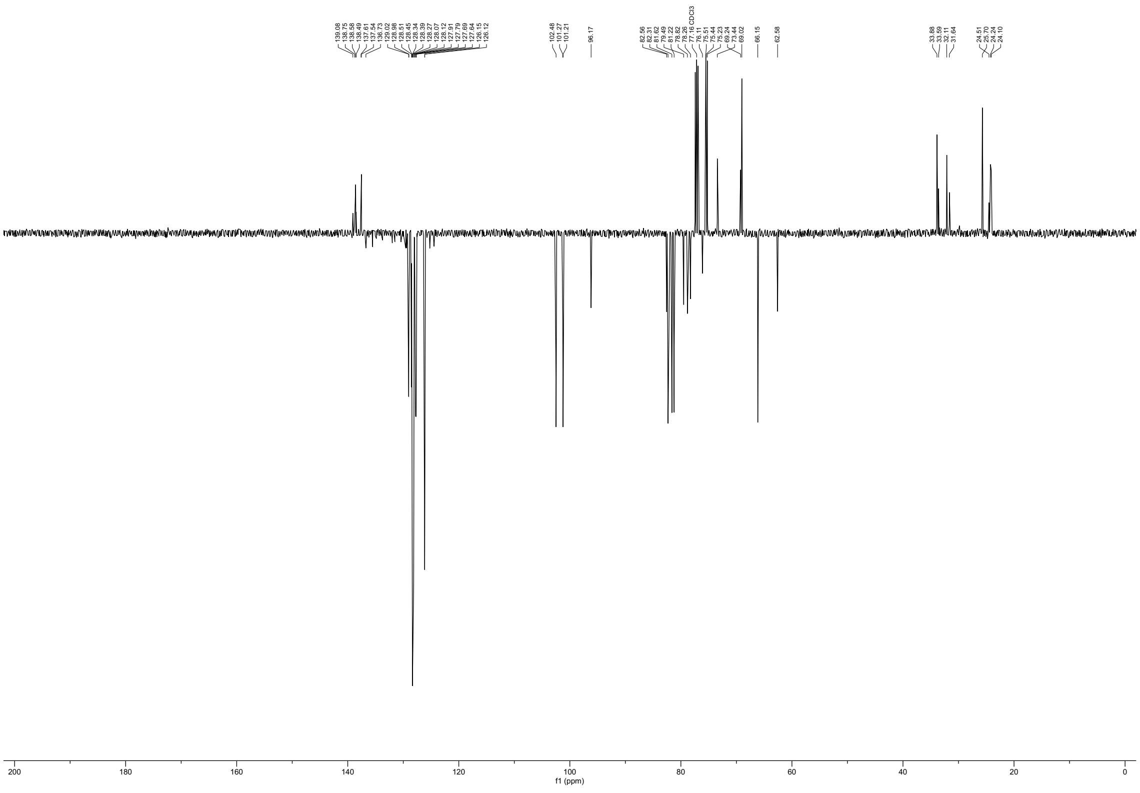

Supplementary Figure 37. ^{13}C-NMR, 126 MHz, $CDCl_3$ of compound **S13**

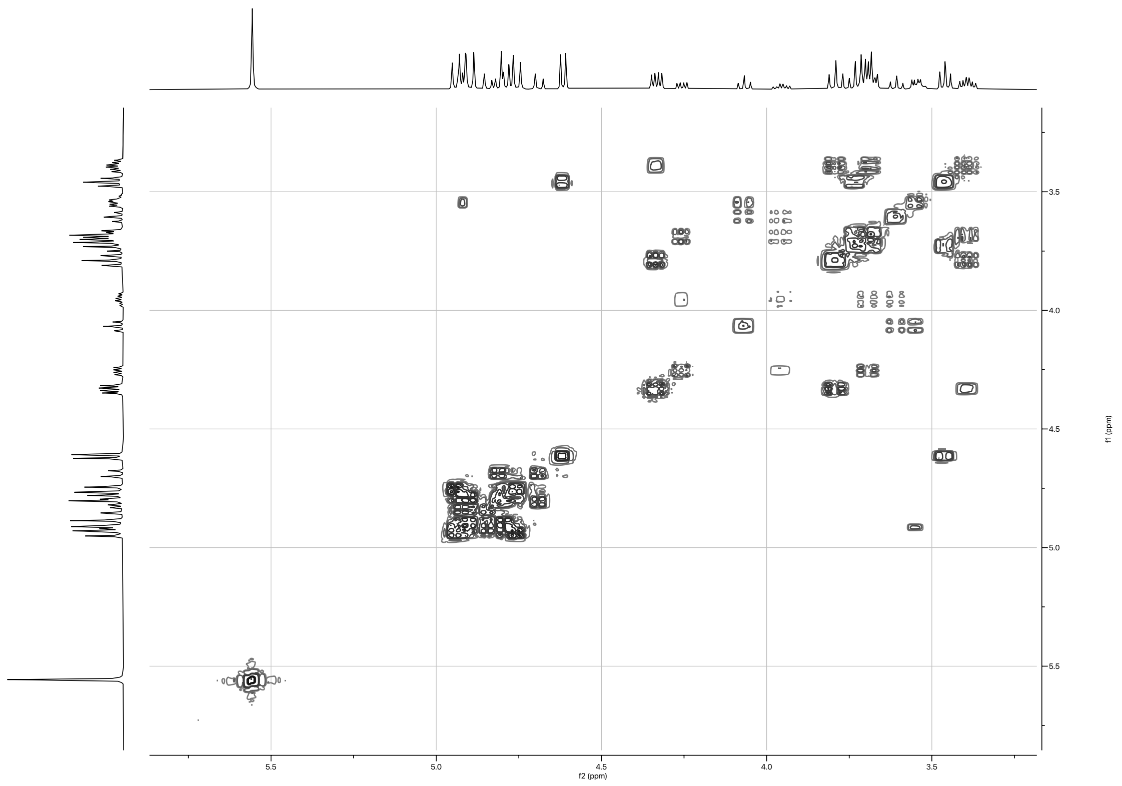

Supplementary Figure 38. HH-COSY NMR, $CDCl_3$ of compound **S13**

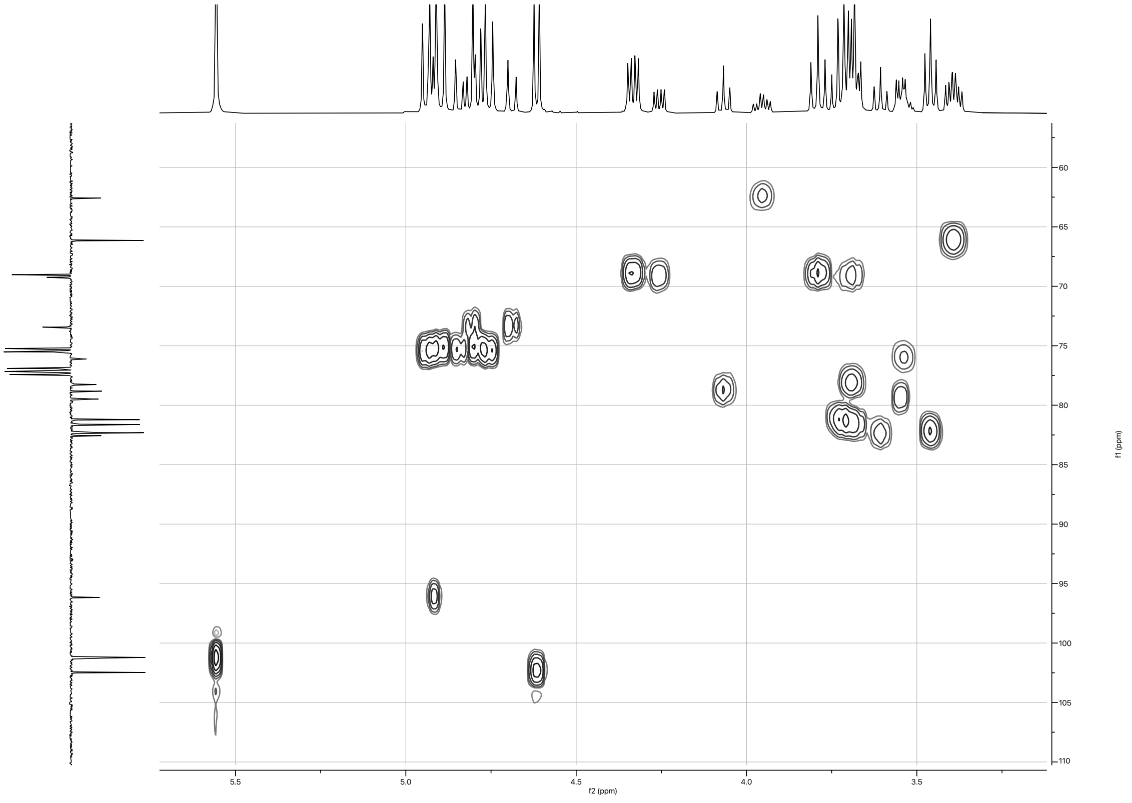

Supplementary Figure 39. HSQC NMR, $CDCl_3$ of compound **S13**

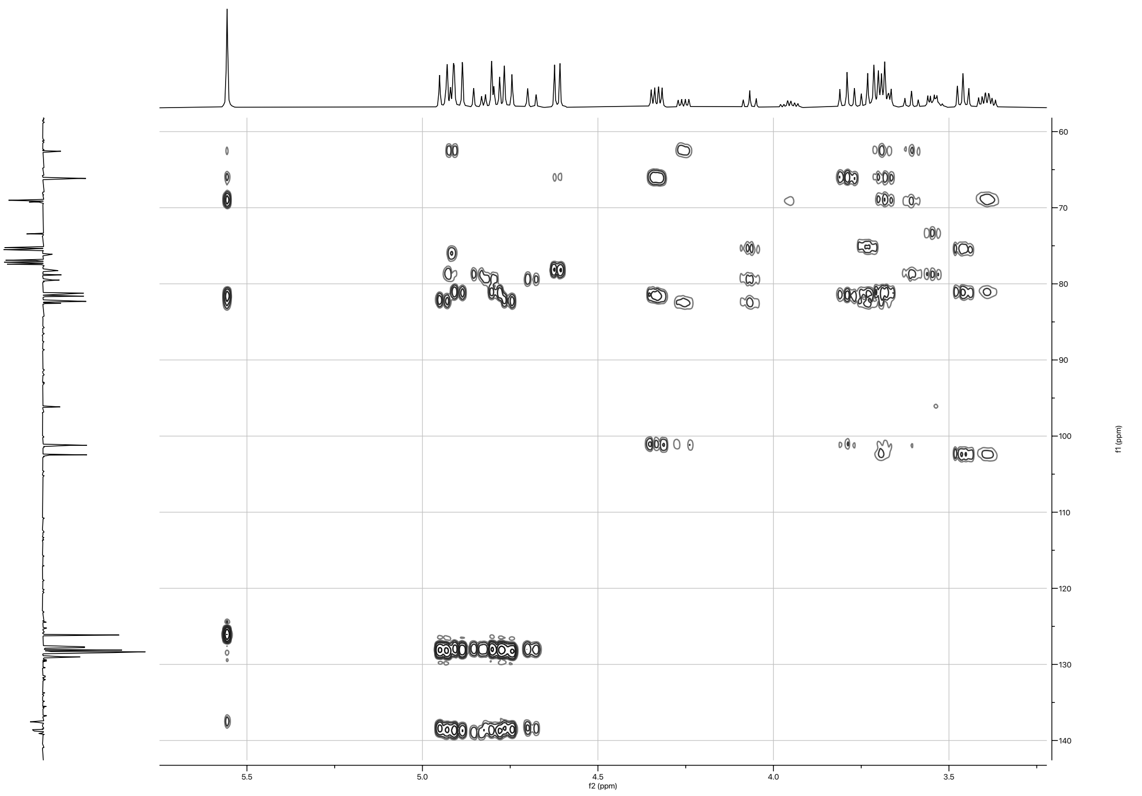

Supplementary Figure 40. HMBC NMR, $CDCl_3$ of compound **S13**

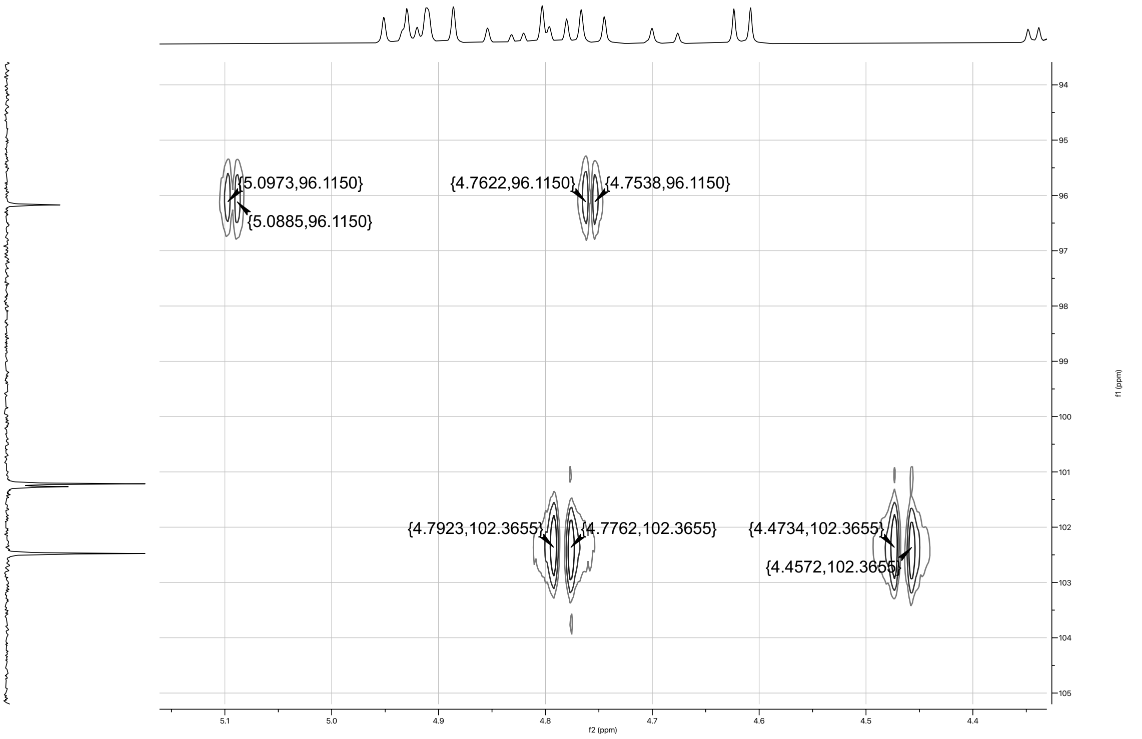

Supplementary Figure 41. HMBC-GATED NMR, $CDCl_3$ of compound **S13**

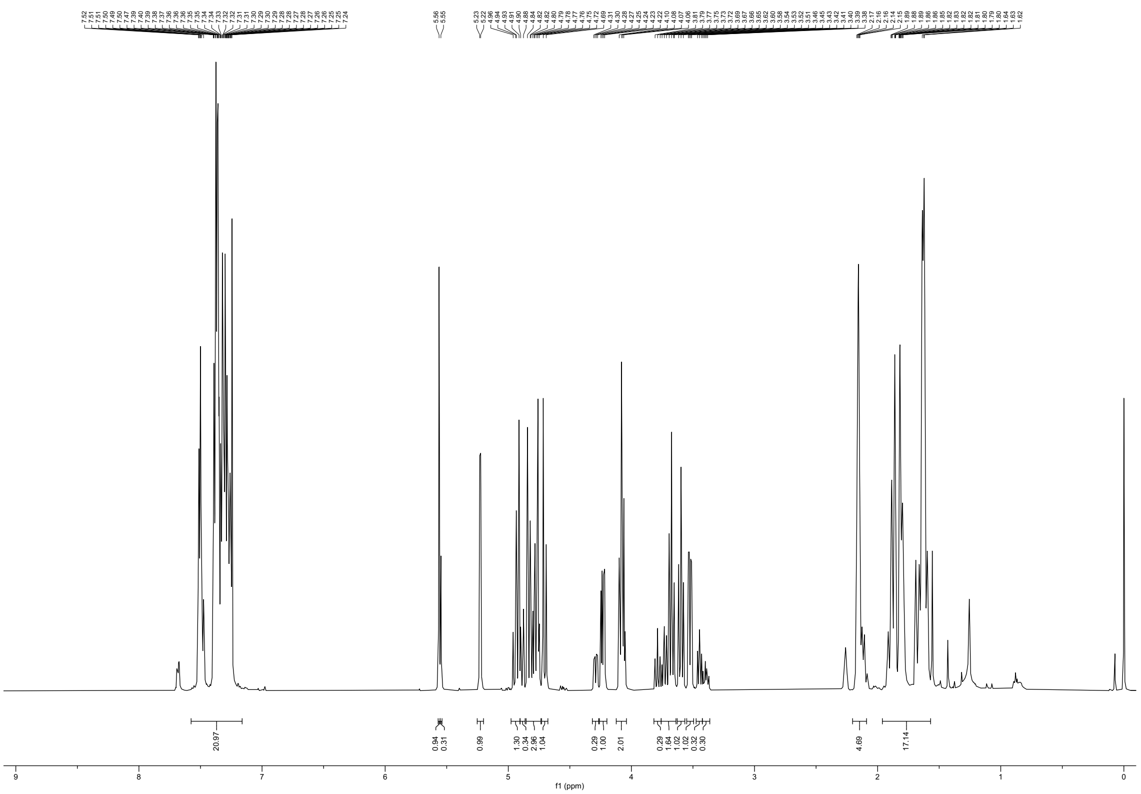

Supplementary Figure 42. ^{1}H-NMR, 500 MHz, $CDCl_3$ of compound **S14**

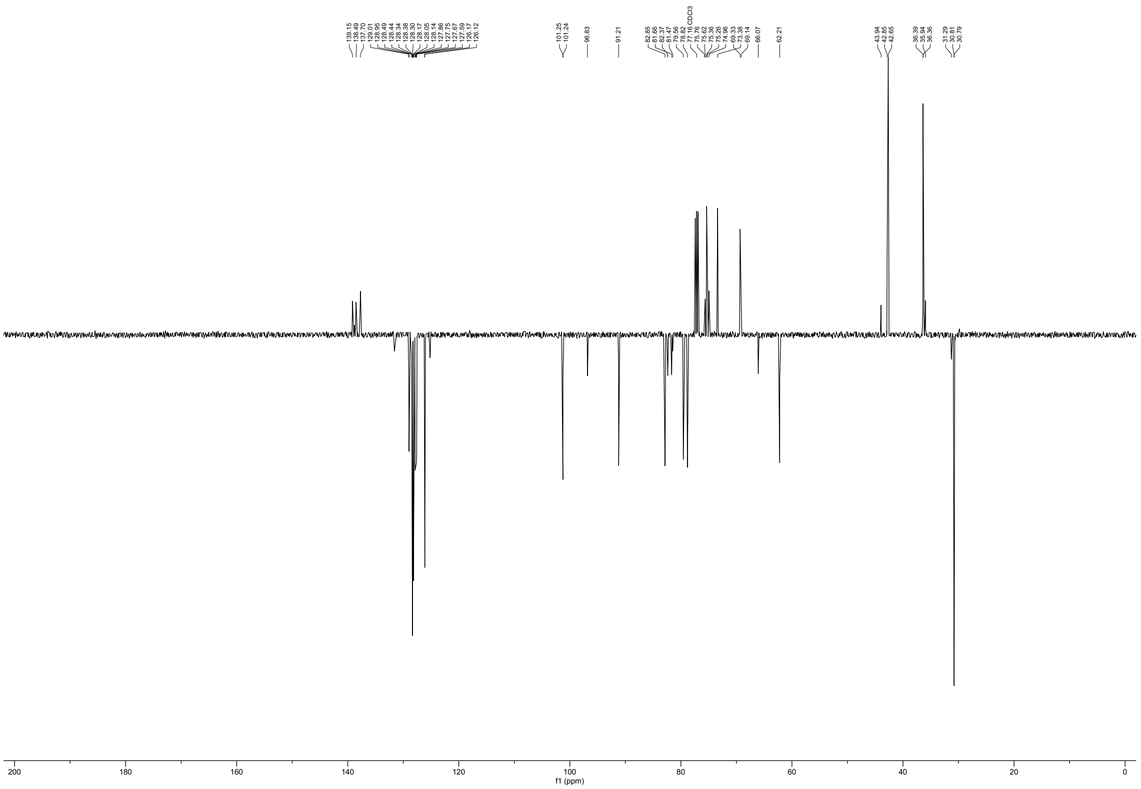

Supplementary Figure 43. ^{13}C-NMR, 126 MHz, $CDCl_3$ of compound **S14**

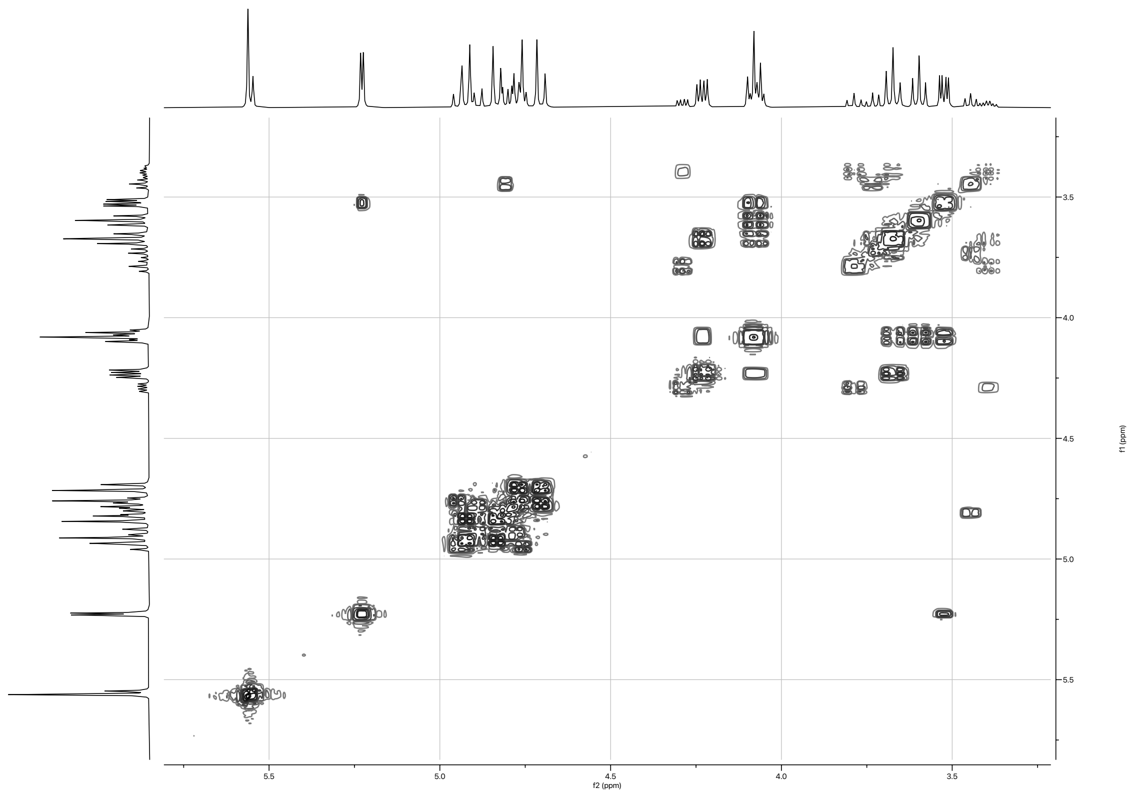

Supplementary Figure 44. HH-COSY NMR, $CDCl_3$ of compound **S14**

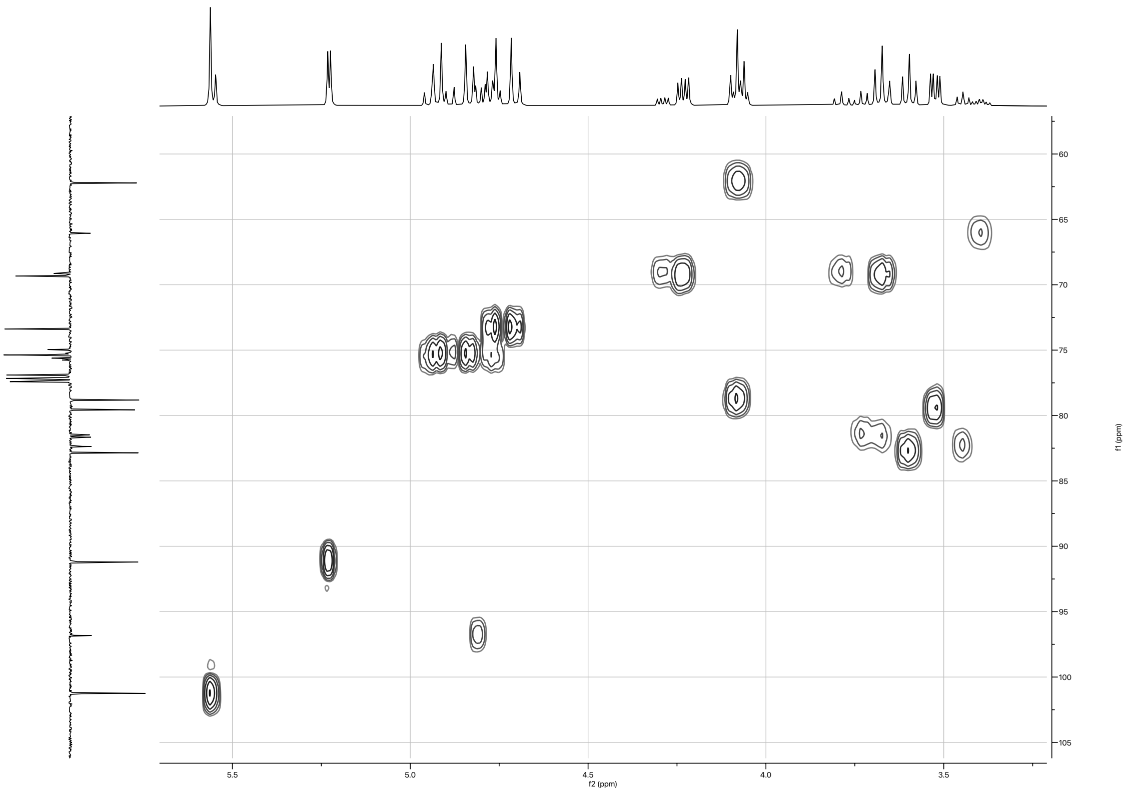

Supplementary Figure 45. HSQC NMR, $CDCl_3$ of compound **S14**

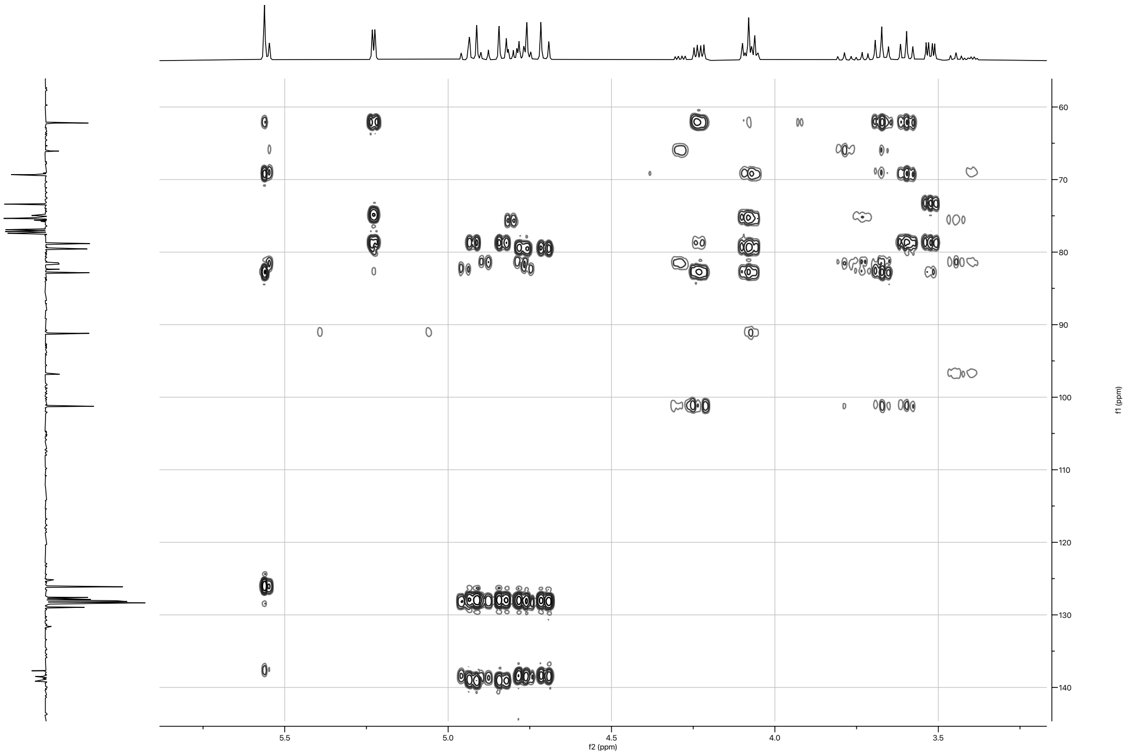

Supplementary Figure 46. HMBC NMR, $CDCl_3$ of compound **S14**

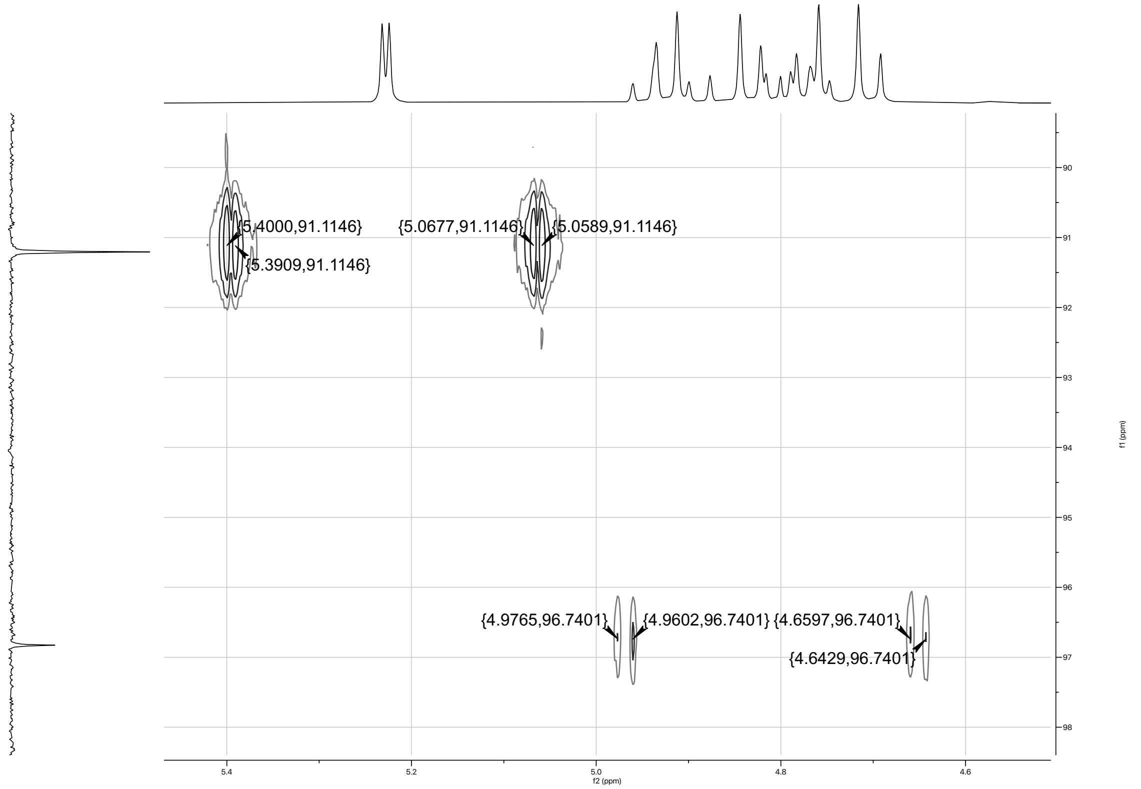

Supplementary Figure 47. HMBC-GATED NMR, $CDCl_3$ of compound **S14**

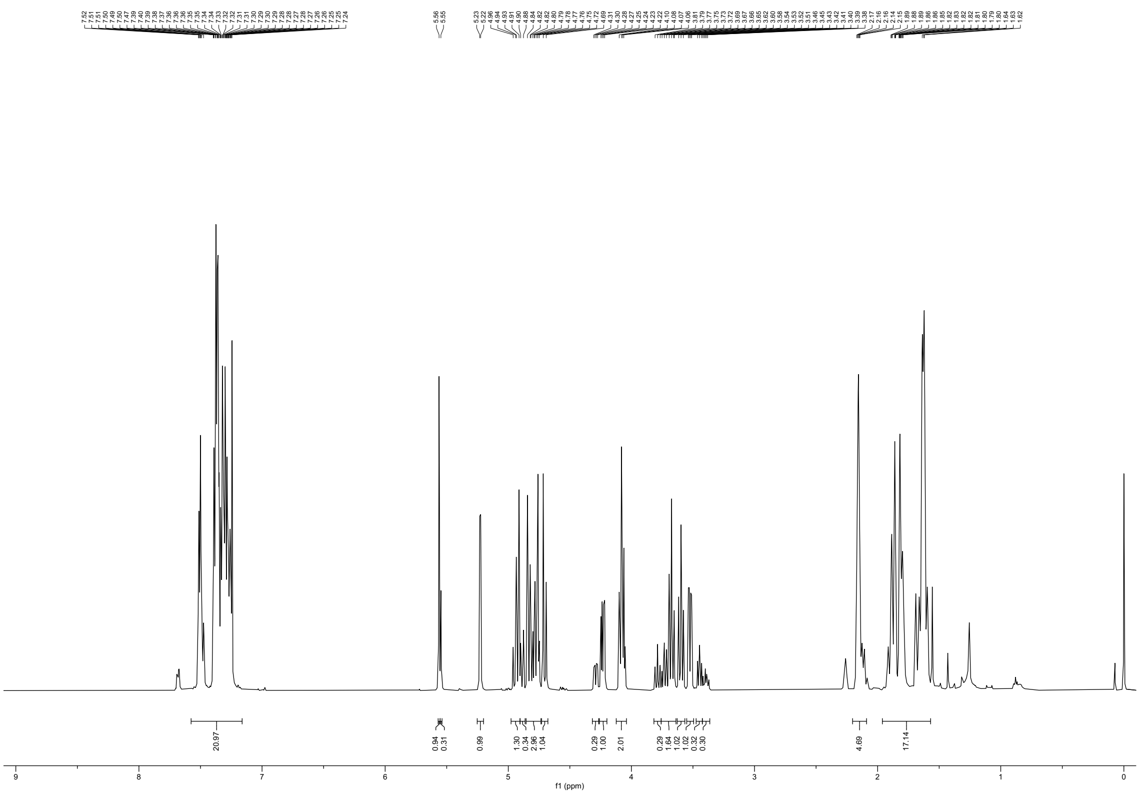

Supplementary Figure 48. ^{1}H-NMR, 500 MHz, $CDCl_3$ of compound **S15**

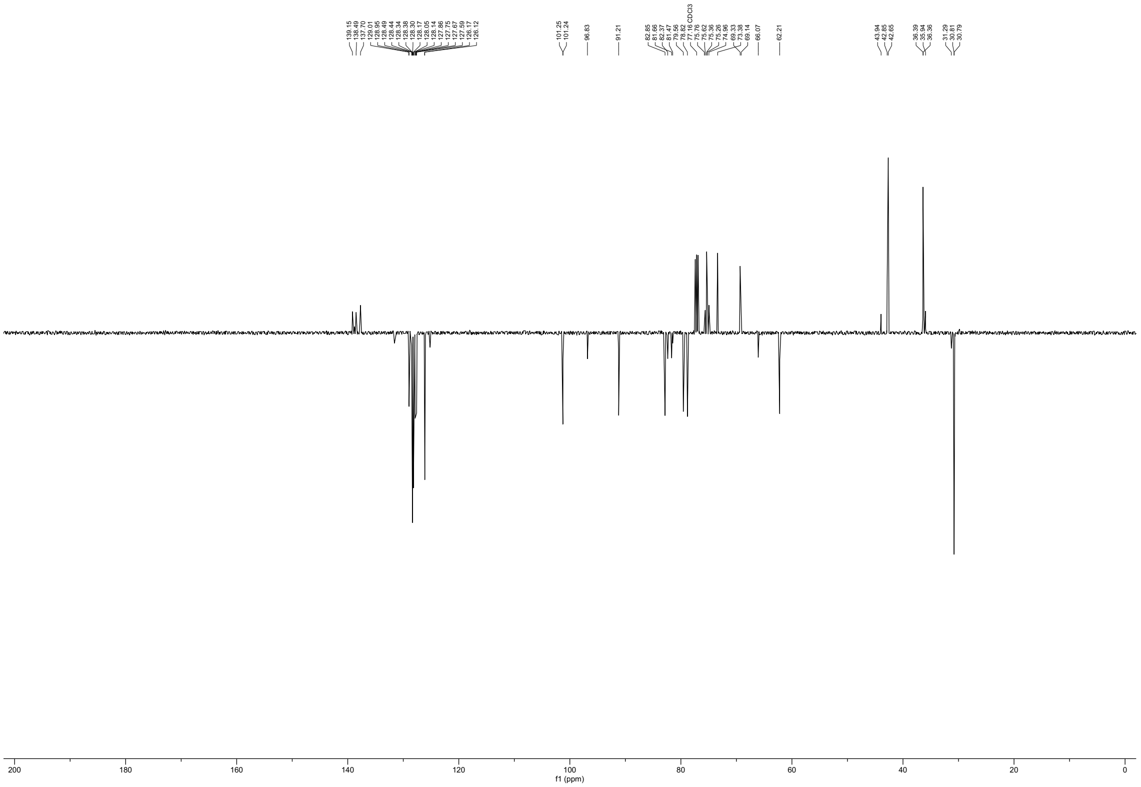

Supplementary Figure 49. ^{13}C-NMR, 126 MHz, $CDCl_3$ of compound **S15**

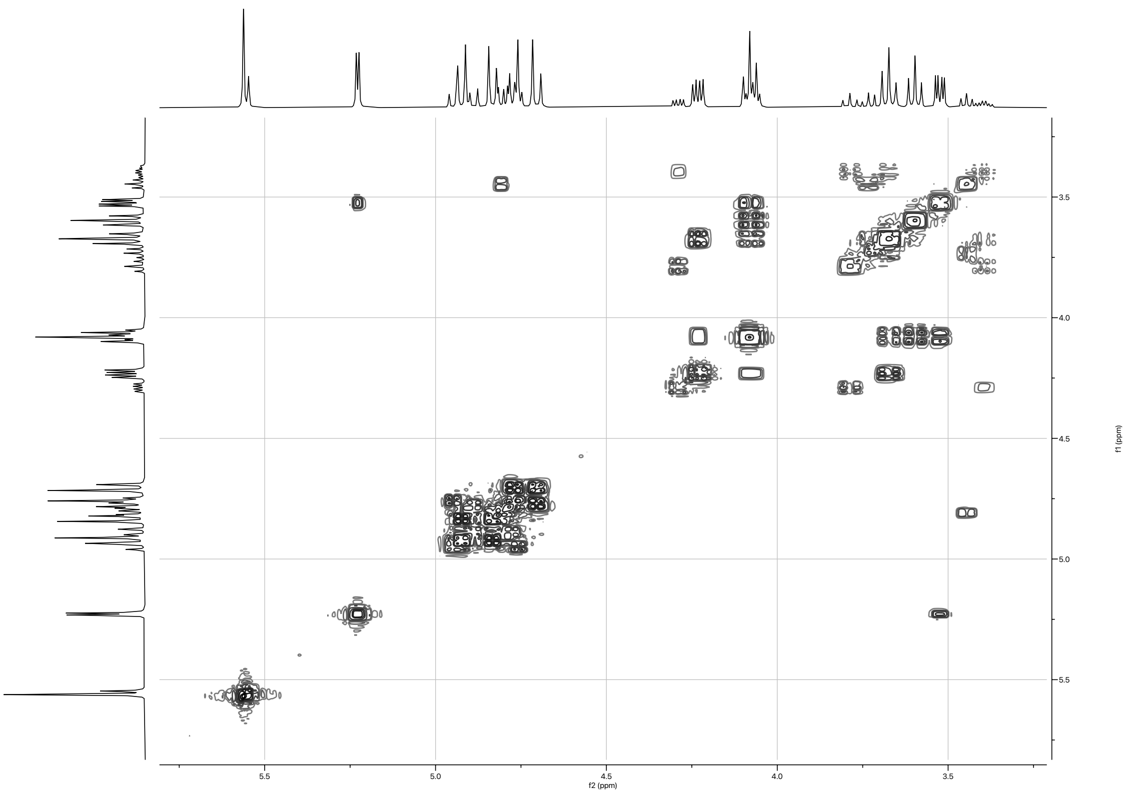

Supplementary Figure 50. HH-COSY NMR, $CDCl_3$ of compound **S15**

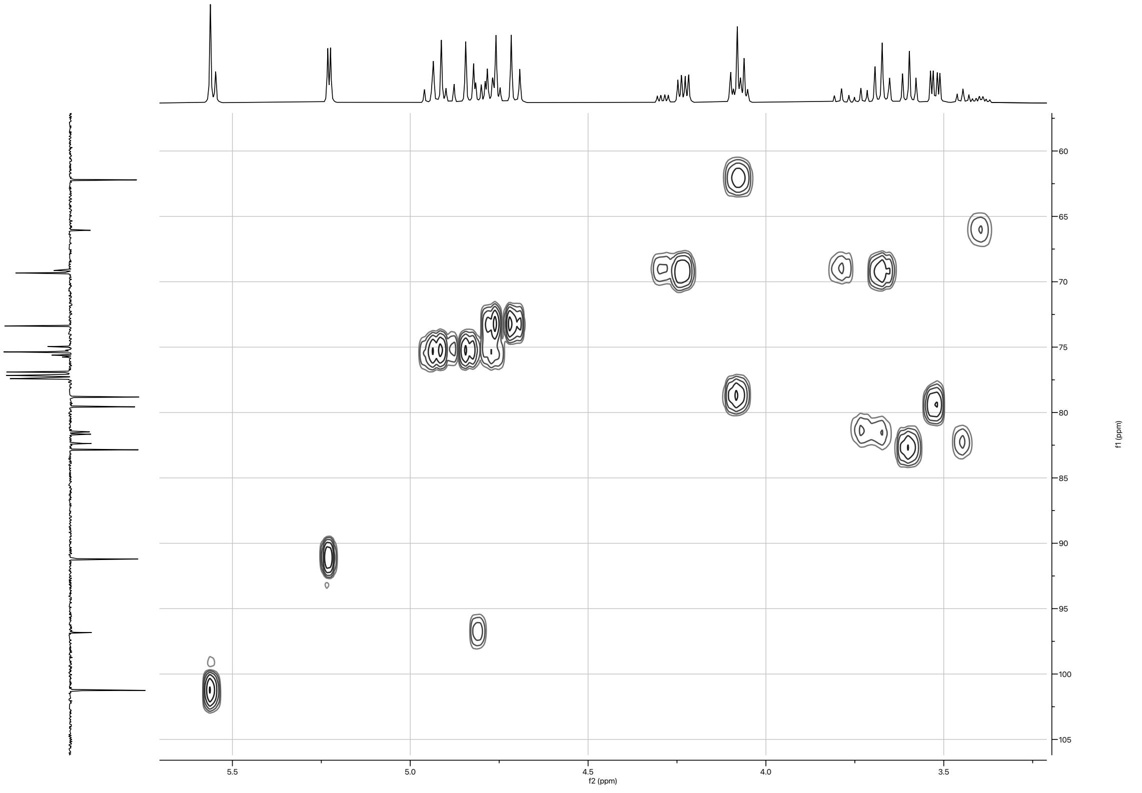

Supplementary Figure 51. HSQC NMR, $CDCl_3$ of compound **S15**

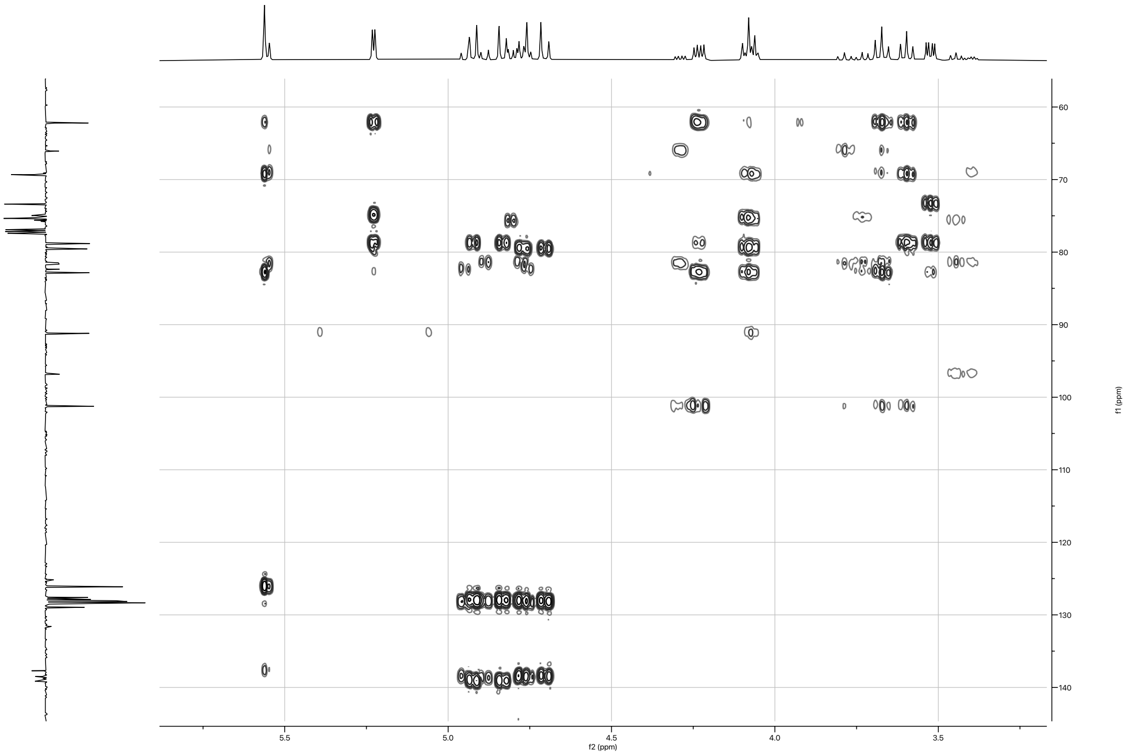

Supplementary Figure 52. HMBC NMR, $CDCl_3$ of compound **S15**

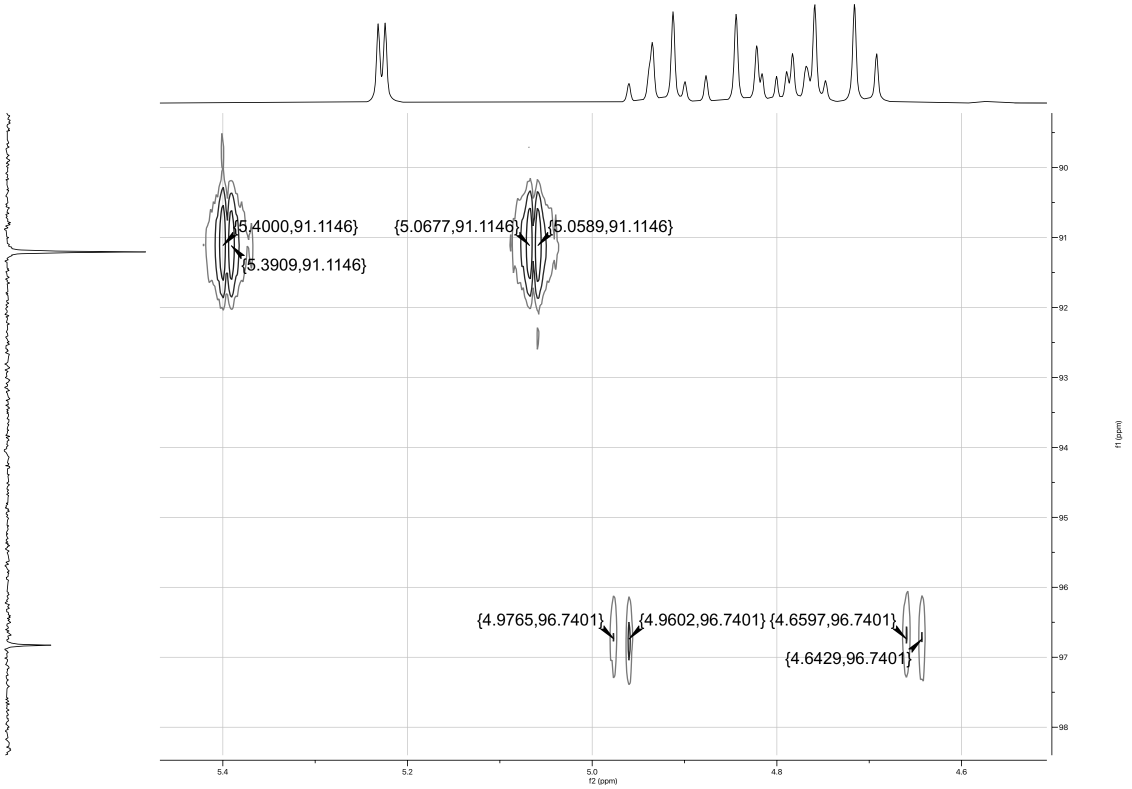

Supplementary Figure 53. HMBC-GATED NMR, $CDCl_3$ of compound **S15**
